# Imidazolium salts as an alternative for anti-Leishmania drugs: Oxidative and immunomodulatory activities

**DOI:** 10.3389/fimmu.2022.1096312

**Published:** 2023-01-17

**Authors:** Fernanda Giesel Baldissera, Tiago Fazolo, Matheus Brasil da Silva, Paulo Cesar de Santana Filho, Vinícius Demétrio da Silva, David Max Rivillo Perez, Joice Sandra Klitzke, Eduardo Giovanni de Oliveira Soares, Luiz Carlos Rodrigues Júnior, Alessandra Peres, Eliane Dallegrave, Kely Campos Navegantes-Lima, Marta Chagas Monteiro, Henri Stephan Schrekker, Pedro Roosevelt Torres Romão

**Affiliations:** ^1^ Laboratory of Cellular and Molecular Immunology, Universidade Federal de Ciências da Saúde de Porto Alegre (UFCSPA), Porto Alegre, RS, Brazil; ^2^ Graduate Program in Health Sciences, Universidade Federal de Ciências da Saúde de Porto Alegre (UFCSPA), Porto Alegre, RS, Brazil; ^3^ Laboratory of Technological Processes and Catalysis, Institute of Chemistry, Universidade Federal do Rio Grande do Sul (UFRGS), Porto Alegre, RS, Brazil; ^4^ Graduate Program in Biosciences, Universidade Federal de Ciências da Saúde de Porto Alegre (UFCSPA), Porto Alegre, RS, Brazil; ^5^ Graduate Program in Pharmaceutical Science, Graduate Program in Neuroscience and Cellular Biology, Faculty of Pharmacy, Universidade Federal do Pará, Belém, PA, Brazil

**Keywords:** imidazolium salt, *L. amazonensis*, *L. infantum chagasi*, oxygen reactive species, leishmanicidal activity

## Abstract

In this study we explored the previously established leishmanicidal activity of a complementary set of 24 imidazolium salts (IS), 1-hexadecylimidazole (C_16_Im) and 1-hexadecylpyridinium chloride (C_16_PyrCl) against *Leishmania (Leishmania) amazonensis* and *Leishmania (Leishmania) infantum chagasi*. Promastigotes of *L. amazonensis* and *L. infantum chagasi* were incubated with 0.1 to 100 μM of the compounds and eight of them demonstrated leishmanicidal activity after 48 h – C_10_MImMeS (IC_50_ *_L. amazonensis_* = 11.6), C_16_MImPF_6_(IC_50_ *_L. amazonensis_* = 6.9), C_16_MImBr (IC_50_ *_L. amazonensis_* = 6), C_16_M_2_ImCl (IC_50_ *_L. amazonensis_* = 4.1), C_16_M_4_ImCl (IC_50_ *_L. amazonensis_* = 1.8), (C_10_)_2_MImCl (IC_50_ *_L. amazonensis_* = 1.9), C_16_Im (IC_50_ *_L. amazonensis_* = 14.6), and C_16_PyrCl (IC_50_ *_L. amazonensis_* = 4).The effect of IS on reactive oxygen species production, mitochondrial membrane potential, membrane integrity and morphological alterations of promastigotes was determined, as well as on *L. amazonensis*-infected macrophages. Their cytotoxicity against macrophages and human erythrocytes was also evaluated. The IS C_10_MImMeS, C_16_MImPF_6_, C_16_MImBr, C_16_M_2_ImCl, C_16_M_4_ImCl and (C_10_)_2_MImCl, and the compounds C_16_Im and C_16_PyrCl killed and inhibited the growth of promastigote forms of *L. amazonensis* and *L. infantum chagasi* in a concentration-dependent manner, contributing to a better understanding of the structure-activity relationship of IS against *Leishmania*. These IS induced ROS production, mitochondrial dysfunction, membrane disruption and morphological alterations in infective forms of *L. amazonensis* and killed intracellular amastigote forms in very low concentrations (IC_50 amastigotes_ ≤ 0.3), being potential drug candidates against *L. amazonensis*.

## 1 Introduction

Leishmaniasis are endemic in 102 countries or territories with around 1.3 million new cases and 20.000 to 30.000 deaths yearly (1). Brazil is one of the countries with the highest incidence of leishmaniasis (2). *Leishmania (Leishmania) amazonensis* is an important etiological agent of cutaneous, anergic diffuse cutaneous and less commonly mucocutaneous leishmaniasis, especially in South America (3, 4), and *Leishmania (Leishmania) infantum chagasi* causes visceral leishmaniasis, mainly in the Mediterranean Basin, Middle East, Central Asia and South America (4).

**Figure 1:**
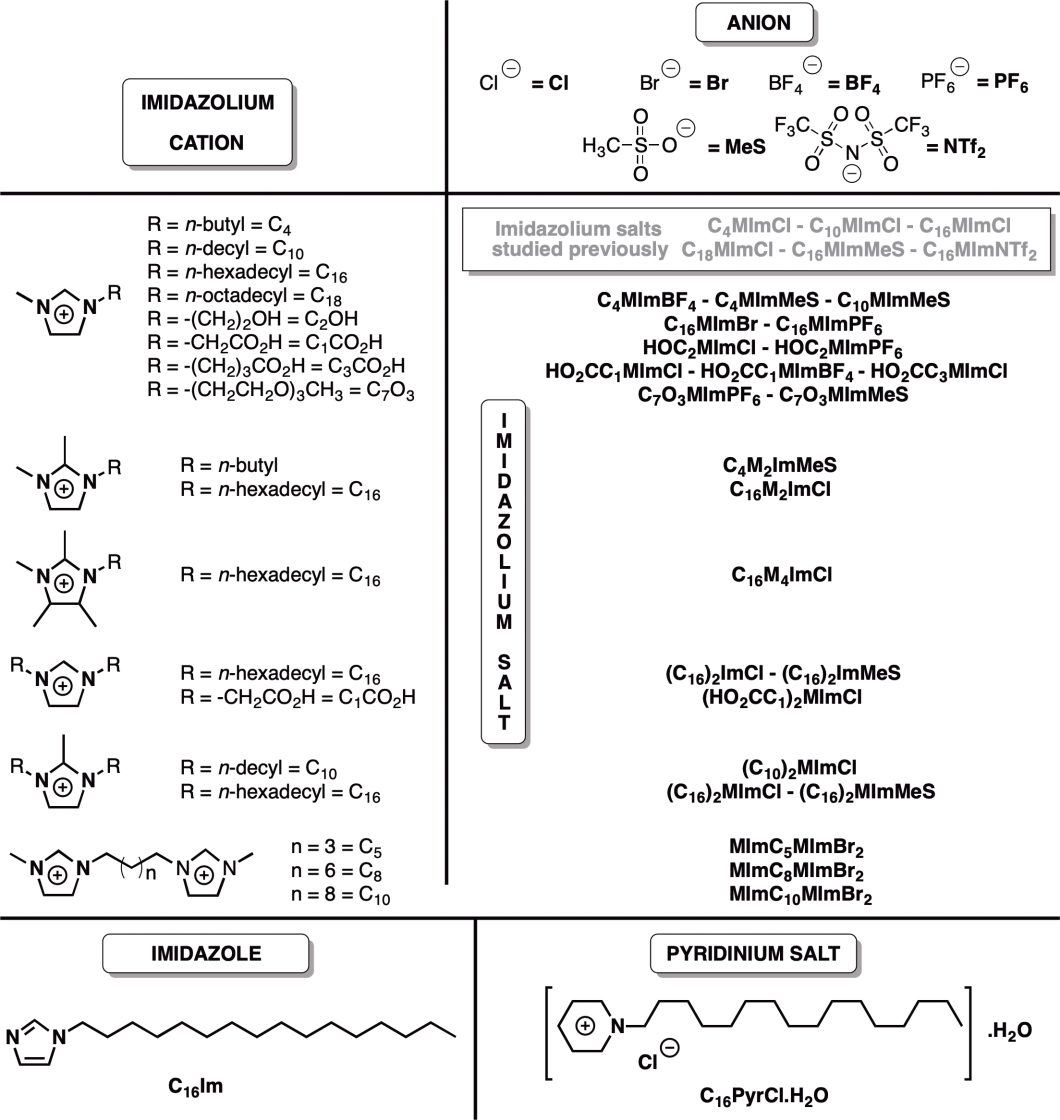
Chemical structures of the imidazolium salts, C_16_Im and C_16_PyrCl screened for anti-*Leishmania* activity tested in this work, and imidazolium salts studied previously for anti-*Leishmania* activity (ref 13). The compounds were given sample codes that contain information on their chemical structures. For instance, for C_18_MImCl: (1) C_18_ = *N*-octadecyl substituent; (2) M = *N*-methyl substituent; (3) Im = imidazolium cation; and (4) Cl = chloride anion.

Currently, there is no vaccine for leishmaniasis, and their treatment is based on very toxic and poorly tolerated drugs, such as pentavalent antimonials and amphotericin B (5). Azole antifungals presented leishmanicidal activity against some *Leishmania* species (6). Moreover, various biological activities have been described for the cationic equivalent of imidazoles, imidazolium salts (IS), including antimicrobial (7), antifungal (8, 9), larvicidal (10), antitumoral, antioxidant and others (11). The IS present high structural variability and tunable interactions with biomolecules like phospholipids and proteins, especially because their amphiphilic nature (11), becoming potential candidates in medicinal chemistry (12). Recently, our group described for the first time the leishmanicidal activity of IS (13). Within the tested IS, 1-methyl-3-octadecylimidazolium chloride (C_18_MImCl), 1-hexadecyl-3-methylimidazolium methanesulfonate (C_16_MImMeS), 1-hexadecyl-3-methylimidazolium chloride (C_16_MImCl), 1-decyl-3-methylimidazolium chloride (C_10_MImCl) and 1-hexadecyl-3-methylimidazolium bis(trifluoromethylsulfonyl)imide (C_16_MImNTf_2_) were identified as potent agents against both promastigote and amastigote forms of *L. amazonensis*. Here, we further explored this leishmanicidal action with a structurally complementary set of 24 IS, 1-hexadecylimidazole (C_16_Im) and 1-hexadecylpyridinium chloride (C_16_PyrCl) (**Figure 1**) to (i) investigate the structure-activity relationship of these molecules on promastigote and amastigote forms of *L. amazonensis* and promastigote forms of *L. infantum chagasi*, (ii) identify mortality pathways, and (iii) evaluate cytotoxicity.

## 2 Materials and methods

### 2.1 Imidazolium salts and reference compounds

1,3-Didecyl-2-methylimidazolium chloride ((C_10_)_2_MImCl) (CJC China JIE Chemical), 1-hexadecylpyridinium chloride monohydrate (C_16_PyrCl.H_2_O) (Sigma-Aldrich), and 1-(2-hydroxyethyl)-3-methylimidazolium chloride (HOC_2_MImCl) (IOLITEC) were purchased and used as received.

1-Butyl-3-methylimidazolium methanesulfonate (C_4_MImMeS) (14), 1-decyl-3-methylimidazolium methanesulfonate (C_10_MImMeS) (15), 1-hexadecyl-3-methylimidazolium bromide (C_16_MImBr) (16), 1-butyl-3-methylimidazolium tetrafluoroborate (C_4_MImBF_4_) (14), 1,10-bis(methylimidazolium-1-yl) decane bromide (MImC_10_MImBr_2_) (16), 1-triethylene glycol monomethyl ether-3-methylimidazolium hexafluorophosphate (C_7_O_3_MImPF_6_) (15), 1-triethylene glycol monomethyl ether-3-methylimidazolium methanesulfonate (C_7_O_3_MImMeS) (15), 1-butyl-2,3-dimethylimidazolium methanesulfonate (C_4_M_2_ImMeS) (17), 1-hexadecylimidazole (C_16_Im) (18), 1,3-dihexadecylimidazolium chloride ((C_16_)_2_ImCl) (19), 1-(3-hydroxypropyl)-3-methylimidazolium hexafluorophosphate (HOC_3_MImPF_6_) (20), 1-carboxymethyl-3-methylimidazolium chloride (HO_2_CC_1_MImCl) (21), 1,3-bis(carboxymethyl)imidazolium chloride ((HO_2_CC_1_)_2_ImCl) (21), 1-carboxymethyl-3-methylimidazolium tetrafluoroborate (HO_2_CC_1_MImBF_4_) (22), 1-carboxypropyl-3-methylimidazolium chloride (HO_2_CC_3_MImCl) (23), 1-hexadecyl-3-methylimidazolium hexafluorophosphate (C_16_MImPF_6_) (24), 1-hexadecyl-2,3,4,5-tetramethylimidazolium chloride (C_16_M_4_ImCl) (25), decyl methanesulfonate (C_10_MeS) (15), hexadecyl methanesulfonate (C_16_MeS) (26), butyl methanesulfonate (C_4_MeS) (14), 1-hexadecyl-2-methylimidazole (C_16_MIm) (27), and triethylene glycol monomethyl ether methanesulfonate (C_7_O_3_MeS) (15) were synthesized according to previously reported procedures. C_4_MeS, C_10_MeS, C_16_MeS, C_16_Im, C_16_MIm, and C_7_O_3_MeS were used as source for the syntheses of C_4_MImMeS, C_4_M_2_ImMeS, C_10_MImMeS, (C_16_)_2_ImCl, C_7_O_3_MImMeS, 1,3-dihexadecylimidazolium methanesulfonate (C_16_)_2_ImMeS, 1,3-dihexadecyl-2-methylimidazolium chloride ((C_16_)_2_MImCl), and 1,3-dihexadecyl-2-methylimidazolium methanesulfonate ((C_16_)_2_MImMeS). Literature procedures were used as basis for the synthesis of 1-hexadecyl-2,3-dimethylimidazolium chloride (C_16_M_2_ImCl) (15), 1,5-bis(methylimidazolium-1-yl) pentane bromide (MImC_5_MImBr_2_) (16), 1,8-bis(methylimidazolium-1-yl) octane bromide (MImC_8_MImBr_2_) (16), (C_16_)_2_ImMeS (15), (C_16_)_2_MImCl (15) and (C_16_)_2_MImMeS (15), which are yet unpublished IS. See the **Supporting Information** for further details. Before use, the IS were dried under vacuum at 60°C for 5 h to remove residual water.

### 2.2 Parasite culture

*Leishmania (L.) amazonensis* (MHOM/BR/73/M2269 strain) and *Leishmania* (*L*.) *infantum chagasi* (MHOM/BR/74/PP75 strain) were cultured at 26 °C as previously described elsewhere (28). To avoid the loss of *in vitro* infectivity of promastigotes, samples of parasites were kept cryopreserved in liquid nitrogen soon after isolation of infected animals (BALB/c or hamsters, respectively). All experiments were performed using parasites with few passages of *in vitro* growth.

### 2.3 Anti-promastigote activity

The effect of IS, C_16_Im and C_16_PyrCl on *L. amazonensis* and *L. infantum chagasi* was evaluated as previously described (28). Promastigotes of *L. amazonensis* in the stationary phase were distributed in 96-well microplates (3 x 10^6^ cells/well) and incubated with M199 medium or 100 μM (screening concentration) of tested compound (IS, C_16_Im or C_16_PyrCl) for 44 h at 26 °C. Subsequently, 0.5 mg/mL of 3-(4,5-dimethylthiazol-2-yl)-2,5-diphenyltetrazolium bromide (MTT, Sigma-Aldrich^®^, St. Louis, MO, USA) was added and the incubations continued for an additional 4 h at 26 °C. The purple formazan product that was formed by the action of mitochondrial enzymes in living cells was solubilized by the addition of acidic isopropanol, and the absorbance at 570 nm was measured using a microplate reader spectrophotometer EZ Read 400 from Biochrom. The compounds that caused 100% of *L. amazonensis* mortality in the screening test (C_10_MImMeS, C_16_MImPF_6_, C_16_MImBr, C_16_M_2_ImCl, C_16_M_4_ImCl, (C_10_)_2_MImCl, C_16_Im and C_16_PyrCl) were selected for additional tests against *L. amazonensis* and *L. infantum chagasi* at concentrations of 0.1 to 100 μM. The IS C_10_MImCl, C_16_MImCl and C_16_MImMeS, tested against *L. amazonensis* in our previous study (13), were tested here against promastigotes of *L. infantum chagasi*. The 50% inhibitory concentration (IC_50_) value for each compound was determined. The survival rate was calculated according to the formula: OD in treated group/OD of untreated group x 100. The 50% inhibitory concentration (IC_50_) value for each compound was determined by nonlinear regression analysis using GraphPad Prism 5.03 software. The activity of each compound was compared with control samples incubated with culture medium (M199 medium for *L. amazonensis* and Schneider’s medium for *L. infantum chagasi*) containing less than 0.05% of polysorbate 80 (Sigma-Aldrich) or amphotericin B at 5 µM (100% mortality: Sigma-Aldrich) as reference antileishmanial drug.

### 2.4 Evaluation of leishmania growth

Promastigote forms of *L. amazonensis* or *L. infantum chagasi* in the stationary growth phase were distributed in 12-well microplates at a density of 1 x 10^5^ mL^-1^ in medium (control) (M199 medium for *L. amazonensis* and Schneider’s medium for *L. infantum chagasi*) or medium plus C_10_MImMeS, C_16_MImPF_6_, C_16_MImBr, C_16_M_2_ImCl, C_16_M_4_ImCl, (C_10_)_2_MImCl, C_16_Im or C_16_PyrCl at the IC_50_ found after 48 h of incubation, and the parasite growth at 26 °C was evaluated daily by motility and cell density using a hemocytometer (28).

### 2.5 Field emission scanning electron microscopy

To evaluate parasite ultrastructural alterations by scanning electron microscopy (SEM), promastigotes of *L. amazonensis* in the stationary phase were distributed in 96-well microplates (3 x 10^6^ cells/well) and incubated at 26 °C with M199, M199 containing IS at the IC_50_ or amphotericin B at 0.4 µM (IC_50_) for 6 h. The parasites were washed with phosphate buffered saline (PBS) and fixed with 25% glutaraldehyde in 0.2 M phosphate buffer pH 7.4. After being fixed three times with the same buffer, the parasites were adhered to glass slides previously coated with 0.1% aqueous poly-L-lysine for 30 min at room temperature. Subsequently, the parasites were washed three times with 0.2 M phosphate buffer pH 7.4 and post-fixed in a solution of 2% OsO_4_ with 0.2 M phosphate buffer (1:1) for 45 min at room temperature. All samples were dehydrated in a graded series of acetone in water from 30% to 100%, critical point dried using CO_2_, mounted on metal stubs, and coated with gold for observation in a field emission scanning electron microscope (Inspect F50, FEI) (29). In addition, the IS C_16_MImMes and C_16_MImCl, tested in our previous study (13), were also analyzed.

### 2.6 Evaluation of cell membrane integrity

*L. amazonensis* promastigotes (2 x 10^5^) in the logarithmic growth phase were distributed in 96-well microplates and either untreated, treated with IS (2-fold IC_50_ for 48 h), hydrogen peroxide (H_2_O_2_) 2 mM or amphotericin B (0.4 μM) as positive control. After 48 h at 26°C, the parasites were harvested and washed twice with PBS, resuspended and incubated with 100 μL of propidium iodide (PI – Sigma–Aldrich) 50 μg/mL at 26°C for 15 min. Following, each sample was completed with 400 μL of PBS and a total of 20,000 events were acquired and analyzed on BD FACScalibur flow cytometer and CellQuest Pro software. PI fluorescence intensity was quantified as the percentage of the fluorescence compared with untreated promastigotes (30). Results were obtained from two independent experiments performed in triplicate.

### 2.7 Determination of the mitochondrial membrane potential (ΔΨm)

Mitochondrial membrane potential (ΔΨm) was quantified using the fluorescent dye rhodamine 123 (Rh 123, Sigma-Aldrich) (29). Briefly, promastigote forms of *L. amazonensis* (2 x 10^5^) in the logarithmic growth phase were incubated with M199 medium (6 h), medium plus IS (2-fold IC_50_ – 6 h), amphotericin B (0.4 μM - 6 h) or H_2_O_2_ (2 mM - 1 h) in 96-well microplates, at 26°C. These tested concentrations and time were chosen based on previous results with other IS (13). Then, cells were washed in PBS and incubated with 500 μL of Rh 123 (1 µg/mL) for 10 min at 37 °C. After being washed, they were resuspended in 0.5 mL of PBS and the analysis was performed using a BD FACSCalibur (Becton–Dickinson^®^, Rutherford, NJ, USA) flow cytometer and CellQuest^®^ Pro software (Joseph Trotter, Scripps Research Institute, La Jolla, CA, USA), using the blue argon-ion 488 nm laser with the FL1 filter channel. A total of 30,000 events were acquired in the region that corresponded to the parasites. Histograms were build using the CellQuest Pro software (Joseph Trotter, Scripps Research Institute, La Jolla, CA, USA).

### 2.8 Detection of reactive oxygen species production

ROS generation was quantified using the dye 2′,7′-dichlorofluorescein diacetate (DCF-DA, Sigma-Aldrich) and flow cytometer analysis (13, 30, 31). Briefly, promastigote forms of *L. amazonensis* (2 x 10^5^/well) in the logarithmic growth phase were incubated with M199 medium (6 h), M199 plus IS (2-fold IC_50_ - 6 h), amphotericin B (0.4 μM - 6 h) or H_2_O_2_ (2 mM – 1 h) in 96-well microplates, at 26°C. Then, cells were washed with PBS and incubated with 0.5 mL of 10 µM DCF-DA for 30 min at room temperature. After being washed with PBS, cells were resuspended in 0.5 mL PBS and analysed using a BD FACSCalibur (Becton–Dickinson, Rutherford, NJ, USA) flow cytometer and CellQuest Pro software (Joseph Trotter, Scripps Research Institute, La Jolla, CA, USA) using the blue argon-ion 488 nm laser with the FL1 filter channel. A total of 30,000 events were acquired in the region that corresponded to living parasite.

### 2.9 Intramacrophage anti-amastigote activity

The anti-amastigote assay was performed as previously described (13). Macrophages RAW 264.7 cell line (ATCC) were cultured in DMEM plus 10% fetal bovine serum (FBS), penicillin (100 U/mL) and streptomycin (100 µg/mL) until reaching 90% confluence, when were distributed into 96-well microplates at a concentration of 2 x 10^5^ cells/well in the presence of DMEM. After 12-16 h at 37°C in a humidified atmosphere of 5% CO_2_, non-adherent cells were removed, and adherent macrophages were infected with promastigotes of *L. amazonensis* (10 parasites/cell) in the stationary phase, and 4 h later were washed with PBS to remove parasites and incubated with DMEM (control), lipopolysaccharide (LPS) (10 ng/mL) plus interferon-γ (IFN-γ (1 ng/mL) as positive control, or IS, C_16_Im or C_16_PyrCl at concentrations of 0.1, 1 μM or 5 μM for 48 h. After, cells were incubated with 100 µL of 0.01% (w/v) SDS solution in serum-free M199 medium at 37°C for 20 min. Then, cells were supplemented with M199 30% FBS and cultured at 26°C for 7 days to determine the number of promastigotes recovered, once only viable amastigotes are capable to differentiate to motile promastigotes. The leishmanicidal activity of macrophages was assessed by determining the number of viable parasites (4 replicates) using a hemocytometer.

### 2.10 Macrophage viability and hemolytic assay

RAW cells were incubated or not with IS, C_16_Im or C_16_PyrCl (5 to 200 μM) for 48 h at 37 °C and *in vitro* cytotoxicity determined by MTT assay (30). Triton X-100 1% was used as positive control. The hemolytic assay was performed according to the procedure used by (13). Briefly, red blood cells solution 1% (v/v) obtained from healthy voluntary donors (n=3) (project approved by the research ethical committee from the Universidade Federal de Ciências da Saúde de Porto Alegre - CAAE 63282416.6.0000.5345) were distributed into 96-well microplate and incubated with different IS, C_16_Im or C_16_PyrCl (1 to 100 μM). The microplate was incubated at 37°C under agitation (90 rpm) for 60 min. Then, the suspension was centrifuged at 3,000 rpm for 5 min, and the absorbance of the supernatant was measured at 540 nm using a spectrophotometer EZ Read 400 from Biochrom. Triton X-100 1% and PBS were used as positive and negative control, respectively. The 50% cytotoxicity concentration (CC_50_) and 50% of red blood cell hemolysis (RBC_50_) were determined by nonlinear regression analysis. The selectivity index (SI) (CC_50_ or RBC_50_/IC_50_ against *L. amazonensis*) were determined for each compound.

### 2.11 Statistical analysis

The results are expressed as mean ± standard error of the mean (SEM) from five replicates in each three independent experiments. Kolmogorov-Smirnov test was applied to verify normality. Comparisons were performed by the One-way ANOVA test followed by Bonferroni’s post-test, Two-way ANOVA repeated measures test followed by Bonferroni’s post-test. Differences were considered statistically significant when p < 0.05. All statistical tests were performed using GraphPad Prism 6.01 software.

## 3 Results

### 3.1 Imidazolium salts

The results regarding the syntheses of IS are reported in the **Supplementary materials**, together with ^1^H NMR spectra of the synthesized compounds, and ^13^C NMR and FTIR spectra for the novel IS.

### 3.2 Leishmanicidal activity of the compounds on promastigotes of *L. amazonensis* and *L. infantum chagasi*

Six of the 24 IS tested (**Figure 1**) were able to kill promastigotes of *L. amazonensis* in a concentration-dependent manner (**Figure S38A**: Leishmanicidal activity of imidazolium salts, C_16_Im and C_16_PyrCl (0.1 to 100 μM) on promastigote forms of *L. amazonensis*, determined at 48 h incubation using MTT assay). C_10_MImMeS, C_16_MImPF_6_, C_16_MImBr, C_16_M_2_ImCl, C_16_M_4_ImCl and (C_10_)_2_MImCl at 5 µM caused a significant decrease in the viability after 48 h, resulting in 60, 37, 42, 85, 95 and 97% of mortality, respectively. The neutral imidazole C_16_Im and the pyridinium salt C_16_PyrCl at concentrations ≥ 10 µM and ≥ 5 µM caused significant parasite mortality, respectively (**Figure S38A**). The IC_50_ values of C_10_MImMeS, C_16_MImPF_6_, C_16_MImBr, C_16_M_2_ImCl, C_16_M_4_ImCl and (C_10_)_2_MImCl on promastigotes of *L. amazonensis* were calculated to be 11.6, 6.9, 6.0, 4.1, 1.8 and 1.9 µM, respectively (**Table 1**). Although C_10_MImMeS, C_16_MImPF_6_, C_16_MImBr, C_16_M_2_ImCl, C_16_M_4_ImCl and (C_10_)_2_MImCl also presented leishmanicidal activity against *L. infantum chagasi* (**Figure S38B**: Leishmanicidal activity of imidazolium salts, C_16_Im and C_16_PyrCl (0.1 to 100 μM) on promastigote forms of *L. infantum chagasi*, determined at 48 h incubation using MTT assay), the IC_50_ values were in general higher than against *L. amazonensis* (**Table 1**). In contrast, the IC_50_ values for C_16_Im and C_16_PyrCl were lower in the case of *L. infantum chagasi*. To reinforce the higher activity of IS towards *L. amazonensis* we tested the effect of IS with the best activity against *L. amazonensis* in our previous study (13) in promastigotes of *L. infantum chagasi*. The IS C_10_MImCl, C_16_MImCl and C_16_MImMeS presented lower IC_50_ values for *L. amazonensis* (2.31, 1.23 and 0.75 µM, respectively) (13) when compared to those values found for *L. infantum chagasi* (IC_50_ = 114.9 µM, 9.9 µM and 10.8 µM, respectively; data not shown). As expected, amphotericin B at 5 µM caused 100% mortality of *L. amazonensis* and *L. infantum chagasi.*

**Table 1:** Leishmanicidal activity, macrophage toxicity and hemolytic effect of compounds.

Compounds	IC_50_ ^a^ (CI 95%^b^)	IC_50_ ^c^ (CI 95%)	IC_50_ ^d^ (CI 95%)	CC_50_ ^e^ (CI 95%)	RBC_50_ ^f^ (CI 95%)	SI^g^	SI^h^	SI^i^	SI^j^
C_10_MImMeS	11.6 (10.5-12.9)	0.3 (0.2-0.4)	> 100	76.9 (63.8-92.8)	> 100	6.6	> 8.6	256	333
C_16_MImPF_6_	6.9 (6.4-7.3)	0.3 (0.2-0.3)	26.3 (20.1-34.5)	16.8 (14.1-20.1)	24.8 (22.9-26.8)	2.4	3.6	56	82.7
C_16_MImBr	6 (5.6-6.3)	0.2 (0.1-0.2)	29.7 (25.1-35.1)	16 (12.1-21.3)	22.8 (21.7-24)	2.7	3.8	80	114
C_16_M_2_ImCl	4.1 (3.9-4.3)	0.2 (0.1-0.2)	15.4 (13.2-17.9)	19.8 (18.1-21.7)	21.6 (20.8-22.5)	4.8	5.3	99	108
C_16_M_4_ImCl	1.8 (1,7-1.9)	0.2 (0.1-0.3)	14.9 (12.7-17.5)	17.8 (16.3-19.5)	15.6 (14.5-16.8)	10	8.7	89	78
(C_10_)_2_MImCl	1.9 (1.7-2)	0.2 (0.1-0.2)	16.5 (14.5-18.8)	16.4 (14.4-18.8)	20.9 (20-21.9)	8.6	11	82	105
C_16_Im	14.6 (12.6.-17.1)	0.2 (0.1-0.2)	8.3 (4.4-15.5)	54.9 (39.9-75.6)	> 100	3.8	> 6.8	275	> 500
C_16_PyrCl	4.0 (2.0-7.9)	0.2 (0.2-0.3)	2.6 (1.4-4.6)	6.3 (4.8-8.1)	21.3 (19.2-23.7)	1.6	5.3	31.5	107

Data are presented on µM.

a IC_50_: concentration of compound that causes 50% of L. amazonensis promastigotes mortality.

b CI 95%: 95% confidence interval.

c IC_50_: concentration of compound that causes 50% of L. amazonensis amastigotes mortality.

d IC_50_: concentration of compound that causes 50% of L. infantum chagasi promastigotes mortality.

e CC_50_: concentration of compound that causes 50% of macrophage mortality.

f RBC_50_: concentration of compound that causes 50% of red blood cell hemolysis.

g SI: selectivity index, calculated as ratio of CC_50_ for macrophage/IC_50_ for L. amazonensis promastigotes.

h SI: selectivity index, calculated as ratio of CC_50_ for RBC hemolysis/IC_50_ for L. amazonensis promastigotes.

i SI: selectivity index, calculated as ratio of CC_50_ for macrophage/IC_50_ for L. amazonensis amastigotes.

j SI: selectivity index, calculated as ratio of CC_50_ for RBC hemolysis/IC_50_ for L. amazonensis amastigotes.

### 3.3 Imidazolium salts, C_16_Im and C_16_PyrCl inhibit the growth of promastigotes of *L. amazonensis* and *L. infantum chagasi*

**Figure 2:**
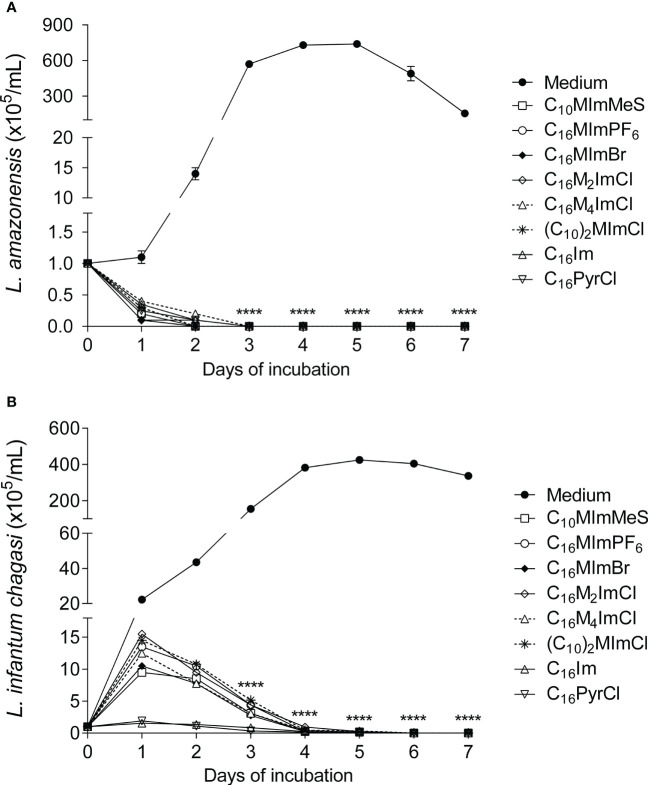
Growth kinetics of *L. amazonensis* and *L. infantum chagasi* promastigotes treated with the different compounds. *L. amazonensis* **(A)** *or L. infantum chagasi* **(B)** promastigotes in the stationary phase (1 × 10^5^ cells/mL) were incubated with medium as control (M199 to *L. amazonensis* and Schneider’s medium to *L. infantum chagasi*) or medium plus compounds at IC_50_ values (for 48 h incubation), at 26°C, and the growth was determined daily using a hemocytometer. Data are expressed as means ± SEM (n = 5) and are representative of three independent experiments. The Two-way ANOVA repeated measures test followed by the Bonferroni’s test were applied. ****p < 0.0001.

When promastigotes of *L. amazonensis* or *L. infantum chagasi* were treated with IS, C_16_Im or C_16_PyrCl at IC_50_ concentrations, a complete inhibition of the parasites growth was observed (**Figures 2A, B**).

### 3.4 Imidazolium salts increase reactive oxygen species and induce mitochondrial membrane potential (ΔΨm) alterations in *L. amazonensis*

**Figure 3:**
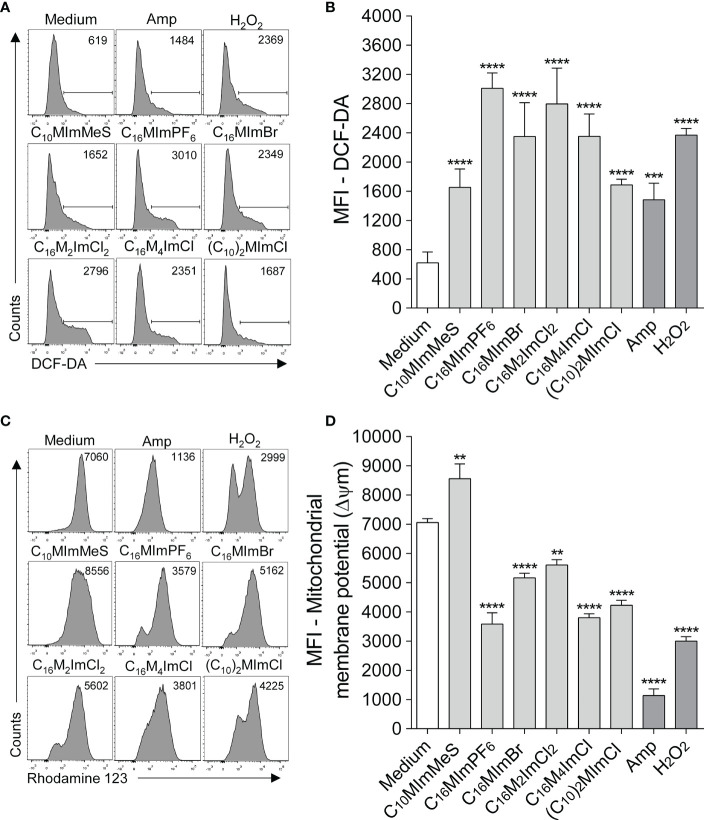
Determination of intracellular ROS **(A, B)** and mitochondrial membrane potential (ΔΨm) **(C, D)** of *L. amazonensis* promastigotes incubated with imidazolium salts. Promastigote forms in the logarithmic phase (2 x 10^5^ cells/well) were incubated or not with 2-fold IC_50_ value of IS at 26°C for 6 h. M199 was used as negative control, and amphotericin B (0.4 µM) and H_2_O_2_ (2 mM) as positive controls. Data are expressed as means ± SEM and are representative of three independent experiments. The One-way ANOVA test followed by the Bonferroni’s test were applied. **p < 0.01, ***p < 0.001 and ****p < 0.0001 when compare with the medium group.

The treatment of promastigotes of *L. amazonensis* with IS at 2-fold IC_50_ values led to a significant increase in ROS production at 6 h of incubation (**Figures 3A, B**). C_16_MImPF_6_, C_16_MImBr, C_16_M_2_ImCl, C_16_M_4_ImCl and (C_10_)_2_MImCl caused significant ΔΨm depolarization (loss of Rh 123 fluorescence), while C_10_MImMeS induced ΔΨm hyperpolarization (increase in Rh 123 fluorescence) (**Figures 3C, D**). Hydrogen peroxide at 2 mM or amphotericin B at 0.4 µM also induced significant elevation in ROS production and mitochondrial depolarization.

### 3.5 Imidazolium salts induce loss of plasma membrane integrity in *L. amazonensis*

**Figure 4:**
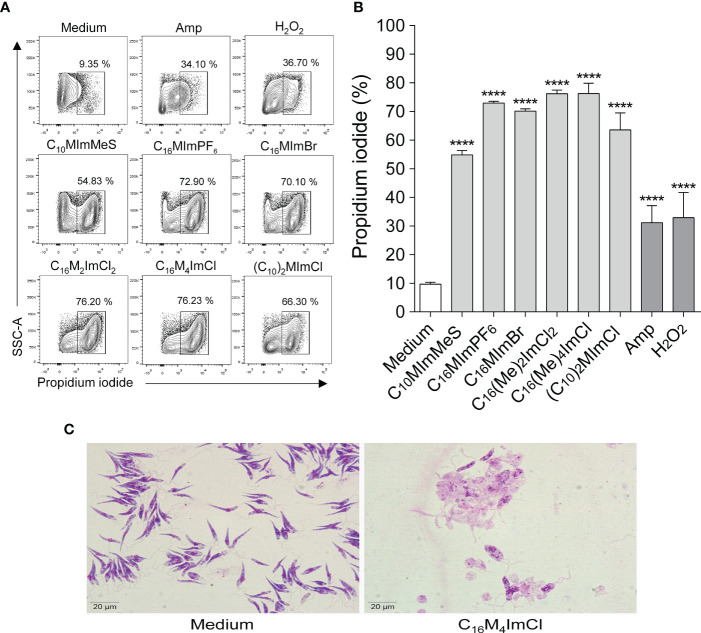
Determination of membrane integrity of *L. amazonensis* promastigotes treated with imidazolium salts **(A, B)**. Promastigote forms in the logarithmic phase (2 x 10^5^ cells/well) were incubated or not with 2-fold IC_50_ value of IS for 48 h at 26°C. M199 was used as negative control, and amphotericin B (0.4 µM) or H_2_O_2_ (2 mM) as positive controls. Data are expressed as means ± SEM (n = 5) and are representative of three independent experiments. The One-way ANOVA test followed by the Bonferroni’s test were applied. ****p < 0.0001 when compare with the medium group. **(C)** *L. amazonensis* integrity after 48 h of exposure to C_16_M_4_ImCl at 1.8 μM or M199 (panoptic stain – 1000 × magnification).

When parasites were treated with IS at 2-fold IC_50_ values for 48 h, a significant increase in propidium iodide labeling was observed for all IS tested, indicating that the IS interfere with membrane integrity causing cell death (**Figures 4A, B**). Besides, as illustrated in **Figure 4C**, incubation with C_16_M_4_ImCl at 1.8 µM (IC_50_) for 48 h led to total lysis of the parasites.

### 3.6 Imidazolium salts cause morphological alterations on *L. amazonensis* promastigotes

**Figure 5:**
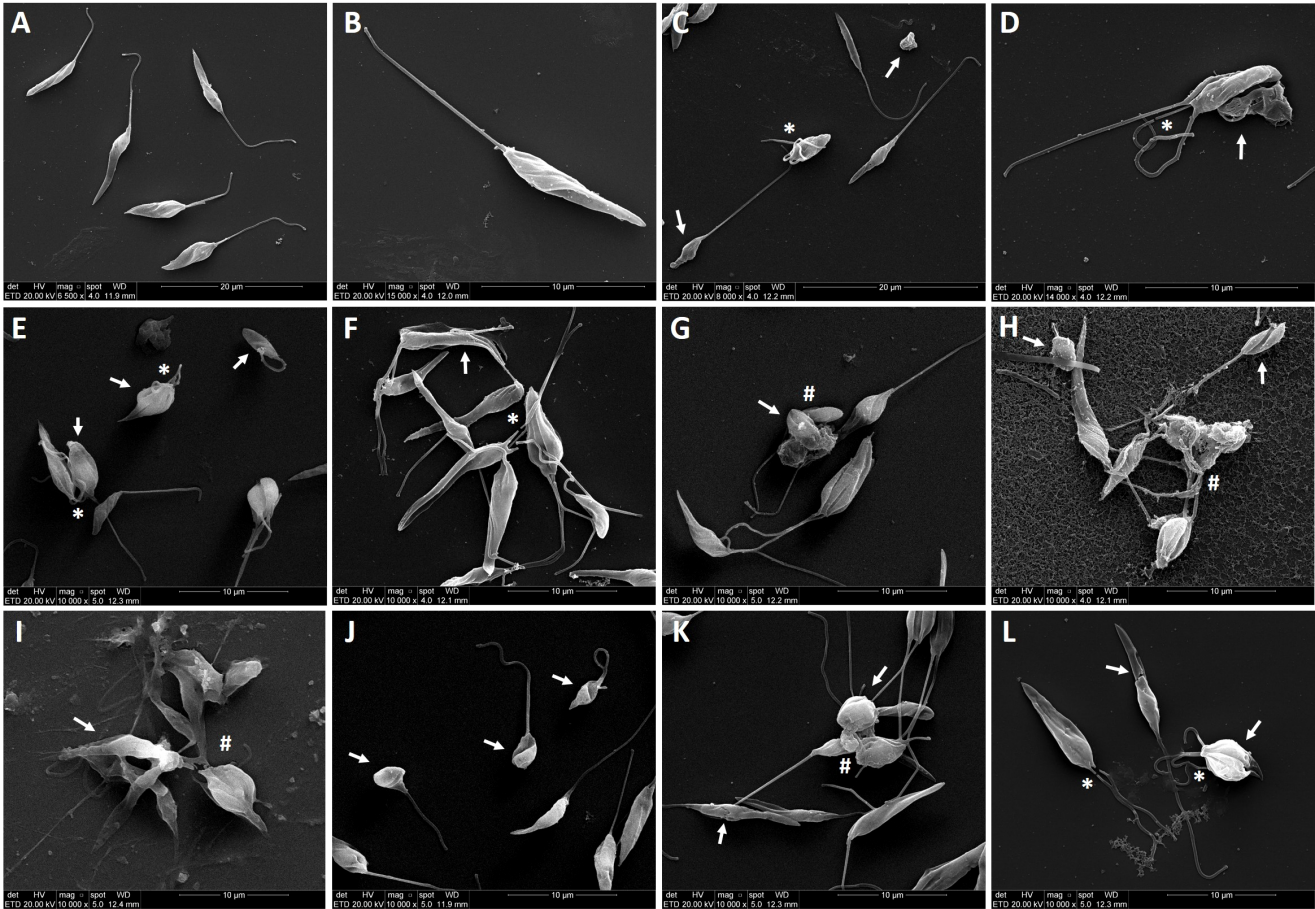
Scanning electron micrographs of *L. amazonensis* promastigotes treated or not with imidazolium salts at IC_50_ values for 6 h. Untreated parasites showing the typical elongated shape (parasite body and anterior flagella) **(A, B)**. Parasites treatment with amphotericin B at 0.4 µM **(C, D)**, C_10_MImMeS at 11.6 µM **(E)**, C_16_MImPF_6_ at 6.9 µM **(F)**, C_16_MImBr at 6 µM **(G)**, C_16_M_2_ImCl at 4.1 µM **(H)**, C_16_M_4_ImCl at 1.8 µM **(I)**, (C_10_)_2_MImCl at 1.9 µM **(J)**, C_16_MImMeS at 0.75 µM **(K)** and C_16_MImCl at 1.23 µM **(L)**. The images show alterations in promastigote body shape or disruption of plasma membrane (arrows), flagellum loss (asterisks), and aggregation of cells (hashes). Bars: **(A, C)** represent 20 µm, **(B)** and **(D–L)** represent 10 µm.

As illustrated in **Figure 5**, when promastigotes were treated with IS at IC_50_ values for 6 h, the parasites showed striking morphological alterations. While untreated parasites exhibited elongated shape, integral flagellum and membrane integrity (**Figures 5A, B**), promastigotes treated with amphotericin B at 0.4 µM (**Figures 5C, D**), as well as with C_10_MImMeS, C_16_MImPF_6_, C_16_MImBr, C_16_M_2_ImCl, C_16_M_4_ImCl or (C_10_)_2_MImCl (**Figures 5E–J**) evidenced rounded shape, membrane damage, flagellum disruption or twisted, and cell aggregation. In addition, C_16_MImMeS and C_16_MImCl that killed *L. amazonensis* in our previous study (13) also caused similar morphological alterations in promastigotes of *L. amazonensis* (**Figures 5K, L**).

### 3.7 Cytotoxicity of imidazolium salts on mammalian cells

*In vitro* cytotoxicity of imidazolium salts, C_16_Im and C_16_PyrCl (5 to 200 μM) towards macrophages, determined by MTT assay

In relation to the effects of IS on macrophages, a concentration-dependent cytotoxicity was found (**Figure S39A**: *In vitro* cytotoxicity of imidazolium salts, C_16_Im and C_16_PyrCl [5 to 200 μM] towards macrophages, determined by MTT assay). All IS at 10 µM kept more than 90% of the macrophages alive after 48 h of incubation, and C_10_MImMeS maintained this condition up to 50 µM. Besides that, C_16_M_4_ImCl, (C_10_)_2_MImCl and C_10_MImMeS at concentrations of 5.6-, 5.3- and 4.3-fold IC_50_ for *L. amazonensis* kept 90, 94 and 89% of macrophages viability, respectively. C_16_M_4_ImCl and (C_10_)_2_MImCl presented the best selectivity indexes (10 and 8.6, respectively) (**Table 1**). Moreover, human erythrocytes incubated with IS at 10 µM demonstrated hemolysis up to 8% and, as well as for macrophages, C_10_MImMeS was the less toxic IS, without significant hemolysis up to 40 µM (**Figure S39B**: *In vitro* cytotoxicity of imidazolium salts, C_16_Im and C_16_PyrCl (1 to 100 μM) towards human red blood cells, determined by hemolysis rates at 540 nm). C_16_M_4_ImCl and (C_10_)_2_MImCl also presented the best SI when considering hemolysis – 8.7 and 11, respectively (**Table 1**). The neutral imidazole C_16_Im presented lower cytotoxicity against both macrophages and erythrocytes, however it presented low SI (**Table 1**). The pyridinium salt C_16_PyrCl presented the higher toxicity and lowest SI among the tested compounds (**Table 1**).

### 3.8 Imidazolium salts reduce the intracellular survival of *L. amazonensis* on macrophages

**Figure 6:**
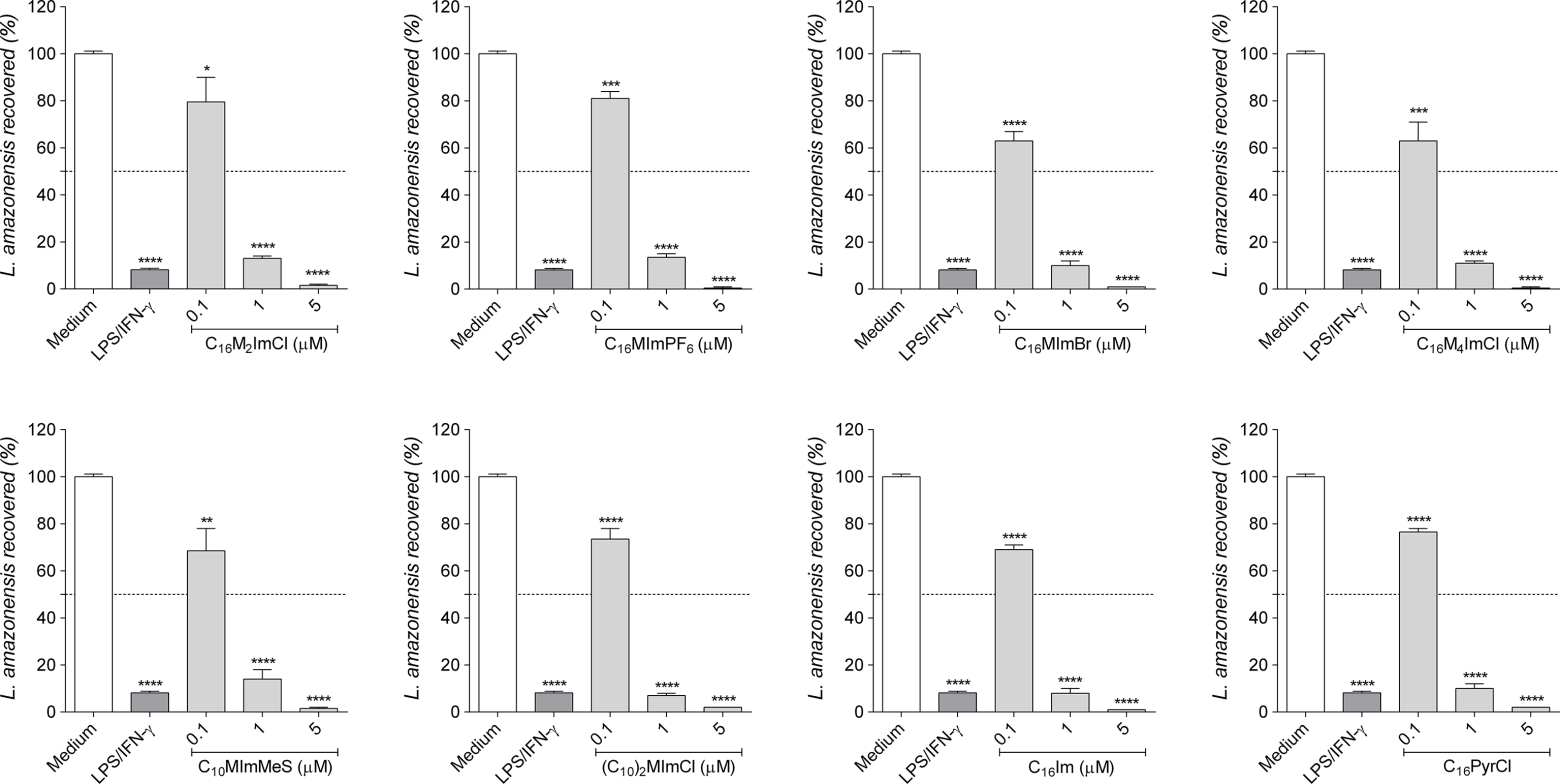
Intramacrophage anti-amastigote activity of compounds. RAW cells (2 × 10^5^ cells/well) were infected with promastigotes of *L. amazonensis* (10 parasites/cell) in the stationary phase (day 5 of culture) and incubated with DMEM (control), lipopolysaccharide (LPS; 10 ng/mL) plus interferon-γ (IFN-γ; 1 ng/mL) as positive control, or IS, C_16_Im or C_16_PyrCl at concentrations of 0.1 μM, 1 μM or 5 μM for 48 h. After, cells were lysed, supplemented with M199 30% FBS and cultured at 26°C for 7 days to determine the number of promastigotes recovered using a hemocytometer. Data are expressed as means ± SEM (n = 5). The One-way ANOVA test followed by the Bonferroni’s test were applied. *p < 0.05, ** p < 0.01, ***p < 0.001 and ****p < 0.0001 when compared with the medium group (100% parasite recovered).

As *Leishmania* can survive inside macrophages, we evaluate whether IS, C_16_Im and C_16_PyrCl stimulate the leishmanicidal activity of macrophages. Our data showed that all compounds at 1 µM were able to reduce amastigotes survival in 86% to 93% (**Figure 6**).

## 4 Discussion

C_4_MImMeS, C_10_MImMeS, C_16_MImBr, C_4_MImBF_4_, MImC_10_MImBr_2_, C_7_O_3_MImPF_6_, C_7_O_3_MImMeS, C_4_M_2_ImMeS, (C_16_)_2_ImCl, HOC_3_MImPF_6_, HO_2_CC_1_MImCl, (HO_2_CC_1_)_2_ImCl, HO_2_CC_1_MImBF_4_, HO_2_CC_3_MImCl, C_16_MImPF_6_, C_16_M_4_ImCl, C_16_M_2_ImCl, MImC_5_MImBr_2_, MImC_8_MImBr_2_, (C_16_)_2_ImMeS, (C_16_)_2_MmCl, and (C_16_)_2_MImMeS were synthesized, which along with the commercially available (C_10_)_2_MImCl, and HOC_2_MImCl constituted the group of 24 IS tested (**Figure 1**). The spectral data of C_4_MImMeS (14), C_10_MImMeS (15), C_16_MImBr (16), C_4_MImBF_4_ (14), MImC_10_MImBr_2_ (16), C_7_O_3_MImPF_6_ (15), C_7_O_3_MImMeS (15), C_4_M_2_ImMeS (17), C_16_Im (18), (C_16_)_2_ImCl (19), HOC_3_MImPF_6_ (20), HO_2_CC_1_MImCl (21), (HO_2_CC_1_)_2_ImCl (21), HO_2_CC_1_MImBF_4_ (22), HO_2_CC_3_MImCl (23), C_16_MImPF_6_ (24), C_16_M_4_ImCl (25), C_10_MeS (15), C_16_MeS (26), C_4_MeS (14), C_16_MIm (27), and C_7_O_3_MeS (15) are in agreement with those previously reported. For the new IS C_16_M_2_ImCl, MImC_5_MImBr_2_, MImC_8_MImBr_2_, (C_16_)_2_ImMeS, (C_16_)_2_MmCl, and (C_16_)_2_MImMeS, the NMR, FTIR and MS data support their structures and purity.

To the best of our knowledge, this is the first study reporting the leishmanicidal and immunomodulatory activities for C_10_MImMeS, C_16_MImPF_6_, C_16_MImBr, C_16_M_2_ImCl, C_16_M_4_ImCl and (C_10_)_2_MImCl (**Figure 1**) on two *Leishmania* species. Here, it was demonstrated that depending on the concentration, IS were able to kill infective forms of *L. amazonensis* and *L. infantum chagasi* (**Figure S38**).

In a previous study, we demonstrated that IS are able to kill both promastigote and amastigote forms of *L. amazonensis* (13). Here, we investigated a structurally complementary set of mono- (*N*-substituent = methyl; *N’*-substituent = *n-*butyl, *n-*decyl, *n*-hexadecyl, 2-hydroxyethyl, methylcarboxylic acid, propylcarboxylic acid, methoxycarbonylmethyl, triethylene glycol monomethyl ether; C_2_-substituent = H, methyl; C_4_-substituent = H, methyl; C_5_-substituent = H, methyl – *N,N’-substituents = n*-decyl, *n*-hexadecyl, methylcarboxylic acid; C_2_-substituent = H, methyl) and dicationic (*N*-substituent = methyl; alkane bridge = pentane, octane, decane) IS with different anions, as well as the neutral C_16_Im and the pyridinium salt C_16_PyrCl (**Figure 1**) against *L. amazonensis* and *L. infantum chagasi*. This provided the perspective to compare the chemical structures and extend the comprehension about activity of this class of compounds against *Leishmania*. Significant differences in leishmancidal activity of IS against *L. amazonensis* were observed (**Table 1**). (C_10_)_2_MImCl and C_16_M_4_ImCl presented the highest activity (lowest IC_50_ values): (C_10_)_2_MImCl = C_16_M_4_ImCl > C_16_M_2_ImCl > C_16_MImBr > C_16_MImPF_6_ > C_10_MImMes. Corroborating with scientific literature (10, 11, 13), the monocationic methylimidazolium salts with longer *N*-alkyl chain-lengths are closely related to higher leishmanicidal activities. In this sense, we point out that C_16_MImMeS showed the higher leishmanicidal activity (IC_50_ = 0.75 µM) (13) compared to C_10_MImMeS (IC_50_ = 11.6 µM) and C_4_MImMeS (without activity at 100 µM) tested here. On the other hand, shorter *N*-alkyl chain-lengths afford lower lipophilicity to the molecule and consequently less cytotoxic effects (32), as demonstrated by C_10_MImMeS which showed lower toxicity towards macrophages (CC_50_ = 76.9 µM) and erythrocytes (RBC_50_ > 100 µM) when compared to C_16_MImMeS (CC_50_ = 11.5 µM and RBC_50_ = 36.9 µM) (13).

The *N,N’*dialkyl substituted IS with *n-*decyl and *n-*hexadecyl groups (**Figure 1**) showed that, independent of the anions present in the *n-*hexadecyl-based IS (MeS, Cl or Br), only (C_10_)_2_MImCl presented leishmanicidal activity (IC_50_ = 1.9 μM), evidencing a higher leishmanicidal effect than C_10_MImCl – tested in the previous study of our group (IC_50_ = 2.31 µM) (13). Another study demonstrated that (C_10_)_2_MImCl also inhibits bacterial (*Escherichia coli* and *Staphylococcus aureus*) and fungal (*Candida albicans* and *Candida tropicalis*) growth *in vitro* (33), and the addition of two hydrophobic alkyl chains to the imidazolium ring also contributed greatly to improve the activity of IS against tumor-derived cell lines (34). This reaffirms the potential balance between hydrophilicity and lipophilicity, which is essential for the antimicrobial activity of IS (7).

The introduction of methyl groups on the C_2_-, C_4_- and C_5_-positions of the imidazolium ring influences the *L. amazonensis* mortality (**Table 1**). When comparing the activities of C_16_M_4_ImCl (IC_50_ = 1.8 µM) and C_16_M_2_ImCl (IC_50_ = 4.1 µM), the additional two methyl groups reduced 2.3-fold the IC_50_ value for *L. amazonensis* mortality. However, C_16_MImCl presented the lowest IC_50_ value among these three compounds (IC_50_ = 1.23 µM) (13). This H-methyl substituent alteration in the imidazolium ring does not seem to interfere to the same extend on *L. infantum chagasi* mortality, according to the IS C_16_M_4_ImCl (IC_50_ = 14.9 µM) and C_16_M_2_ImCl (IC_50_ = 15.4 µM).

From results of this study and our previous work (13), it is possible to compare the effect of different IS anions on *L. amazonensis* mortality. The IS C_16_MImMeS (IC_50_ = 0.75 µM), C_16_MImCl (IC_50_ = 1.23 µM) and C_16_MImNTf_2_ (IC_50_ = 1.64 µM) demonstrated higher capacities to kill *L. amazonensis* than C_16_MImBr (IC_50_ = 6.0 µM) and C_16_MImPF_6_ (IC_50_ = 6.9 µM), reaffirming the hypothesis that the IS anion also has an important but secondary effect on activity against *Leishmania* (13). For the C_16_MIm-based IS, the established order of anion-dependent inhibition was MeS > Cl > NTf_2_ > Br > PF_6_. As observed for *L. amazonensis* mortality with C_16_MImBr and C_16_MImPF_6_, Br and PF_6_ anions did not trigger differences in *L. infantum chagasi* mortality – C_16_MImPF_6_ (IC_50_ = 26.3 µM) and C_16_MImBr (IC_50_ = 29.7 µM). A decrease in anion volume has been related to an increasing fungal mortality, evidencing the IS with MeS and chloride anions as the most promising antifungals (9). This was also observed for anti-leishmanial activity of the IS.

Considering the highest leishmanial activity achieved with C_16_MImMeS, we tested the effect of its neutral imidazole equivalent C_16_Im on promastigote forms of *Leishmania*. This study showed that C_16_Im was not so effective to kill *L. amazonensis* as C_16_MImMeS. Furthermore, this compound presented lower toxicity than all other tested compounds, except C_10_MImMeS (**Table 1**). In contrast, C_16_Im showed a higher activity than the IS tested in this work against *L. infantum chagasi*.

Compounds containing cationic pyridinium heterocycles have concerned great awareness as prospective chemotherapeutic agents, such as anticancer, analgesic, antimicrobial and antiviral (35). Based on this, the effect of C_16_PyrCl on *Leishmania* mortality was tested, and this compound showed being effective against *L. amazonensis* (IC_50_ = 4.0 µM) (**Figure S38A** and **Table 1**), but being more toxic toward macrophages than IS (**Figure S39A** and **Table 1**). In our previous study we tested the structurally related IS C_16_MImCl, which showed higher leishmanicidal activity and lower macrophage and red blood cell cytotoxicity than C_16_PyrCl (13). C_16_PyrCl also demonstrated high capacity to kill *L. infantum chagasi* (IC_50_ = 2.6 µM) (**Figure S38B**). Although this compound is commonly used as antiseptic (mouthwash), its high toxicity against macrophages turns it into a weak candidate for drug development.

To elucidate possible mechanisms of action of IS on *Leishmania* parasites, membrane and mitochondrial dysfunctions, ROS generation and morphological changes in response to IS were investigated. In *Leishmania*, mitochondria are a drug target (36), since this unique organelle is the main site of ROS production, and excessive ROS may lead to oxidative stress, which may be one of the major contributors to cell death by IS (37, 38). In addition, the maintenance of an appropriate mitochondrial membrane potential (ΔΨm) is essential for *Leishmania* survival (36, 37). Here, we demonstrate that IS with leishmanicidal activity caused an increase in ROS production (**Figures 4A, B**) and ΔΨm alterations (**Figures 34C, D**) in *L. amazonensis.* C_10_MImMeS triggered mitochondrial membrane hyperpolarization, while C_16_MImPF_6_, C_16_MImBr, C_16_M_2_ImCl, C_16_M_4_ImCl and (C_10_)_2_MImCl caused depolarization of mitochondrial membrane. Both hyperpolarization and depolarization can result in promastigotes death (29), and depolarization can be preceded by a transient hyperpolarization as the last attempt by cells to avoid death (39). Moreover, all six IS presenting leishmanicidal activity caused plasma membrane alterations (**Figures 4A, B**), indicating cell death involving necrosis (40).

In this regard, the scanning electron microscopy analyses revealed that the treatment with IS induced swelling and overall rounding, cell aggregation, membrane rupture, besides lower parasite length and flagellum damage (**Figure 5**), corroborating with evidences of the action of IS on *L. amazonensis* (13). In addition to the biochemical alterations, IS inhibited the *in vitro* growth of promastigotes forms of *L. amazonensis* (**Figure 2A**) and *L. infantum chagasi* (**Figure 2B**). This effect could be related to the ergosterol availability. Similarly to fungi, ergosterol in *Leishmania* is essential for the organization of membrane domains, cell growth and survival. In a previous study it was demonstrated that C_16_MImCl reduced the total ergosterol content of clinical isolates of yeasts, suggesting that IS inhibit the ergosterol biosynthesis (8). Although C_16_MImCl and other IS presented potent leishmanicidal activity, the activity of enzyme involved in ergosterol synthesis (lanosterol 14α-demethylase) or ergosterol content were not evaluated in this study.

IS, C_16_Im and C_16_PyrCl at 1 µM reduced the amastigote survival of *L. amazonensis* in about 90% (**Figure 6**), with an IC_50_ between 0.2 to 0.3 and higher SI (31.5 to 275) than those calculated for promastigotes, indicating a higher selectivity for amastigotes. These data are in agreement with our previous data showing the better selectivity of IS for amastigotes (13).

For the first time, the IS C_10_MImMeS, C_16_MImPF_6_, C_16_MImBr, C_16_M_2_ImCl, C_16_M_4_ImCl, and (C_10_)_2_MImCl have been identified as potent agents against *L. amazonensis*. These compounds induced ROS generation, membrane, and mitochondrial dysfunction in parasites. The results reaffirm the hypothesis that IS with longer *N*-alkyl chain-lengths are related with higher biological activities, and on a secondary basis IS with MeS and chloride anions are the most promising antileishmanial agents. As a limitation of this study, we mentioned that we did not test the effects of these IS on antimony-resistant *Leishmania* spp. lines, or their *in vivo* effects.

## 5 Conclusions

The IS C_10_MImMeS, C_16_MImPF_6_, C_16_MImBr, C_16_M_2_ImCl, C_16_M_4_ImCl and (C_10_)_2_MImCl, and the compounds C_16_Im and C_16_PyrCl killed and inhibited the growth of promastigote forms of *L. amazonensis* and *L. infantum chagasi* in a concentration-dependent manner. Moreover, induced ROS production, mitochondrial dysfunction, membrane disruption and morphological alterations in infective forms of *L. amazonensis* and were able to kill intracellular amastigote forms in a very low concentration, being worthy of further *in vivo* studies as potential antileishmanial drugs.

## Data availability statement

The original contributions presented in the study are included in the article/**Supplementary Material**. Further inquiries can be directed to the corresponding authors.

## Author contributions

Conceptualization PR, HS, FB. Methodology: FB, TF, MS, PF, VS, DP, JK, ES. Software: TF, FB, LJ. Validation: FB, TF. Formal analysis: FB, LJ. Investigation: FB, ED, TF. Resources: PR, HS, MM. Data curation: FB, PR. Writing—original draft preparation: KN-L, FB, PR, TF. Writing—review and editing: MM. PR, HS. Visualization: AP, LJ, MM, ED. Supervision: PR, HS, MM. Project administration: PR, HS. Funding acquisition: PR, HS, MM. All authors contributed to the article and approved the submitted version.

## References

[1] OPAS. Manual de procedimientos para vigilancia y control de las leishmaniasis en las américas. (2019). doi: 10.37774/9789275320631.

[2] WHO. Leishmaniasis in high-burden countries: An epidemiological update based on data reported in 2014. Wkly Epidemiol Rec (2016) 91:287–96. 27263128

[3] Silveira FT Lainson R Corbett CEP. Clinical and immunopathological spectrum of american cutaneous leishmaniasis with special reference to the disease in Amazonian Brazil - a review. Mem Inst Oswaldo Cruz (2004) 99:239–51. doi: 10.1590/S0074-02762004000300001 15273794

[4] Burza S Croft SL Boelaert M. Leishmaniasis. Lancet (2018) 392:951–70. doi: 10.1016/S0140-6736(18)31204-2 30126638

[5] Tiuman TS Santos AO Ueda-Nakamura T Filho BPD Nakamura CV. Recent advances in leishmaniasis treatment. Int J Infect Dis (2011) 15:525–32. doi: 10.1016/j.ijid.2011.03.021 21605997

[6] Emami S Tavangar P Keighobadi M. An overview of azoles targeting sterol 14α-demethylase for antileishmanial therapy. Eur J Med Chem (2017) 135:241–59. doi: 10.1016/j.ejmech.2017.04.044 28456033

[7] Pendleton JN Gilmore BF. The antimicrobial potential of ionic liquids: A source of chemical diversity for infection and biofilm control. Int J Antimicrob Agents (2015) 46:131–9. doi: 10.1016/j.ijantimicag.2015.02.016 25907139

[8] Schrekker HS Donato RK Fuentefria AM Bergamo V Oliveira LF Machado MM. Imidazolium salts as antifungal agents: Activity against emerging yeast pathogens, without human leukocyte toxicity. Medchemcomm (2013) 4:1–5. doi: 10.1039/c3md00222e

[9] Dalla Lana DF Donato RK Bündchen C Guez CM Bergamo VZ de Oliveira LFS. Imidazolium salts with antifungal potential against multidrug-resistant dermatophytes. J Appl Microbiol (2015) 119:377–88. doi: 10.1111/jam.12862 26043668

[10] Goellner E Schmitt AT Couto JL Muller ND Pilz Junior HL Schrekker HS. Larvicidal and residual activity of imidazolium salts against aedes aegypti (Diptera: Culicidae). Pest Manag Sci (2018) 74:1013–9. doi: 10.1002/ps.4803 29193680

[11] Riduan SN Zhang Y. Imidazolium salts and their polymeric materials for biological applications. Chem Soc Rev (2013) 42:9055–70. doi: 10.1039/c3cs60169b 23979404

[12] Rakers L Glorius F. Flexible design of ionic liquids for membrane interactions. Biophys Rev (2018) 10:747–50. doi: 10.1007/s12551-018-0412-9 PMC5988627 29549585

[13] Martins RC Dorneles GP Teixeira VON Antonello AM Couto JL Rodrigues Júnior LC. Imidazolium salts as innovative agents against leishmania amazonensis. Int Immunopharmacol (2018) 63:101–9. doi: 10.1016/j.intimp.2018.07.038 30077823

[14] Cassol CC Ebeling G Ferrera B Dupont J. A simple and practical method for the preparation and purity determination of halide-free imidazolium ionic liquids. Adv Synth Catal (2006) 348:243–8. doi: 10.1002/adsc.200505295

[15] Schrekker HS Silva DO Gelesky MA Stracke MP Schrekker CML Gonçalves RS. Preparation, cation-anion interactions and physicochemical properties of ether-functionalized imidazolium ionic liquids. J Braz Chem Soc (2008) 19:426–33. doi: 10.1590/S0103-50532008000300009

[16] Villetti MA Ziembowicz FI Bender CR Frizzo CP Martins MAP De Souza TD. Thermodynamic insights into the binding of mono- and dicationic imidazolium surfactant ionic liquids with methylcellulose in the diluted regime. J Phys Chem B (2017) 121:8385–98. doi: 10.1021/acs.jpcb.7b03525 28787160

[17] Scott M Deuss PJ De Vries JG Prechtl MHG Barta K. New insights into the catalytic cleavage of the lignin β-O-4 linkage in multifunctional ionic liquid media. Catal Sci Technol (2016) 6:1882–91. doi: 10.1039/c5cy01554e

[18] Chakraborty A Debnath S Ghosh T Maiti DK Majumdar S. An efficient strategy for n-alkylation of benzimidazoles/imidazoles in SDS-aqueous basic medium and n-alkylation induced ring opening of benzimidazoles. Tetrahedron (2018) 74:5932–41. doi: 10.1016/j.tet.2018.08.029

[19] Rohini R Lee CK Lu JT Lin IJB. Symmetrical 1, 3-dialkylimidazolium based ionic liquid crystals. J Chin Chem Soc (2013) 60:745–54. doi: 10.1002/jccs.201200598

[20] Zhang S Qi X Ma X Lu L Deng Y. Hydroxyl ionic liquids: The differentiating effect of hydroxyl on polarity due to ionic hydrogen bonds between hydroxyl and anions. J Phys Chem B (2010) 114:3912–20. doi: 10.1021/jp911430t 20199047

[21] Huang Z Wang Y Zhang N Zhang L Darensbourg DJ. One-pot synthesis of ion-containing CO2-based polycarbonates using protic ionic liquids as chain transfer agents. Macromolecules (2018) 51:9122–30. doi: 10.1021/acs.macromol.8b01834

[22] Miao CX He LN Wang JQ Wang JL. TEMPO and carboxylic acid functionalized imidazolium salts/sodium nitrite: An efficient, reusable, transition metal-free catalytic system for aerobic oxidation of alcohols. Adv Synth Catal (2009) 351:2209–16. doi: 10.1002/adsc.200900285

[23] Xu T Waehler T Vecchietti J Bonivardi A Bauer T Schwegler J. Gluing ionic liquids to oxide surfaces: Chemical anchoring of functionalized ionic liquids by vapor deposition onto Cobalt(II) oxide. Angew Chem Int Ed (2017) 56:9072–6. doi: 10.1002/anie.201704107 28600894

[24] Xu F Matsumoto K Hagiwara R. Effects of alkyl chain length and anion size on thermal and structural properties for 1-alkyl-3-methylimidazolium hexafluorocomplex salts (C xMImAF 6, x = 14, 16 and 18; a = p, as, Sb, Nb and Ta). Dalton Trans (2012) 41:3494–502. doi: 10.1039/c2dt11693f 22289812

[25] Neves YF Eloi ACL de Freitas HMM Soares EGO Rivillo D Demétrio da Silva V. Imidazolium salts as alternative compounds to control diseases caused by plant pathogenic bacteria. J Appl Microbiol (2020) 128:1236–47. doi: 10.1111/jam.14575 31922640

[26] Toppino A Bova ME Crich SG Alberti D Diana E Barge A. A carborane-derivative “click” reaction under heterogeneous conditions for the synthesis of a promising lipophilic MRI/GdBNCT agent. Chem Eur J (2013) 19:721–8. doi: 10.1002/chem.201201634 23154917

[27] Langat J Bellayer S Hudrlik P Hudrlik A Maupin PH Gilman JW. Synthesis of imidazolium salts and their application in epoxy montmorillonite nanocomposites. Polymer (Guildf) (2006) 47:6698–709. doi: 10.1016/j.polymer.2006.06.067

[28] Antonello AM Sartori T Folmer Correa AP Brandelli A Heermann R Rodrigues Júnior LC. Entomopathogenic bacteria photorhabdus luminescens as drug source against leishmania amazonensis. Parasitology (2018) 145:1065–74. doi: 10.1017/S0031182017002001 29157317

[29] Dagnino APA Mesquita CS Dorneles GP Teixeira VDON De Barros FMC Vidal Ccana-Ccapatinta G. Phloroglucinol derivatives from hypericum species trigger mitochondrial dysfunction in leishmania amazonensis. Parasitology (2018) 145:1199–209. doi: 10.1017/S0031182018000203 29482667

[30] Cezarotto CS Dorneles A Baldissera FG da Silva MB Markoski MM Rodrigues-Júnior LC. Leishmanicidal and antichemotactic activities of icetexanes from salvia uliginosa benth. Phytomedicine (2018), 58. doi: 10.1016/j.phymed.2018.11.009 31005722

[31] Wu D Yotnda P. Production and detection of reactive oxygen species (ROS) in cancers. J Vis Exp (2011) 57:2–5. doi: 10.3791/3357 PMC3308605 22127014

[32] Garcia MT Gathergood N Scammells PJ. Biodegradable ionic liquids part II. effect of the anion and toxicology. Green Chem (2005) 7:9–14. doi: 10.1039/b411922c

[33] Raucci MG Fasolino I Pastore SG Soriente A Brentano L Dessuy M. Antimicrobial imidazolium ionic liquids for the development of minimal invasive calcium phosphate-based bio-nanocomposites. ACS Appl Mater Interfaces (2018) 10:42766–76. doi: 10.1021/acsami.8b12696 30456941

[34] Wang D Galla HJ Drücker P. Membrane interactions of ionic liquids and imidazolium salts. Biophys Rev (2018) 10:735–46. doi: 10.1007/s12551-017-0388-x PMC5988617 29302915

[35] Kumar S Sharma PK Dudhe R Kumar N. Pyridine: Potential for biological activities. J Chron Drug Delivery (2011) 2:71–8.

[36] Fidalgo LM Gille L. Mitochondria and trypanosomatids: Targets and drugs. Pharm Res (2011) 28:2758–70. doi: 10.1007/s11095-011-0586-3 21935742

[37] Granato JT Santos JA dos, Calixto SL Prado da Silva N da Silva Martins J da Silva AD. Novel steroid derivatives: Synthesis, antileishmanial activity, mechanism of action, and in silico physicochemical and pharmacokinetics studies. BioMed Pharmacother (2018) 106:1082–90. doi: 10.1016/j.biopha.2018.07.056 30119174

[38] Martins TVF Zeraik AE Alves NO de Oliveira LL Mendes TA de O. Lipophosphoglycan 3 from leishmania infantum chagasi binds heparin with micromolar affinity. Bioinform Biol Insights (2018) 12. doi: 10.1177/1177932218763363 PMC5858678 29568220

[39] Jiménez-Ruiz A Alzate JF MacLeod ET Lüder CGK Fasel N Hurd H. Apoptotic markers in protozoan parasites. Parasit Vectors (2010) 3:104. doi: 10.1186/1756-3305-3-104 21062457 PMC2993696

[40] Stroppa PHF Antinarelli LMR Carmo AML Gameiro J Coimbra ES da Silva AD. Effect of 1,2,3-triazole salts, non-classical bioisosteres of miltefosine, on leishmania amazonensis. Bioorg Med Chem (2017) 25:3034–45. doi: 10.1016/j.bmc.2017.03.051 28433512

