# Supplementary material for: Imidazolium salts as an alternative for anti-Leishmania drugs: Oxidative and immunomodulatory activities
Source: Front Immunol. 2023 Jan 17;13:1096312. doi: 10.3389/fimmu.2022.1096312 (PMC9886892; doi:10.3389/fimmu.2022.1096312)
Supplement: Supplementary file 1 [file DataSheet_1.docx]

Supplementary Material

# Supplementary Data

## Imidazolium salts and reference compounds

NMR spectra were recorded on a Bruker (400 MHz) equipment at ambient temperature. The chemical shifts are given in parts per million (ppm) and referenced to the residual solvent signal (CDCl_3_ = 7.26 (^1^H), 77.16 (^13^C); D_2_O = 4.79 (^1^H); acetone-d6 = 2.05 (^1^H); DMSO-d6 = 2.50 (^1^H)). For ^13^C NMR spectra in D_2_O, DMSO (39.39) was used as reference. Attenuated total reflection Fourier transform infrared spectroscopy (ATR-FTIR), in the mid infrared range (4000–500 cm^-1^), was performed on an ALPHA-P compact Bruker FTIR spectrometer. High-resolution mass spectrometry spectra were recorded on an electrospray ionization (ESI) Q-Tof Micro^TM^ equipment (Micromass, Manchester, UK) in the positive mode.

**C_16_M_2_ImCl**: The mixture of *n*-hexadecyl chloride (21 mmol, 1.05 equiv.), 1,2-dimethylimidazole (20 mmol, 1.00 equiv.) and acetonitrile (3 mL) was stirred and heated at 78 °C for 42 h. Next, the crude product was washed 5X with a 1:1 mixture of acetonitrile/diethyl ether. Finally, the solvent was removed under reduced pressure. **C_16_M_2_ImCl** was isolated in 84% yield as a white solid, and the following characterization data were obtained: ^1^H NMR (400 MHz, CDCl_3_) δ 7.80 (d, *J* = 2.0 Hz, 1H), 7.44 (d, *J* = 2.0 Hz, 1H), 4.17 (t, *J* = 7.5 Hz, 2H), 4.03 (s, 3H), 2.78 (s, 3H), 1.84 – 1.73 (m, 2H), 1.34 – 1.18 (m, 26H), 0.85 (t, *J* = 6.8 Hz, 3H). ^13^C NMR (101 MHz, CDCl_3_) δ 143.56, 123.22, 120.97, 77.40, 77.09, 48.80, 35.88, 31.85, 29.84, 29.80 – 29.19 (7C), 29.04, 26.35, 22.62, 14.06, 10.39. MS-ESI(+): C_21_H_41_N_2_^+^ - calculated: 321.3264, obtained: 321.0663.

**MImC_5_MImBr_2_**: The mixture of 1,5-dibromopentane (10.8 mmol, 1.00 equiv.) and 1-methylimidazole (21.6 mmol, 2.00 equiv.) was stirred and heated at 60 °C for 72 h. After cooling to room temperature, the crude product was washed with cold acetone and dried under vacuum and sonication at 45 °C. **MImC_5_MImBr_2_** was isolated in 95% yield as a white solid, and the following characterization data were obtained. ^1^H NMR (400 MHz, D_2_O) δ 8.70 (s, 2H), 7.45 (s, 2H), 7.41 (s, 2H), 4.18 (t, *J* = 7.1 Hz, 4H), 3.87 (s, 6H), 1.90 (m, 4H), 1.31 (m, 2H). ^13^C NMR (101 MHz, D_2_O) δ 135.82 (2C), 123.50 (2C), 122.08 (2C), 49.10 (2C), 35.63 (2C), 28.66 (2C), 22.12 (1C). MS-ESI(+): C_13_H_22_BrN_4_^+^ - calculated: 313.1022, obtained: 313.1389.

**MImC_8_MImBr_2_**: The mixture of 1,8-dibromooctane (10.6 mmol, 1.00 equiv.) and 1-methylimidazole (21.2 mmol, 2.00 equiv.) was stirred and heated at 60 °C for 72 h. After cooling to room temperature, the crude product was washed with cold acetone and dried under vacuum and sonication at 45 °C. **MImC_8_MImBr_2_** was isolated in 97% yield as a white solid, and the following characterization data were obtained. ^1^H NMR (400 MHz, D_2_O) δ 7.44 (2H), 7.40 (2H), 4.16 (t, *J* = 7.1 Hz, 4H), 3.86 (s, 6H), 1.84 (m, 4H), 1.32 – 1.23 (m, 8H). ^13^C NMR (101 MHz, D_2_O) δ 141.26 (2C), 123.35 (2C), 122.06 (2C), 49.43 (2C), 35.56 (2C), 29.09 (2C), 27.79 (2C), 25.15 (2C).

**(C_16_)_2_ImMeS**: The mixture of **C_16_MeS** (3.42 mmol, 1.00 equiv.) and **C_16_Im** (3.42 mmol, 1.00 equiv.) was stirred and heated at 90 °C for 24 h. After cooling to room temperature, the crude product was recrystallized from hot ethyl acetate to give white crystals, which were filtered off and dried, under vacuum. **(C_16_)_2_ImMeS** was isolated in 81% yield as white crystals, and the following characterization data were obtained. ^1^H NMR (400 MHz, CDCl_3_) δ 10.08 (m, 1H), 7.24 (d, *J* = 1.6 Hz, 2H), 4.29 (t, *J* = 7.4 Hz, 4H), 2.79 (s, 3H), 1.87 (m, 4H), 1.31-1.23 (m, 52H), 0.86 (t, *J* = 6.9 Hz, 6H). ^13^C NMR (101 MHz, CDCl_3_) δ 138.17, 121.37 (2C), 50.06 (2C), 39.55, 31.90 (2C), 30.22 (2C), 29.83 – 29.24 (m, 18C), 28.99 (2C), 26.23 (2C), 22.67 (2C), 14.11 (2C). MS-ESI(+): C_35_H_69_N_2_^+^ - calculated: 517.5455, obtained: 517.6363.

**(C_16_)_2_MImCl**: The mixture of *n*-hexadecyl chloride (3.65 mmol, 1.11 equiv.) and **C_16_MIm** (3.3 mmol, 1.00 equiv.) was stirred and heated at 120 °C for 43 h. After cooling to room temperature, the crude product was dissolved in dichloromethane and precipitated by addition of diethyl ether. The product was filtered off and dried under vacuum. **(C_16_)_2_MImCl** was isolated in 88% yield as a white solid, and the following characterization data were obtained: ^1^H NMR (400 MHz, CDCl_3_) δ 7.57 (s, 2H), 4.26 – 4.22 (m, 4H), 2.77 (s, 3H), 1.89 – 1.70 (m, 4H), 1.35 – 1.15 (m, 52H), 0.85 (t, *J* = 6.8 Hz, 6H). ^13^C NMR (101 MHz, CDCl_3_) δ 142.95 (2C), 121.62 (2C), 77.34 (2C), 77.02 (2C), 48.92 (2C), 31.89 (2C), 29.93 – 29.23 (14C), 29.07 (2C), 26.38 (2C), 22.65 (2C), 14.09 (2C), 10.52 (2C).

**(C_16_)_2_MImMeS**: The mixture of **C_16_MeS** (3.26 mmol, 1.00 equiv.) and **C_16_MIm** (3.26 mmol, 1.00 equiv.) was stirred and heated at 150 °C for 24 h. After cooling to room temperature, the crude product was recrystallized from hot ethyl acetate to give white crystals, which were filtered off and dried, under vacuum. **(C_16_)_2_MImMeS** was isolated as white crystals, an the following characterization data were obtained. ^1^H NMR (400 MHz, CDCl_3_) δ 7.35 (s, 2H), 4.17 (t, *J* = 7.5 Hz, 4H), 2.72 (s, 3H), 2.68 (s, 3H), 1.81 (m, *J* = 7.2 Hz, 4H), 1.32-1.24 (m, 52H), 0.87 (t, *J* = 7.0 Hz, 3H). ^13^C NMR (101 MHz, CDCl_3_) δ 143.31, 121.25 (2C), 48.84 (2C), 39.39, 31.91, 29.83 – 29.25 (20C), 29.04 (2C), 26.41 (2C), 22.68 (2C), 14.11 (2C), 10.28 (2C). MS-ESI(+): C_36_H_71_N_2_^+^ - calculated: 531.5612, obtained: 531.6368.

# Supplementary Figures and Tables

## Supplementary Figures


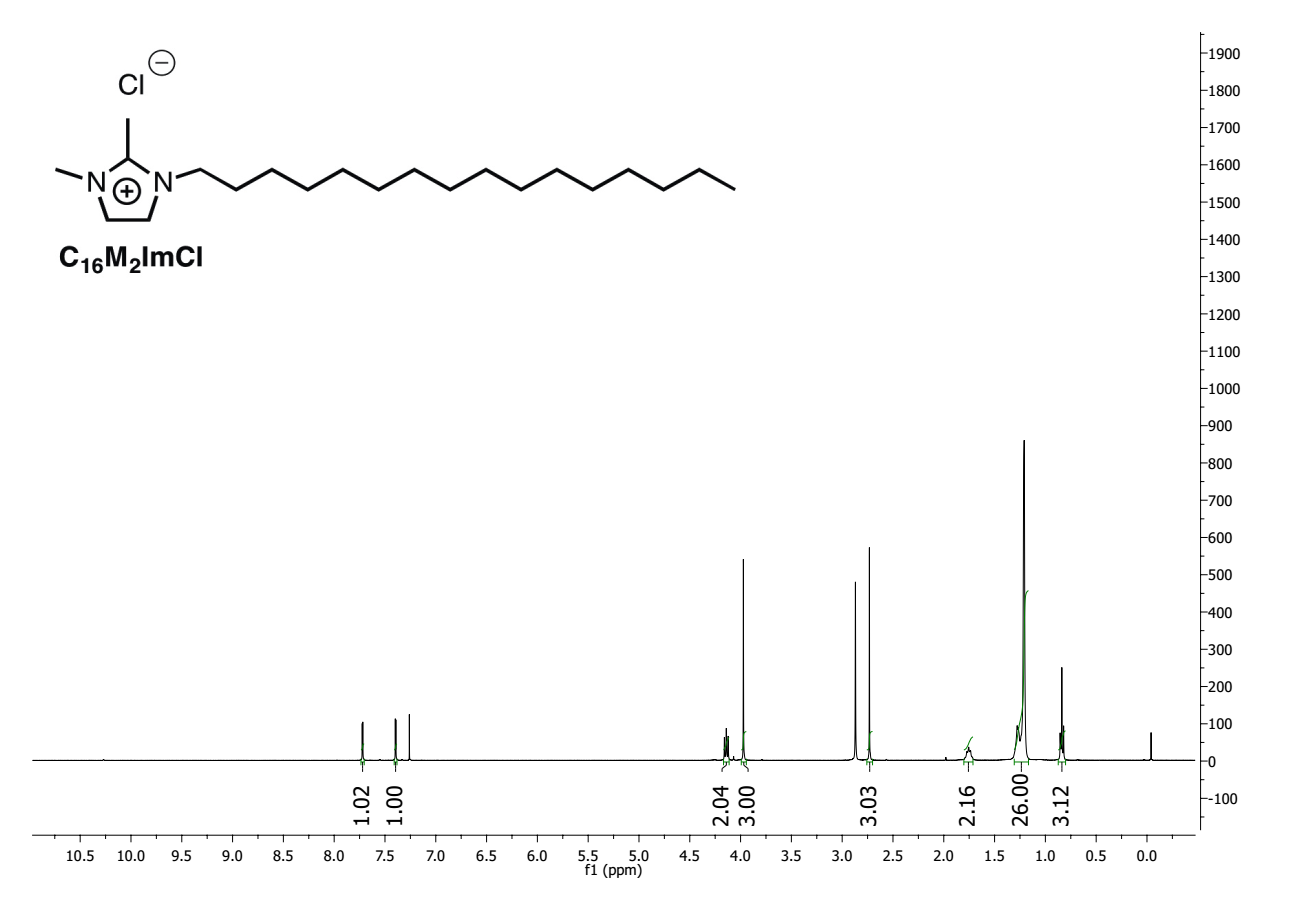


**Figure S1.** ^1^H NMR spectrum of **C_16_M_2_ImCl** (400 MHz, CDCl_3_).


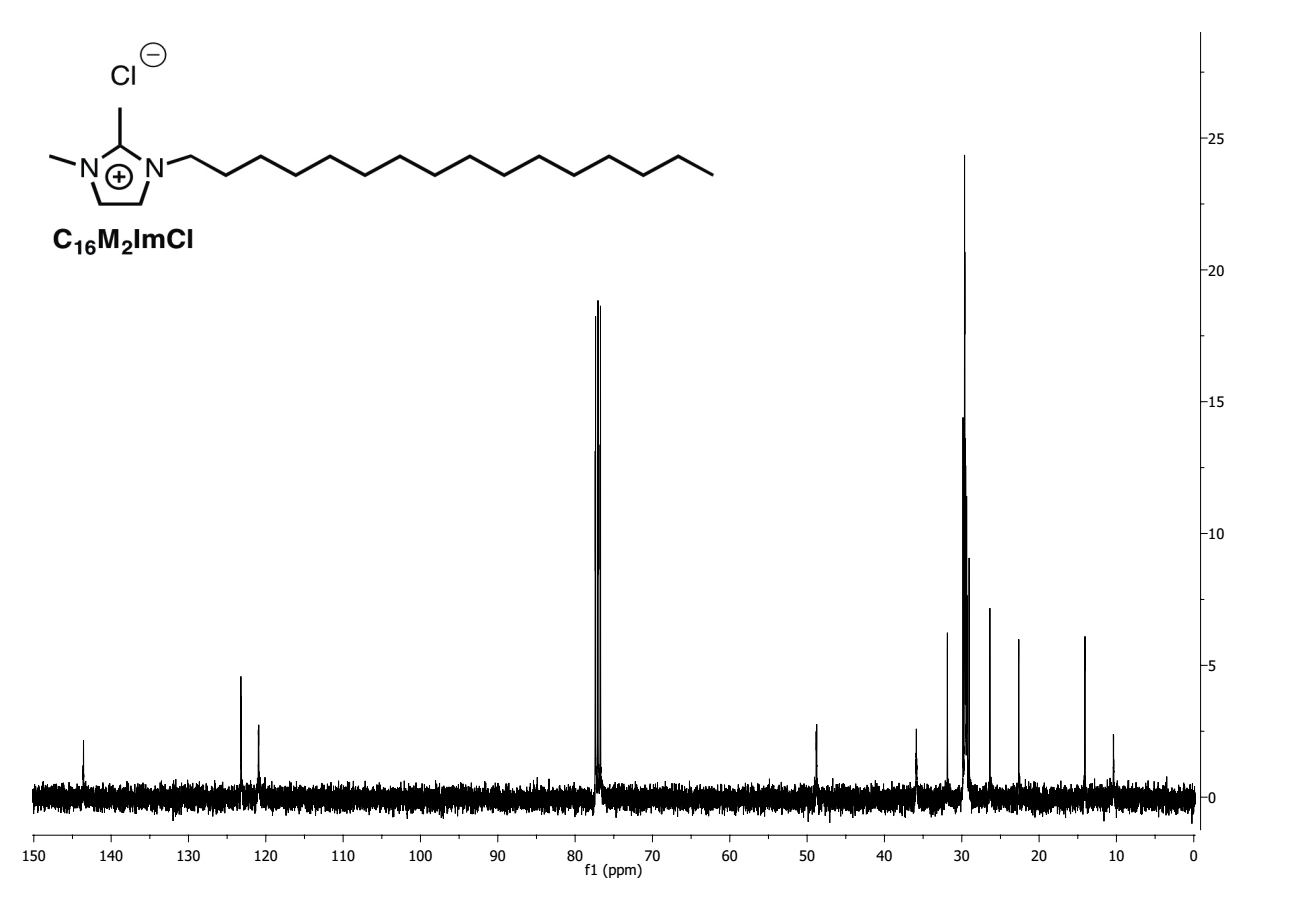


**Figure S2.** ^13^C NMR spectrum of **C_16_M_2_ImCl** (101 MHz, CDCl_3_).


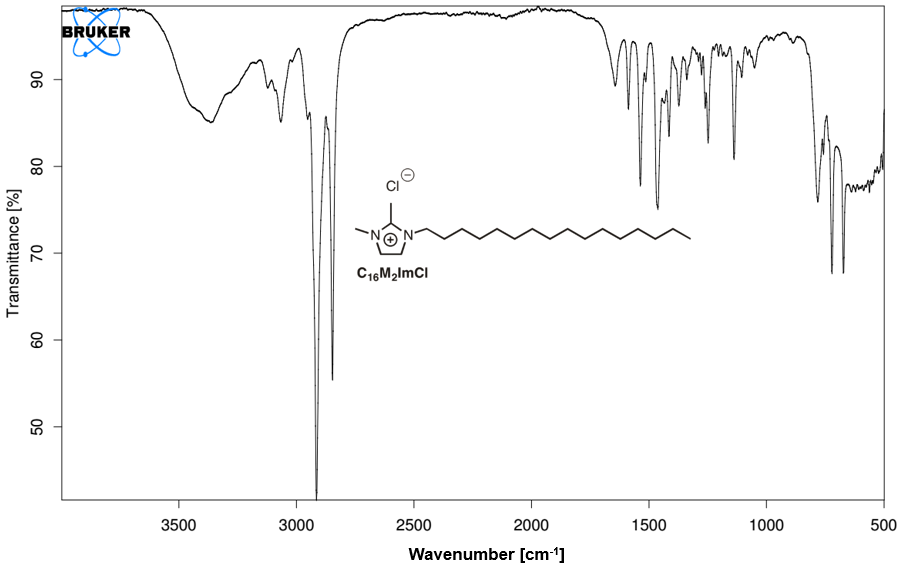


**Figure S3.** FTIR spectrum of **C_16_M_2_ImCl**.


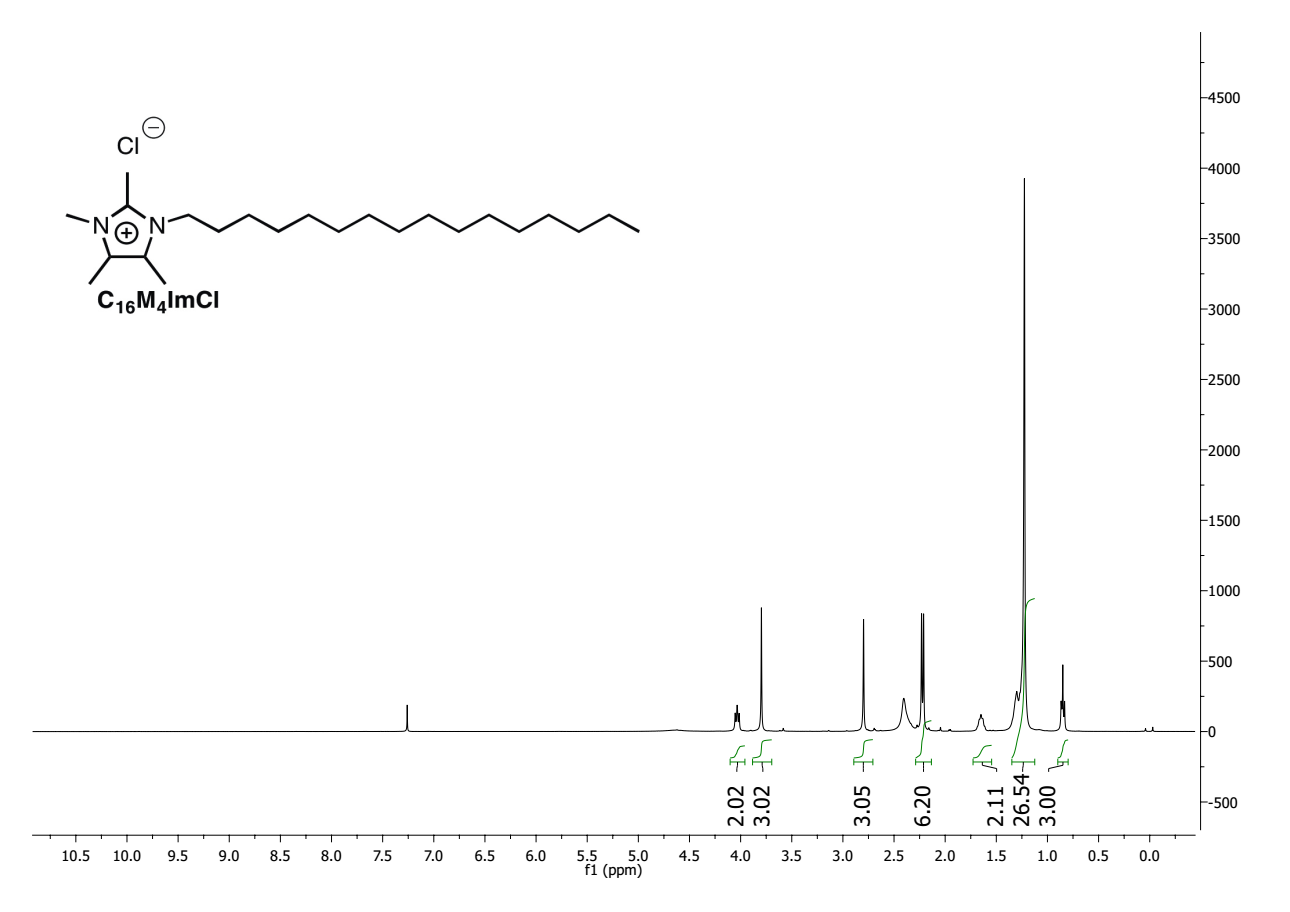


**Figure S4.** ^1^H NMR spectrum of **C_16_M_4_ImCl** (400 MHz, CDCl_3_).


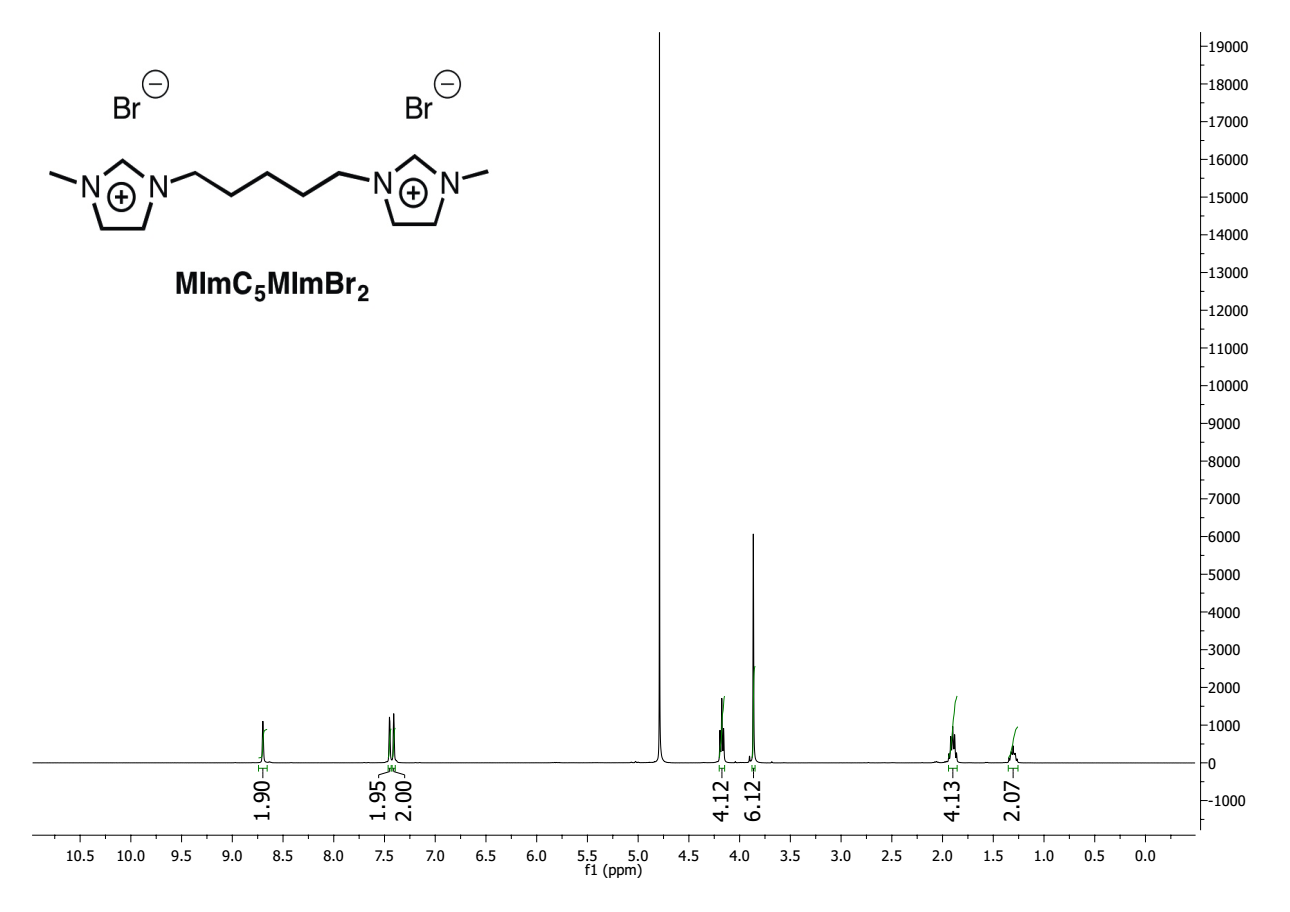


**Figure S5.** ^1^H NMR spectrum of **MImC_5_MImBr_2_** (400 MHz, D_2_O).


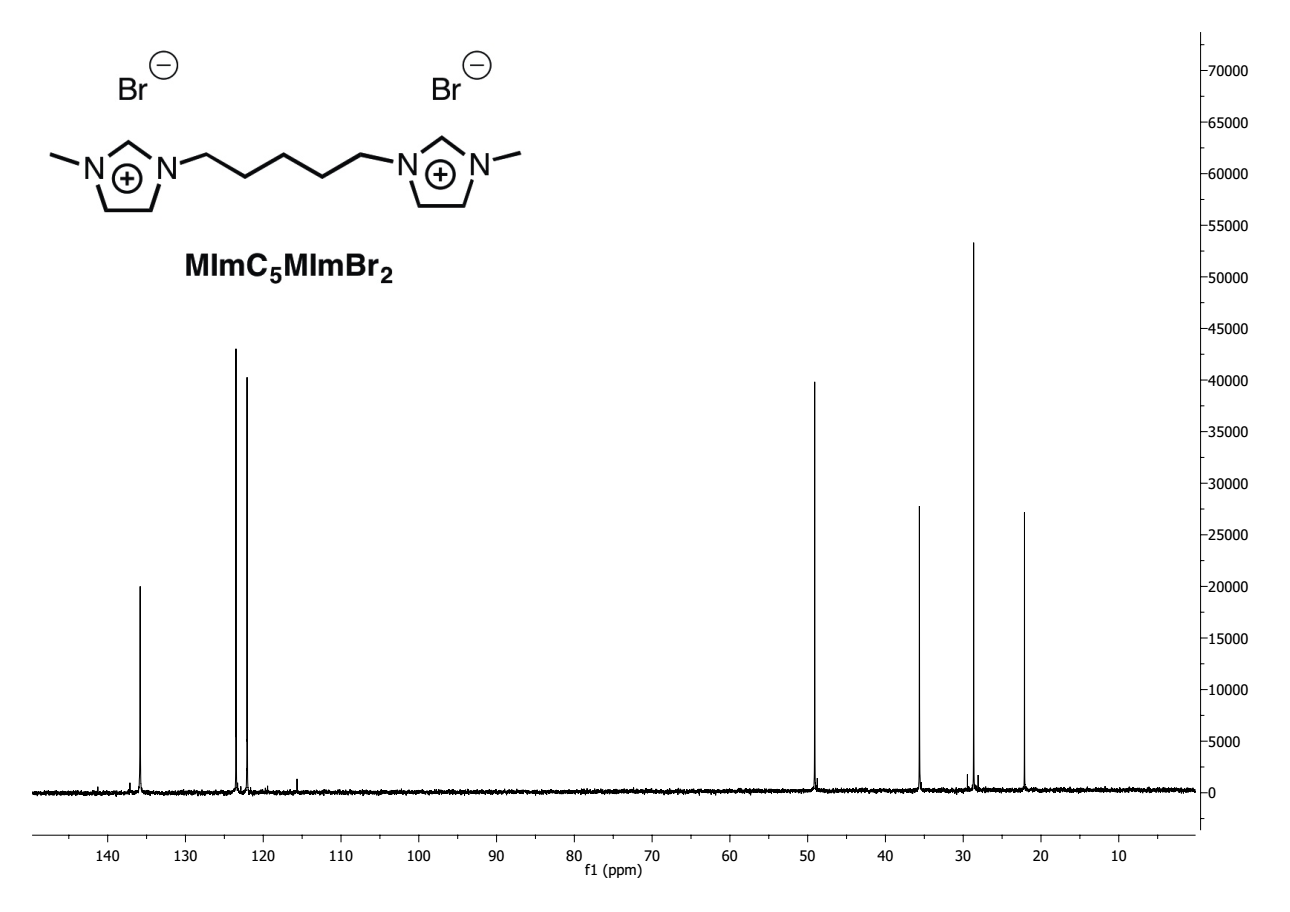


**Figure S6.** ^13^C NMR spectrum of **MImC_5_MImBr_2_** (101 MHz, D_2_O).


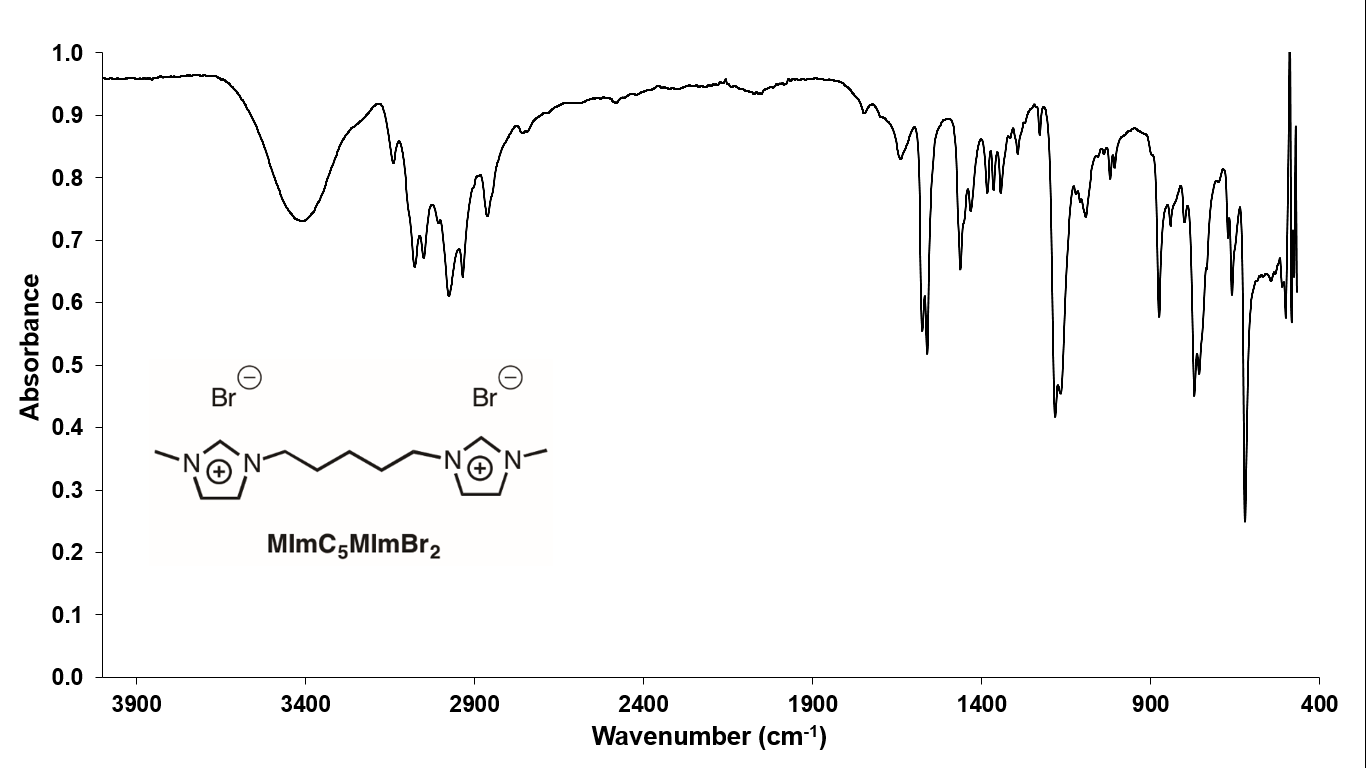


**Figure S7.** FTIR spectrum of **MImC_5_MImBr_2_**.


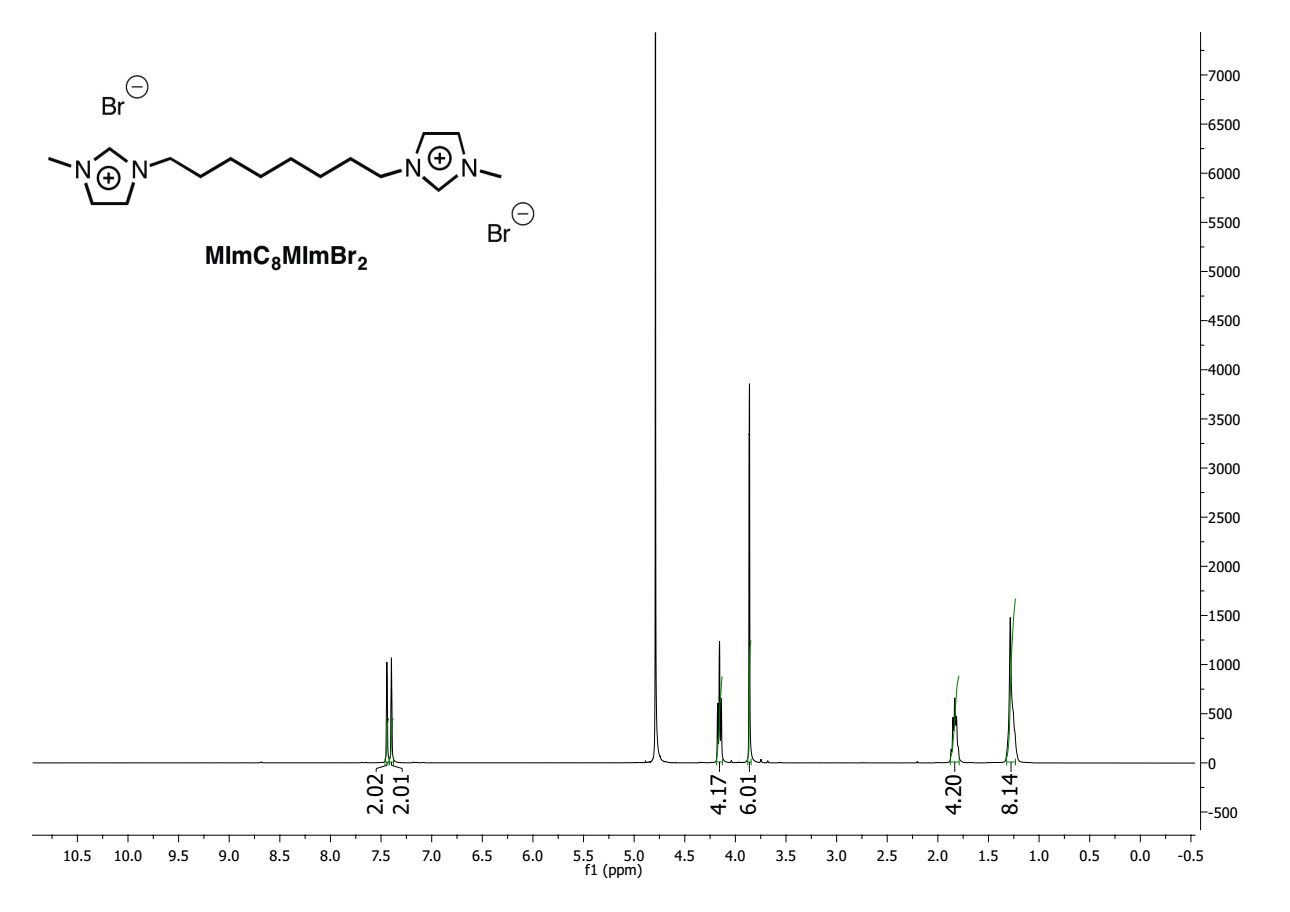


**Figure S8.** ^1^H NMR spectrum of **MImC_8_MImBr_2_** (400 MHz, D_2_O).


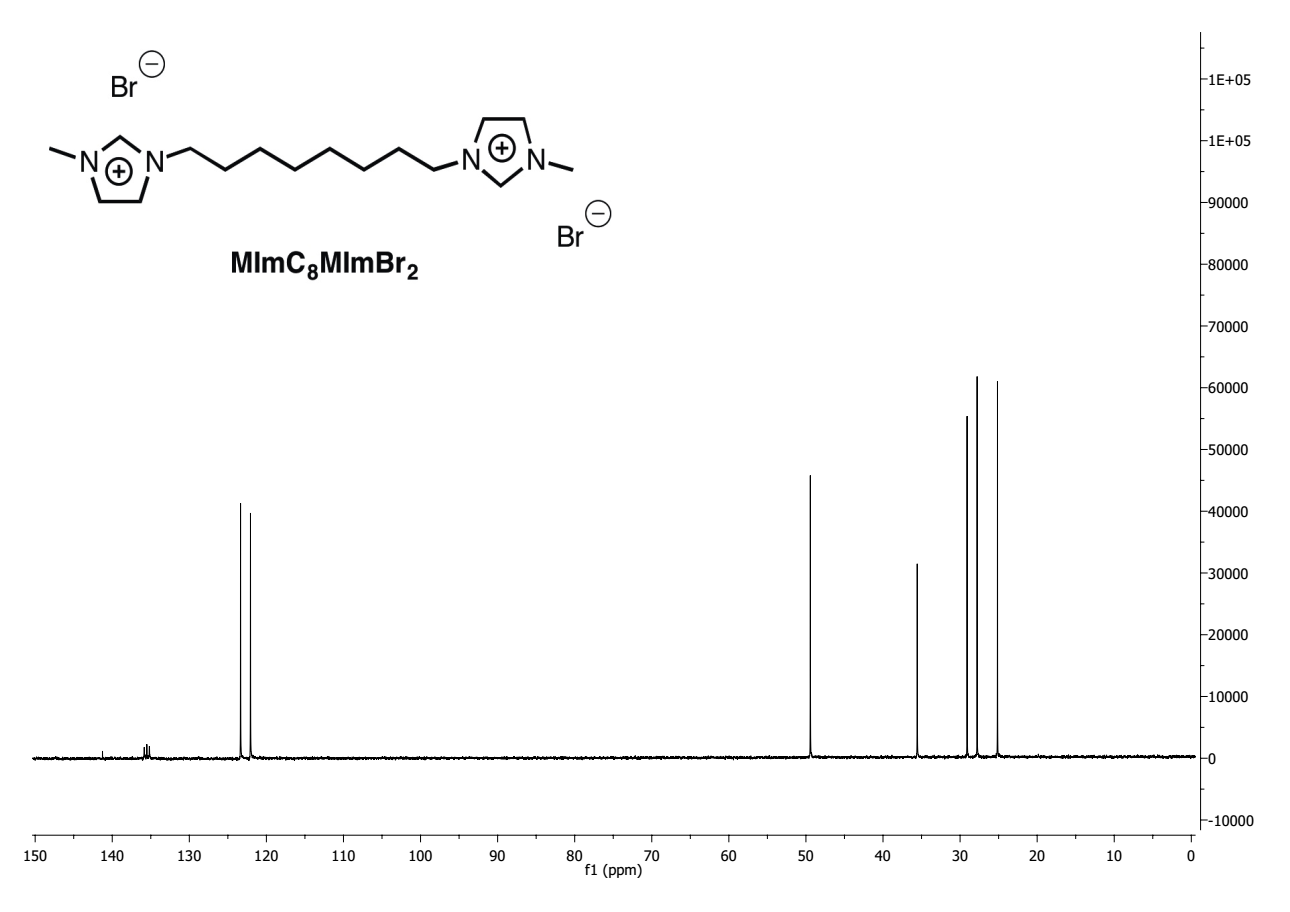


**Figure S9.** ^13^C NMR spectrum of **MImC_8_MImBr_2_** (101 MHz, D_2_O).


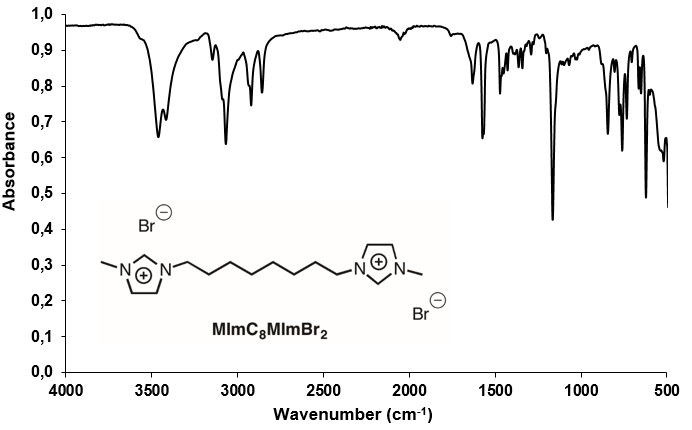


**Figure S10.** FTIR spectrum of **MImC_8_MImBr_2_**.


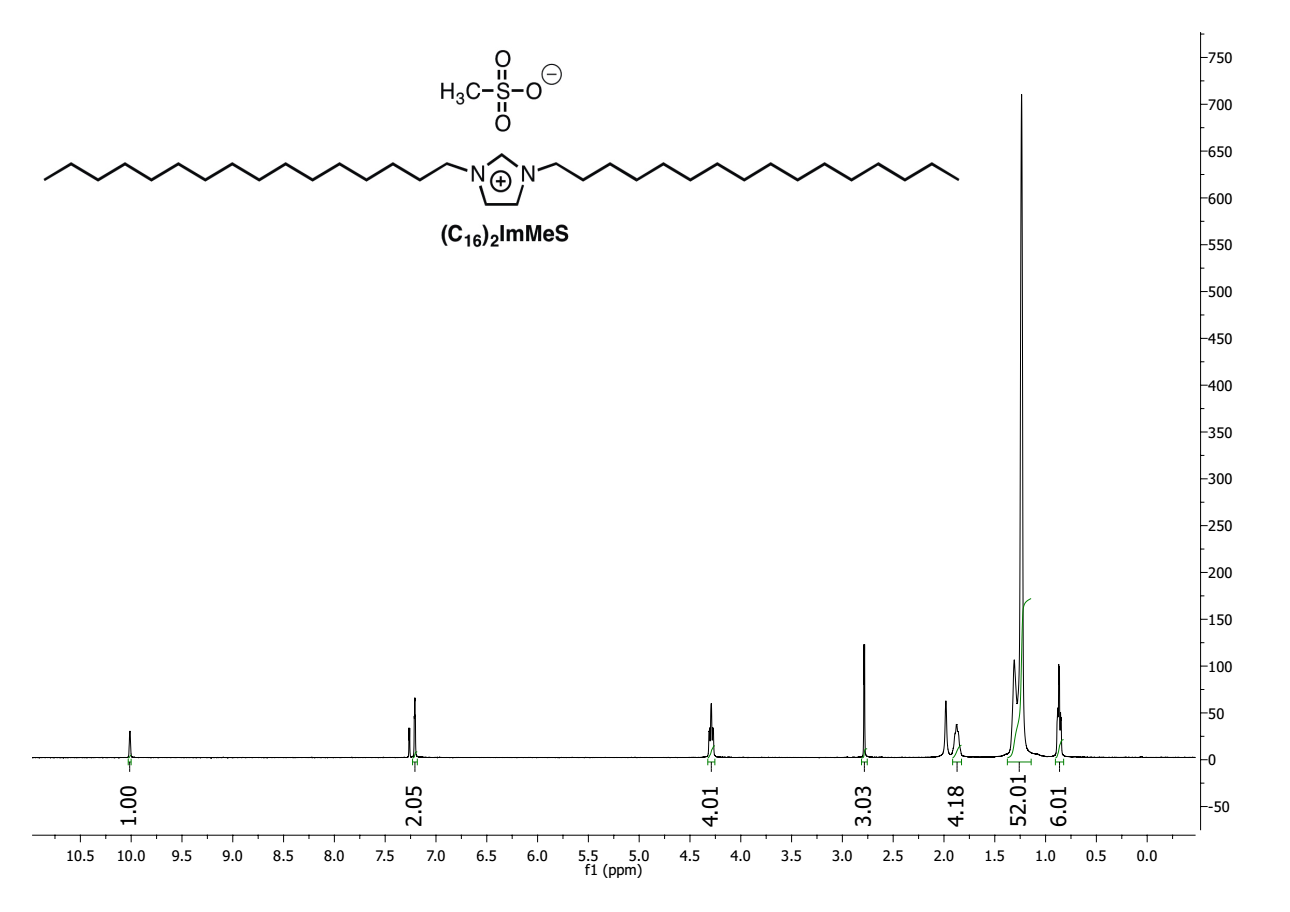


**Figure S11.** ^1^H NMR spectrum of **(C_16_)_2_ImMeS** (400 MHz, CDCl_3_).


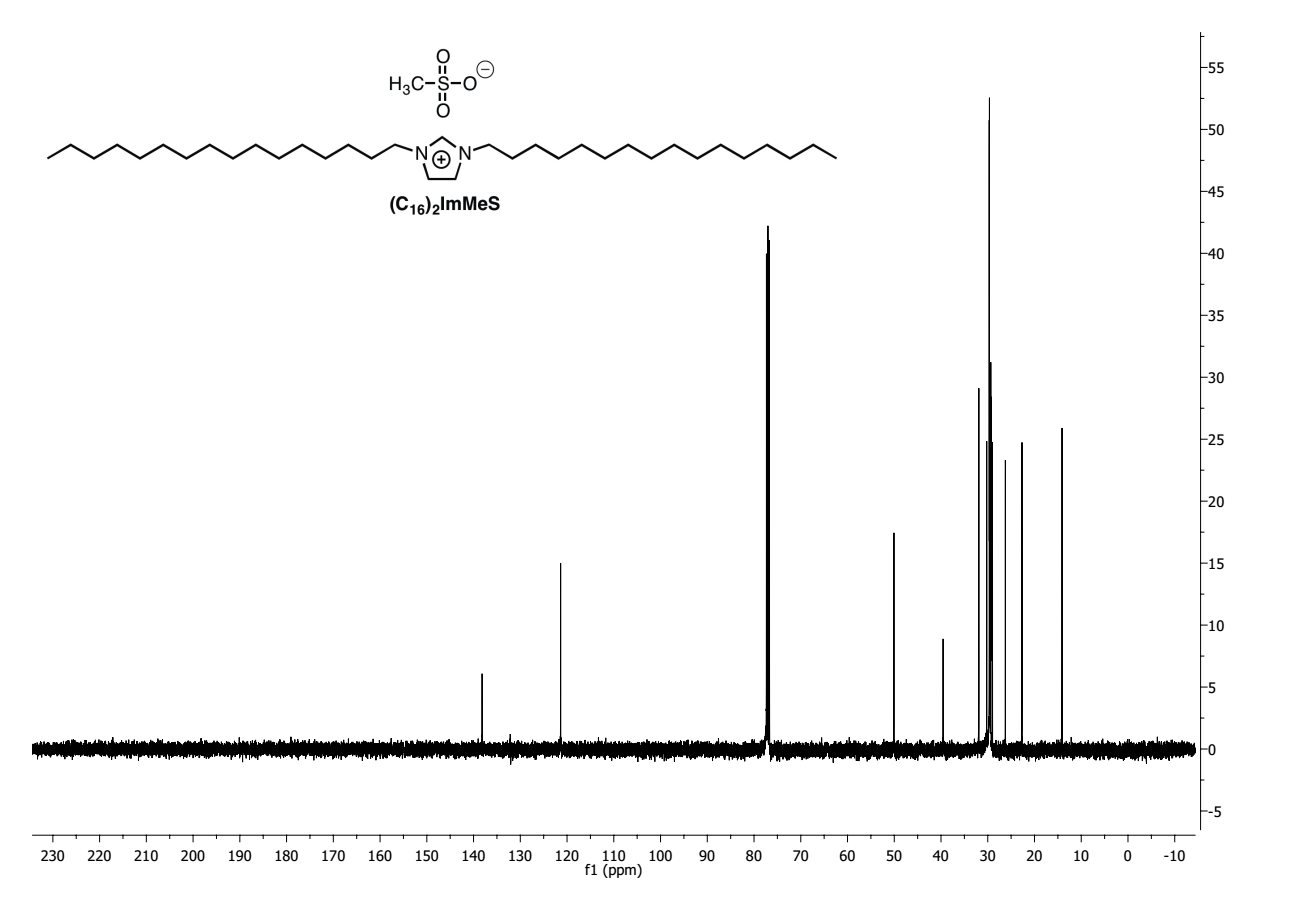


**Figure S12.** ^13^C NMR spectrum of **(C_16_)_2_ImMeS** (101 MHz, CDCl_3_).


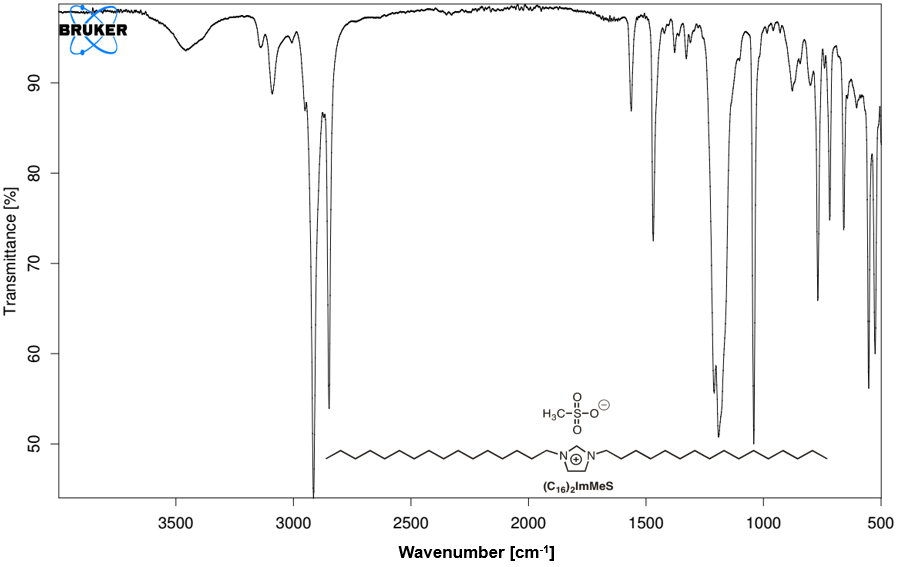


**Figure S13.** FTIR spectrum of **(C_16_)_2_ImMeS**.


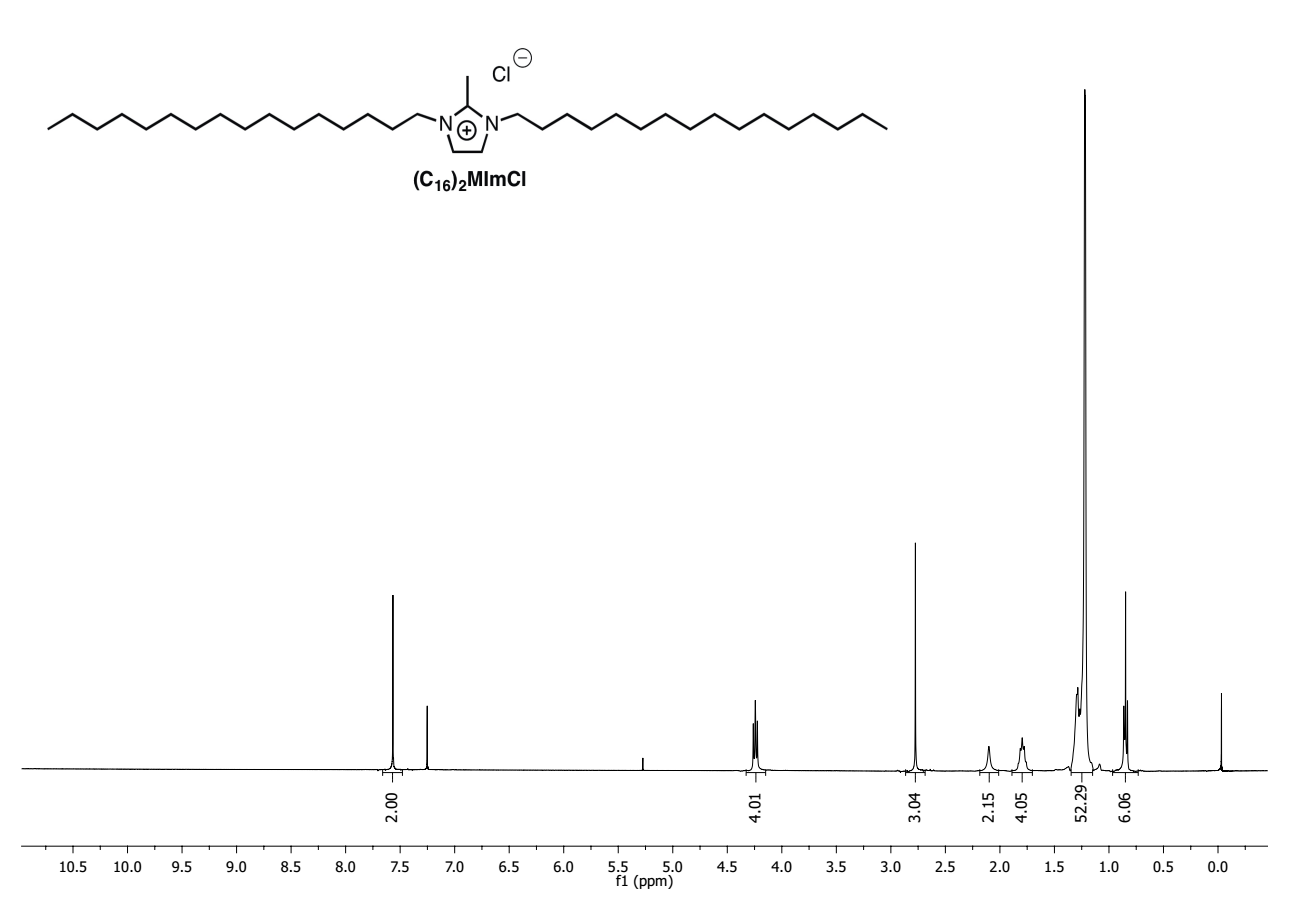


**Figure S14.** ^1^H NMR spectrum of **(C_16_)_2_MImCl** (400 MHz, CDCl_3_).


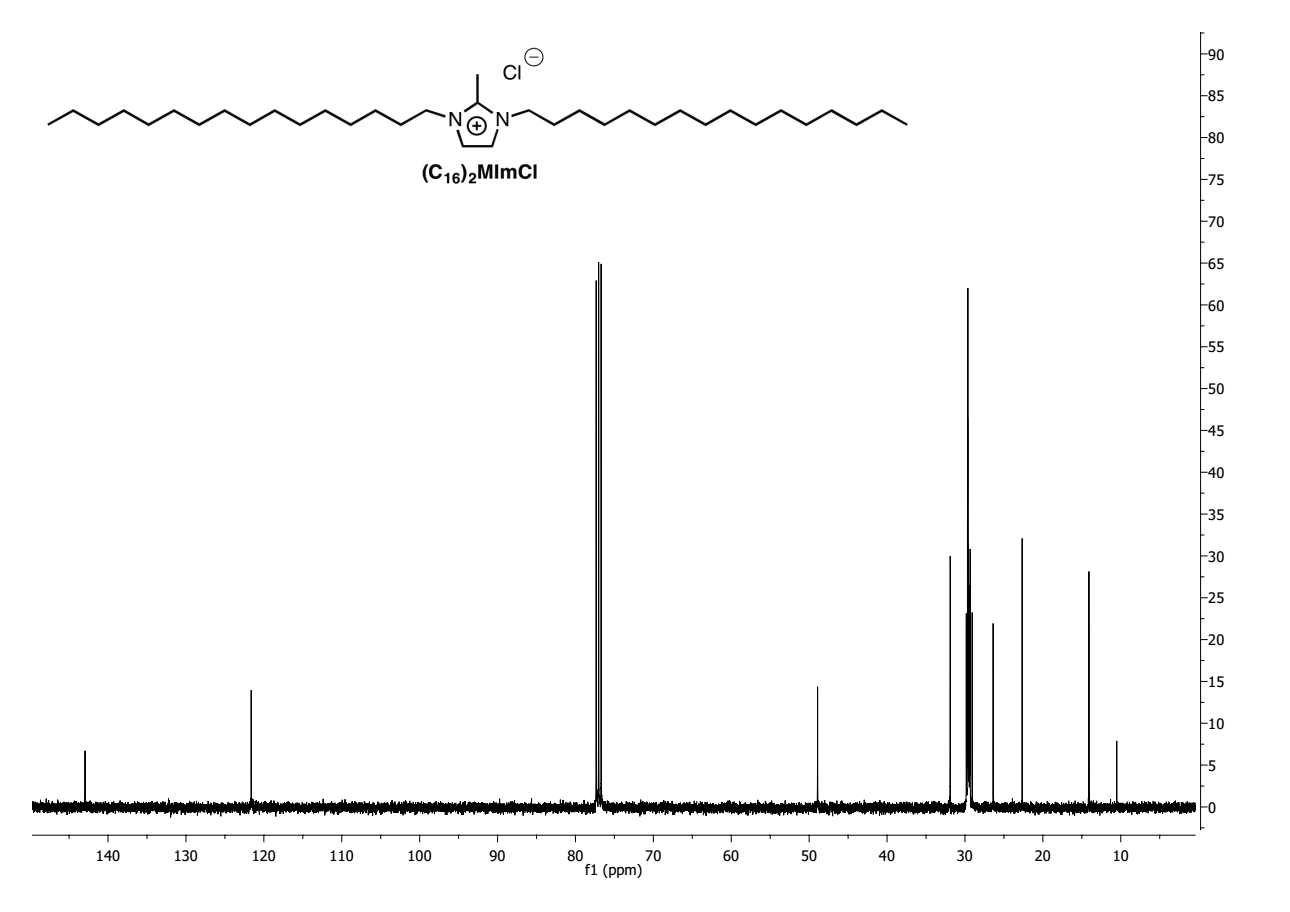


**Figure S15.** ^13^C NMR spectrum of **(C_16_)_2_MImCl** (101 MHz, CDCl_3_).


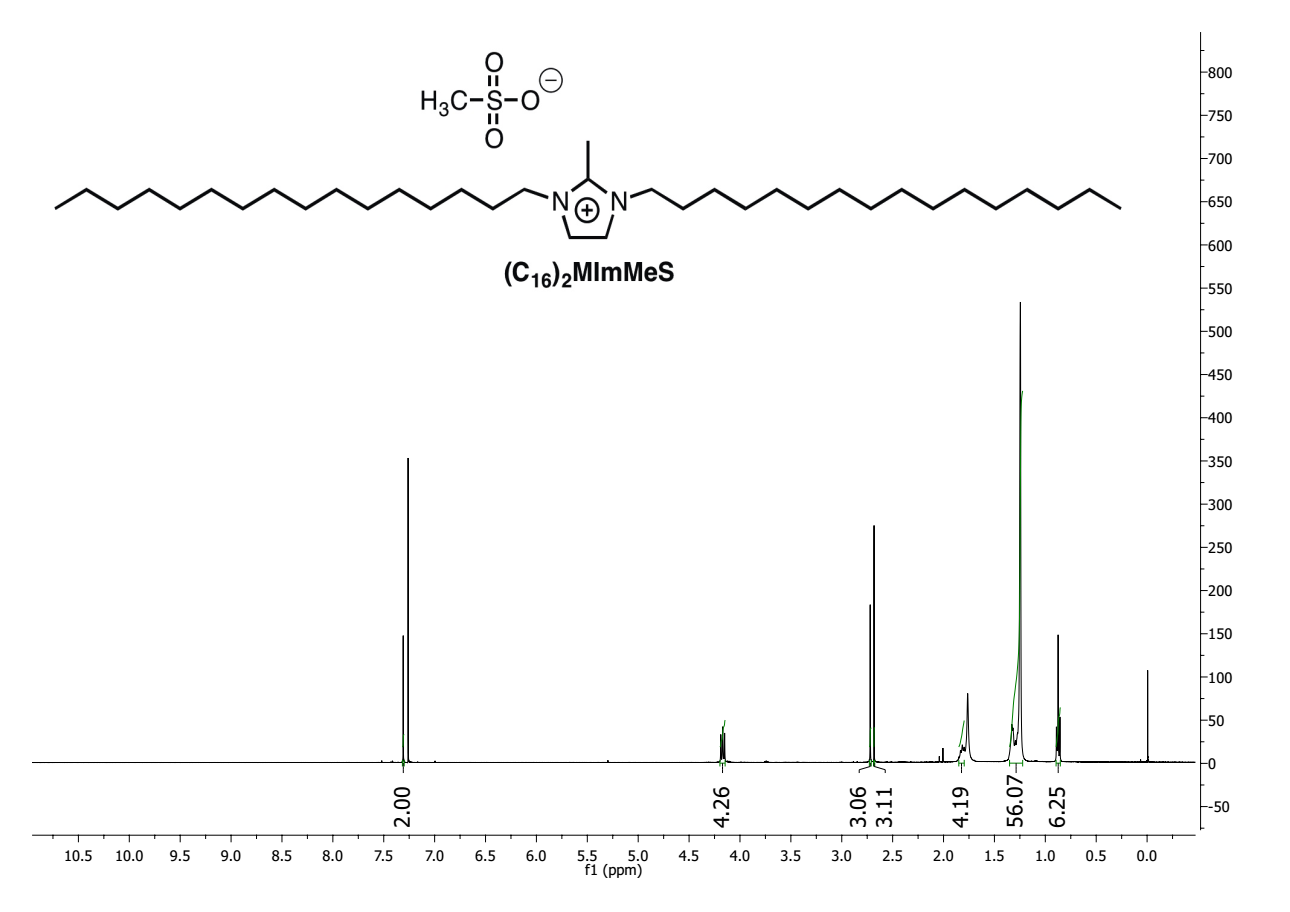


**Figure S16.** ^1^H NMR spectrum of **(C_16_)_2_MImMeS** (400 MHz, CDCl_3_).


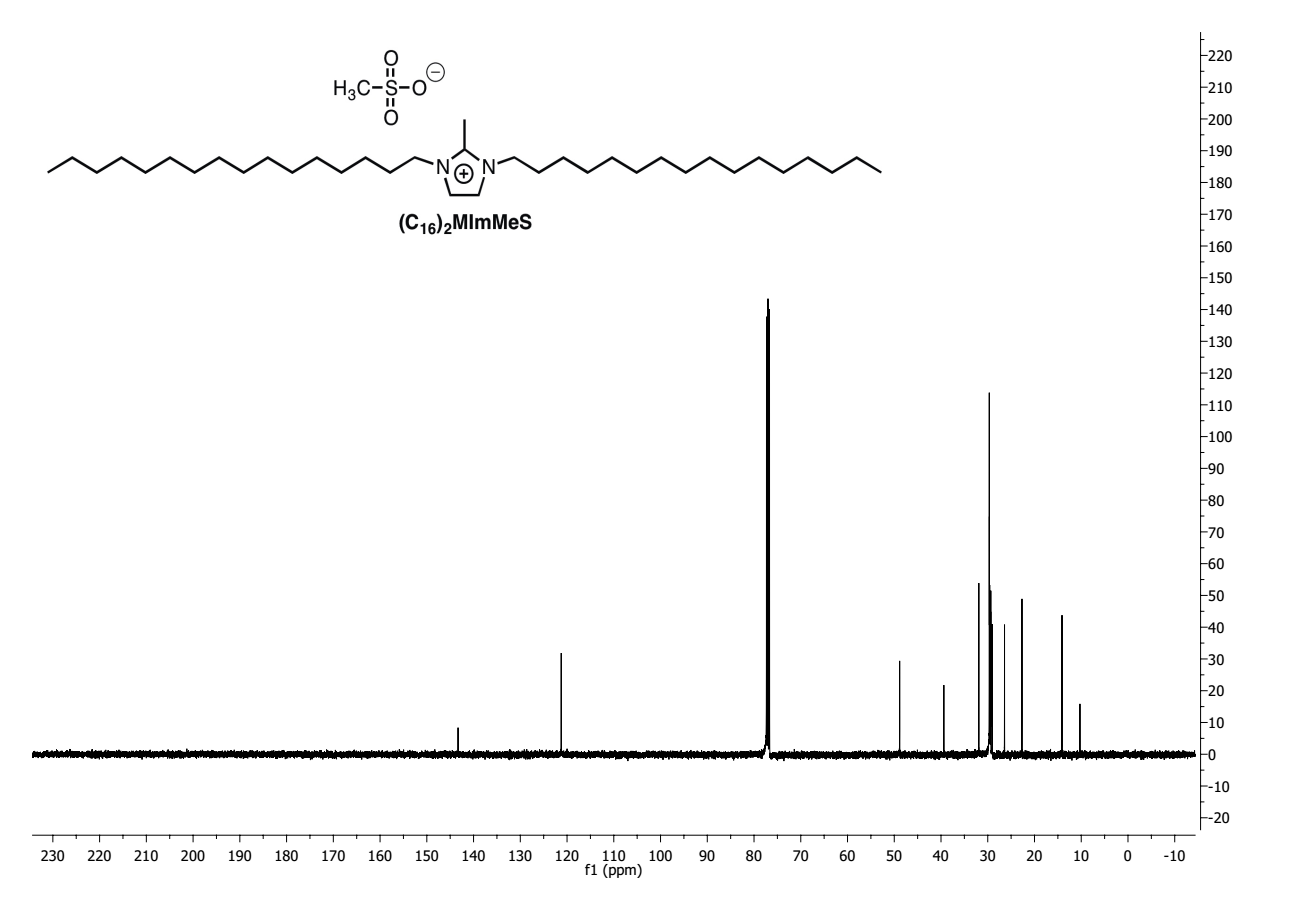


**Figure S17.** ^13^C NMR spectrum of **(C_16_)_2_MImMeS** (101 MHz, CDCl_3_).


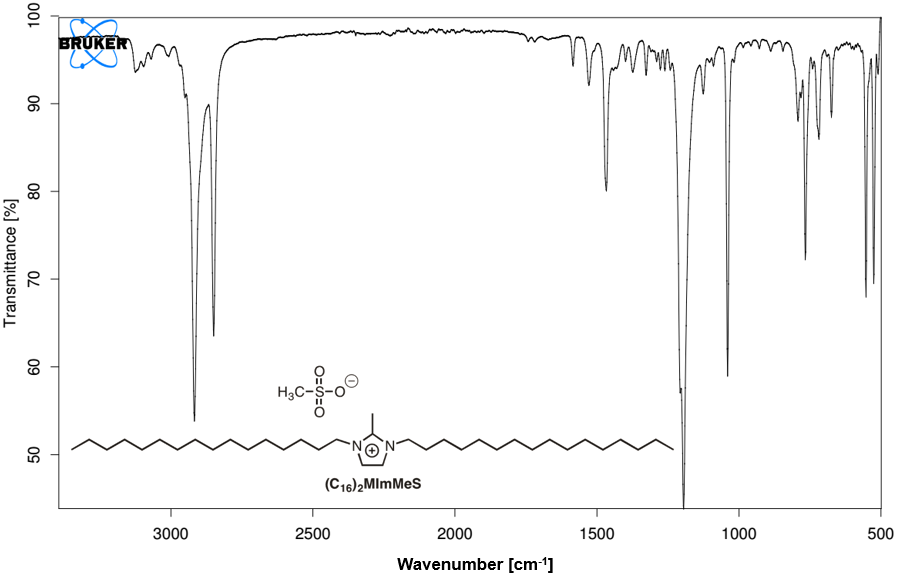


**Figure S18.** FTIR spectrum of **(C_16_)_2_MImMeS**.


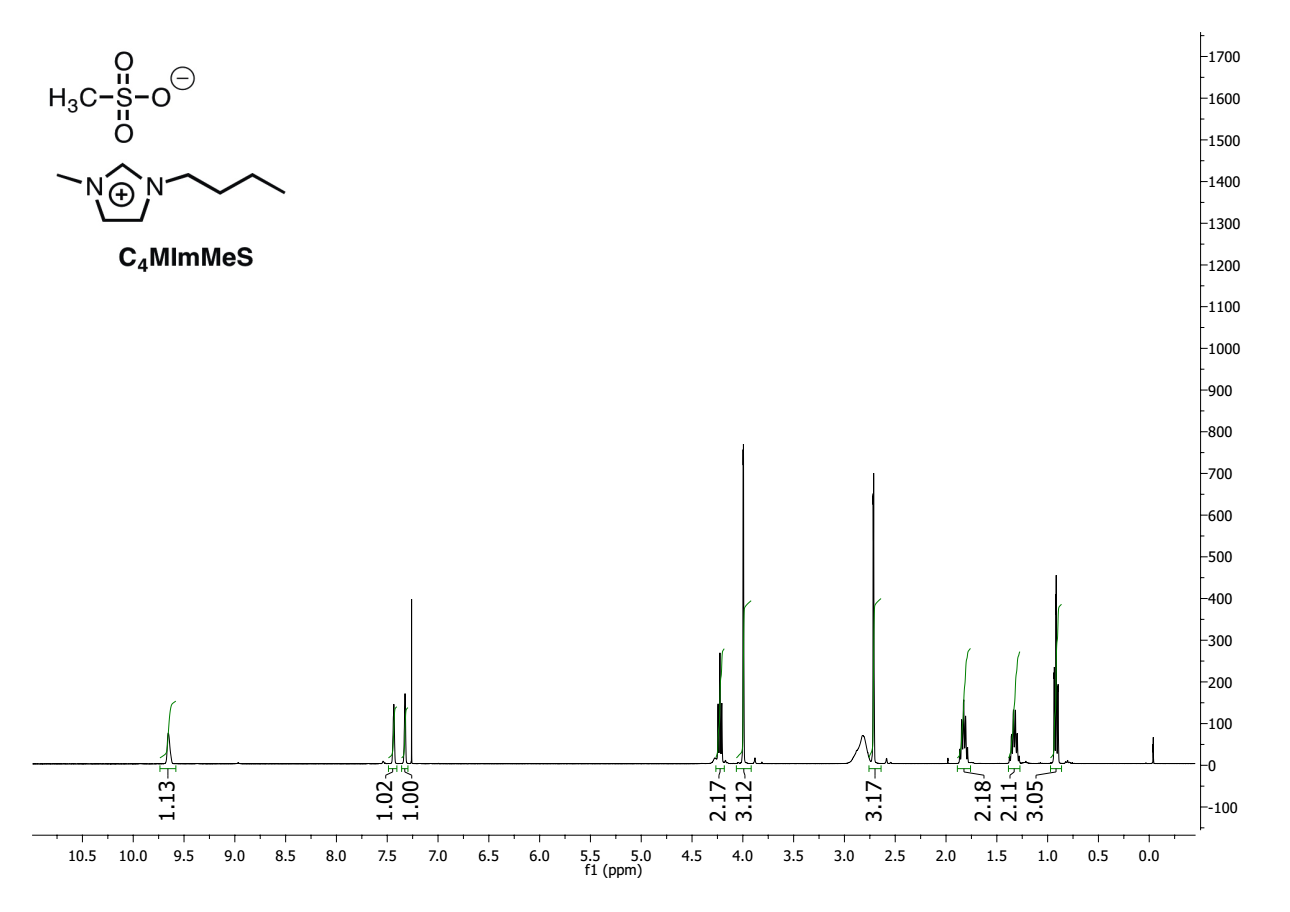


**Figure S19.** ^1^H NMR spectrum of **C_4_MImMeS** (400 MHz, CDCl_3_).


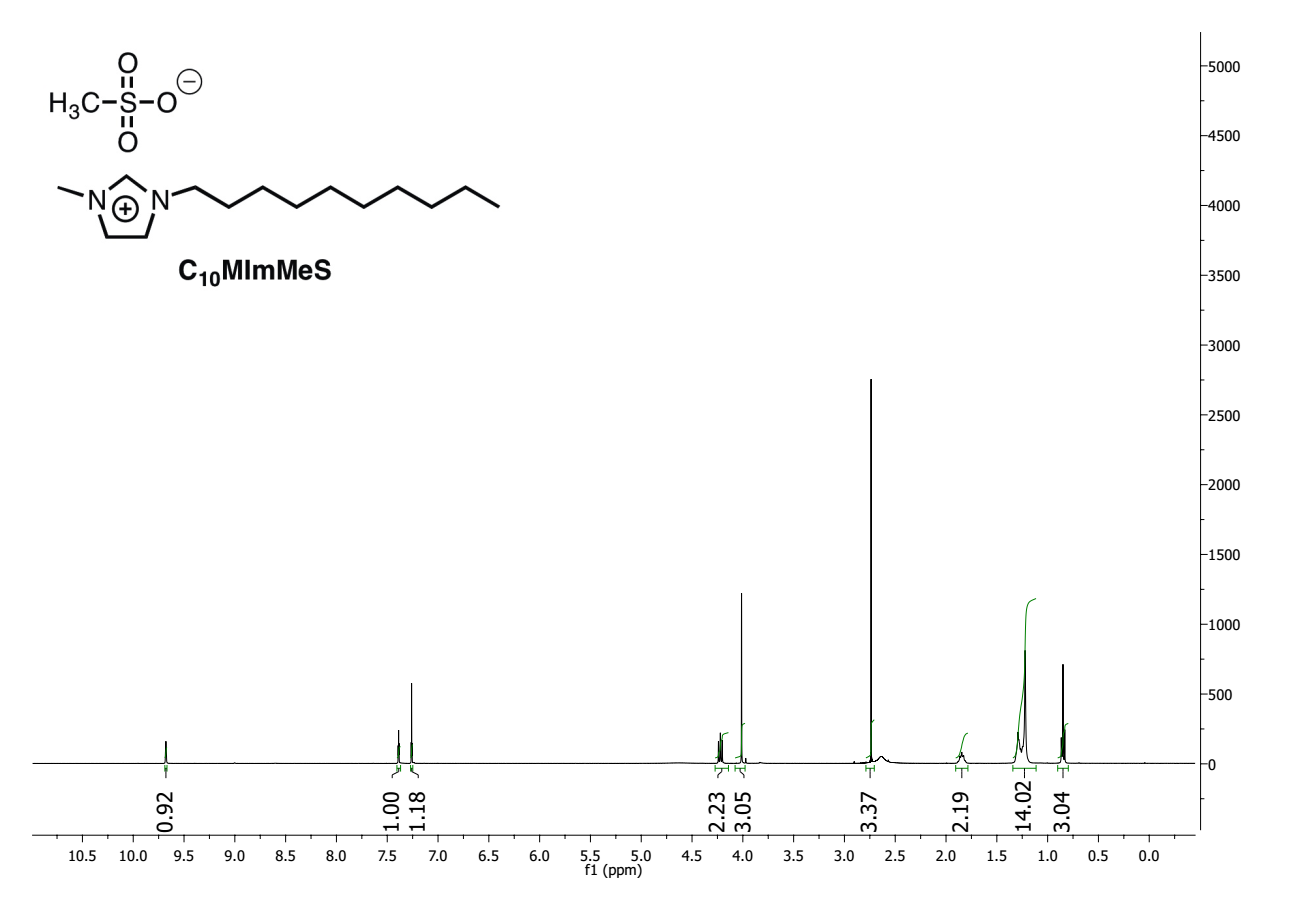


**Figure S20.** ^1^H NMR spectrum of **C_10_MImMeS** (400 MHz, CDCl_3_).


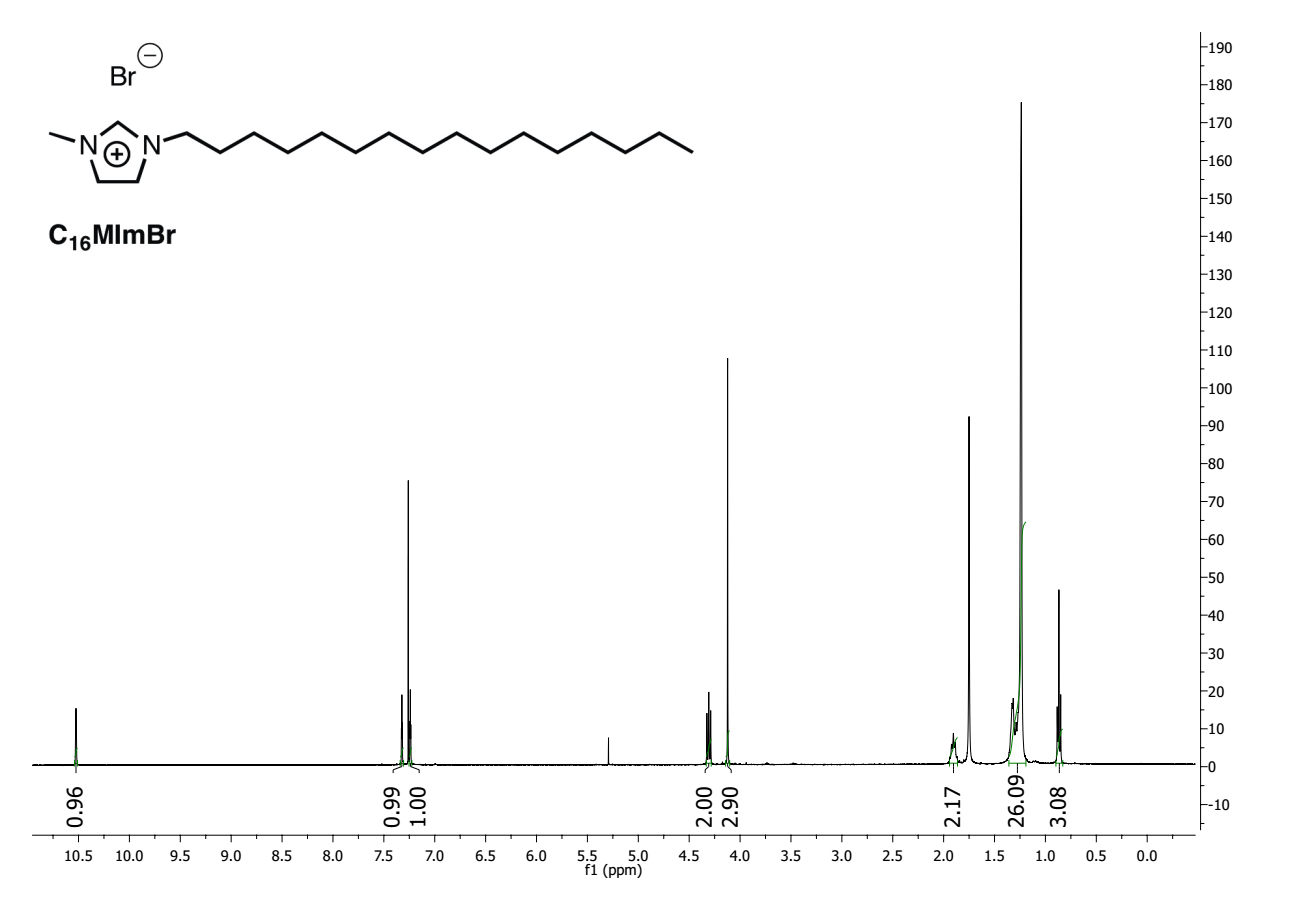


**Figure S21.** ^1^H NMR spectrum of **C_16_MImBr** (400 MHz, CDCl_3_).


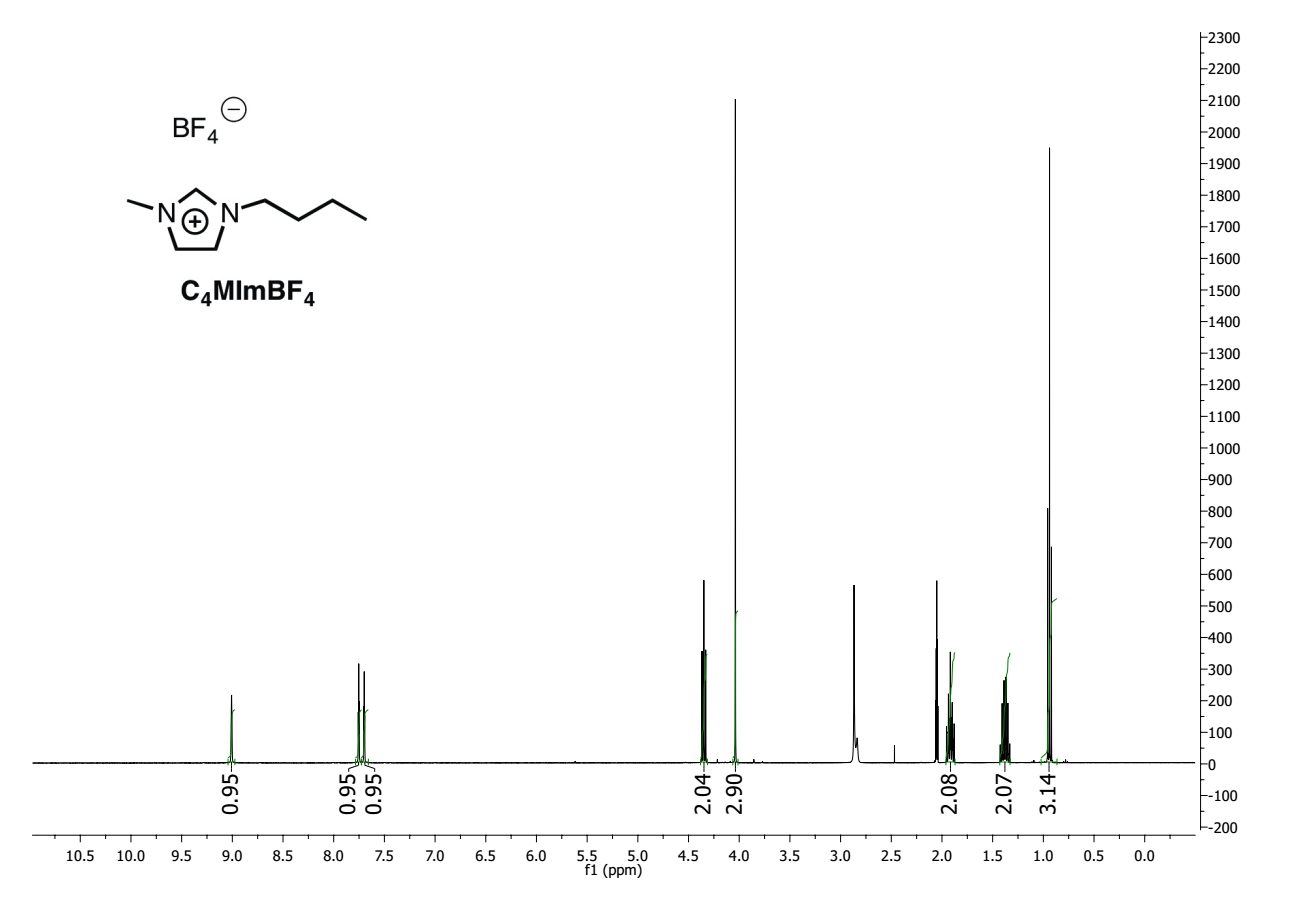


**Figure S22.** ^1^H NMR spectrum of **C_4_MImBF_4_** (400 MHz, acetone-d6).


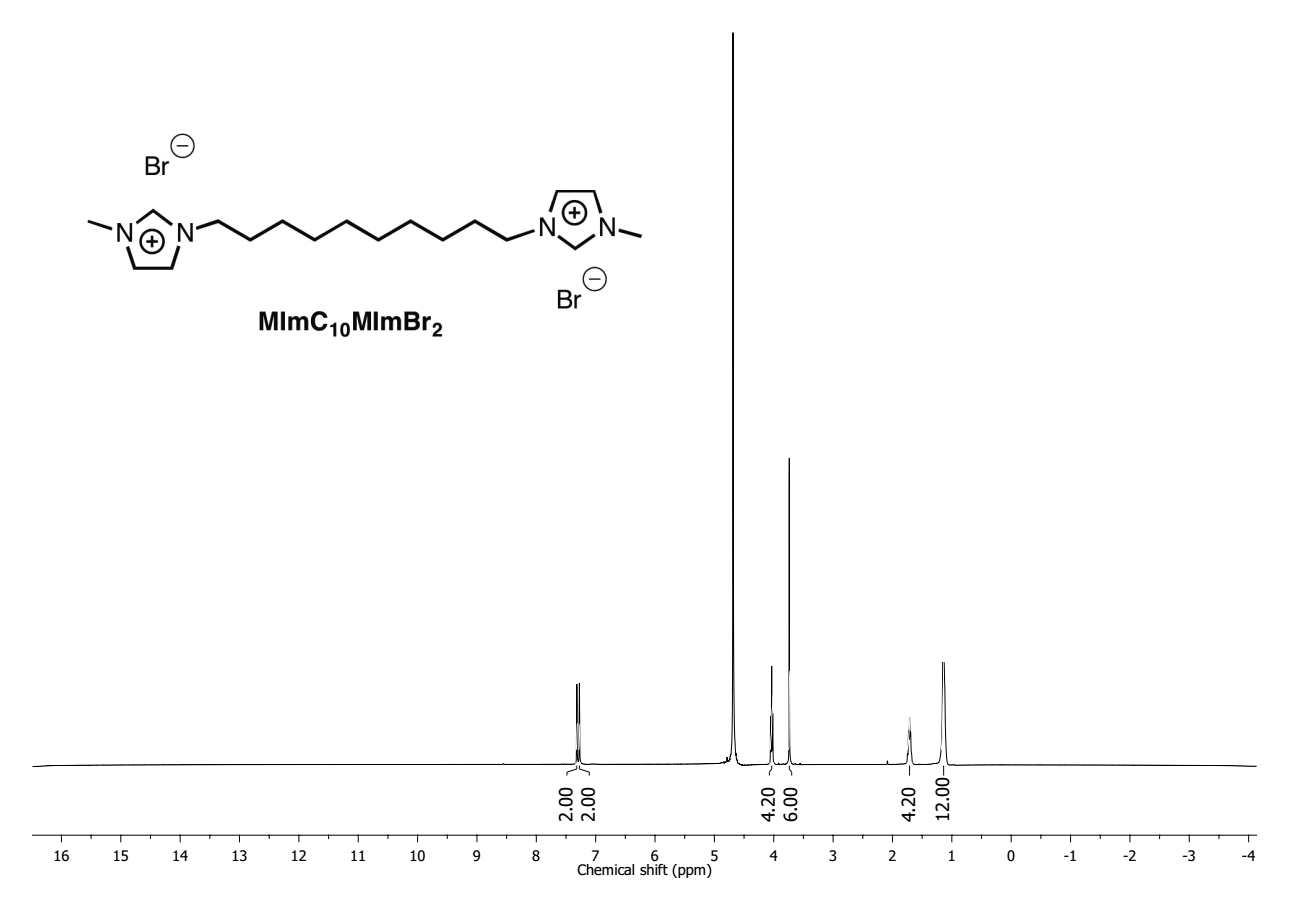


**Figure S23.** ^1^H NMR spectrum of **MImC_10_MImBr_2_** (400 MHz, D_2_O).


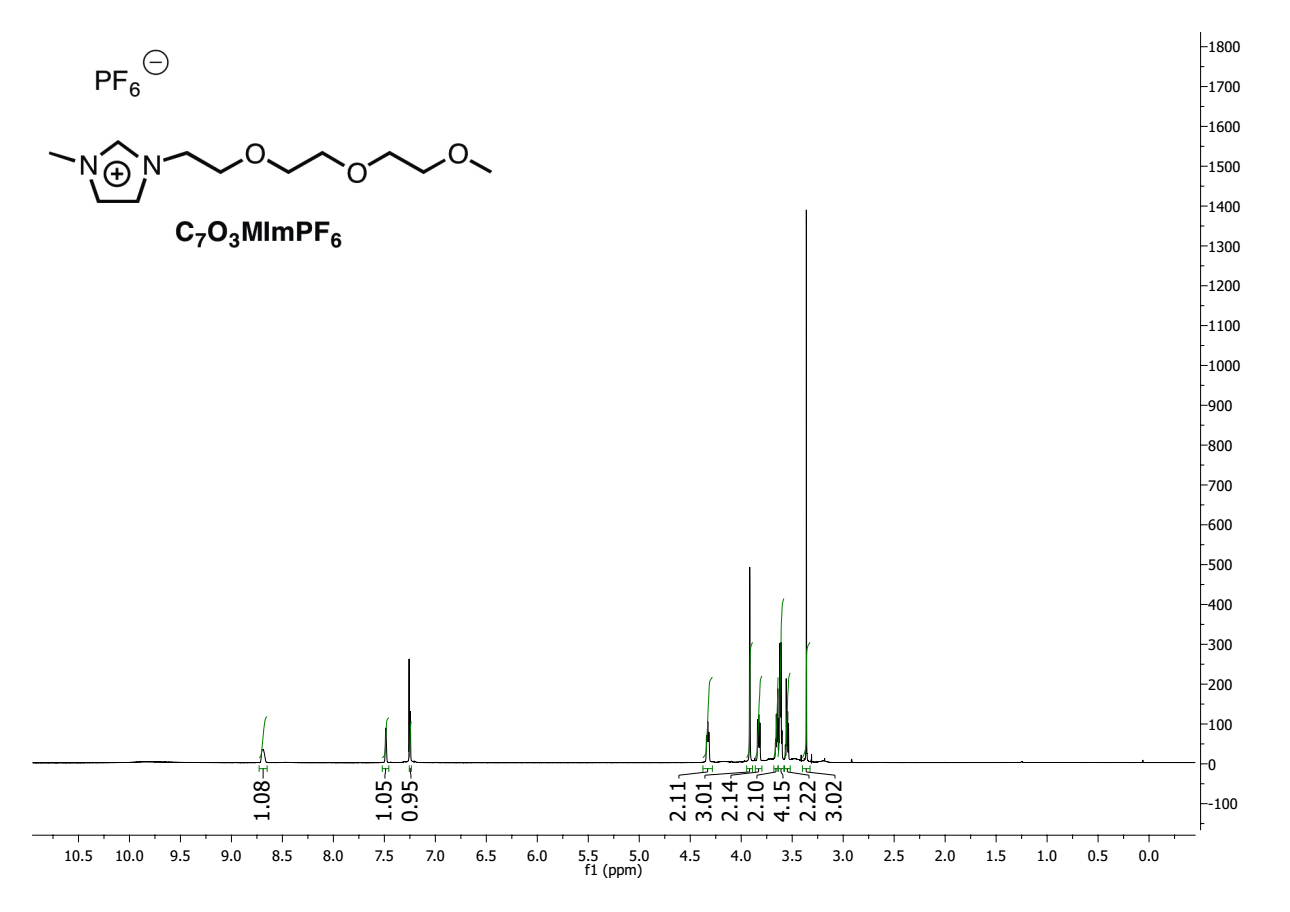


**Figure S24.** ^1^H NMR spectrum of **C_7_O_3_MImPF_6_** (400 MHz, CDCl_3_).


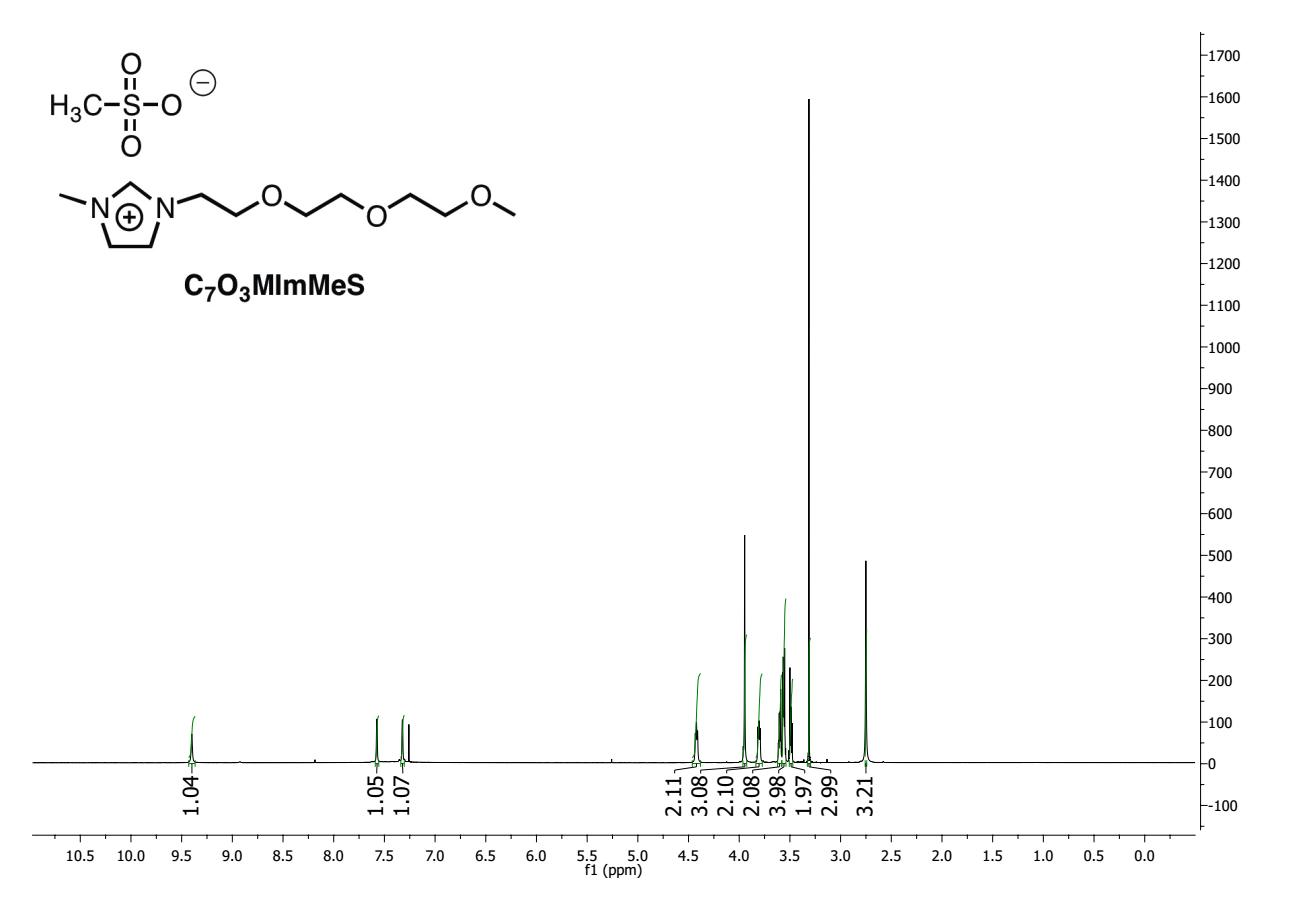


**Figure S25.** ^1^H NMR spectrum of **C_7_O_3_MImMeS** (400 MHz, CDCl_3_).


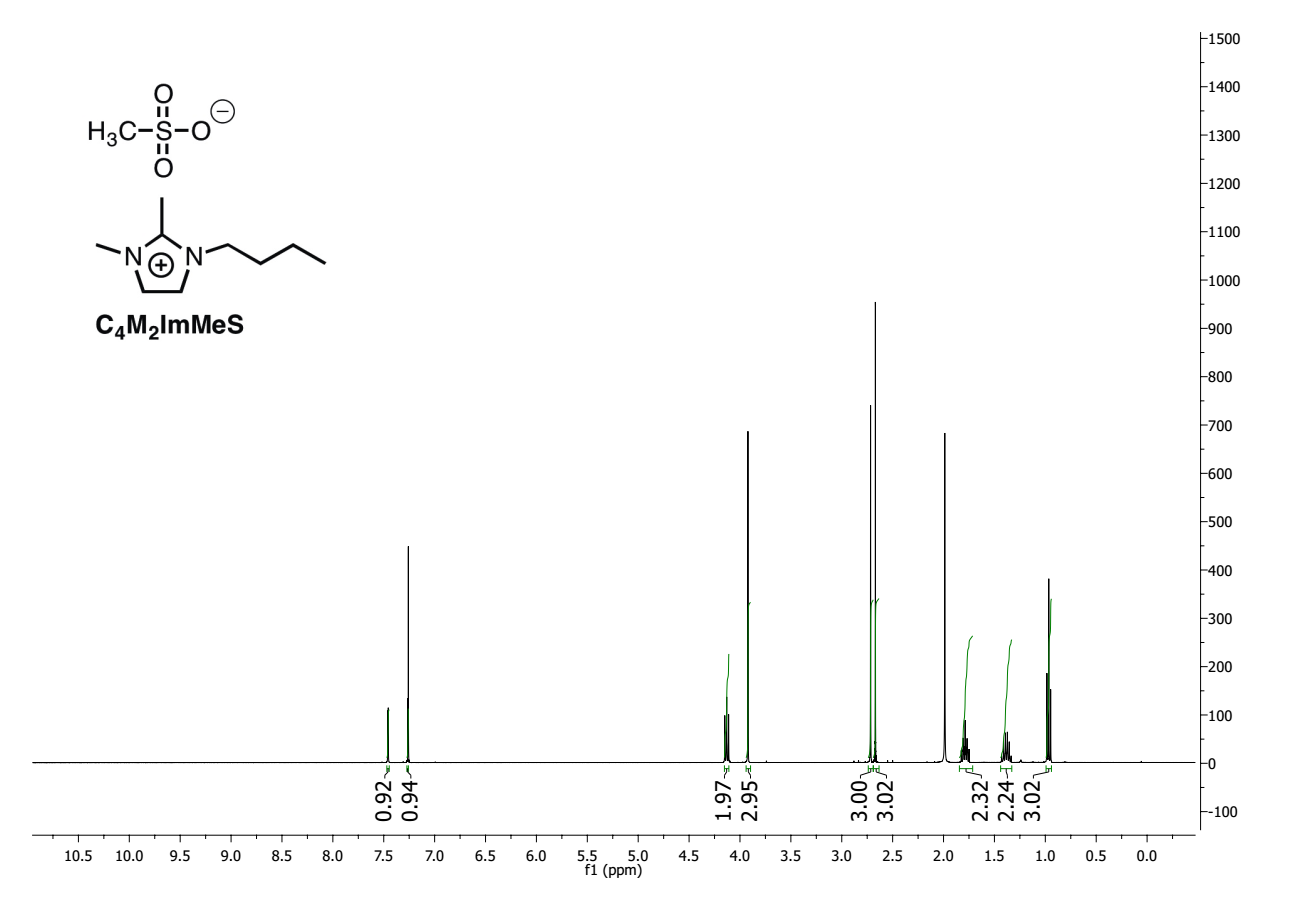


**Figure S26.** ^1^H NMR spectrum of **C_4_M_2_ImMeS** (400 MHz, CDCl_3_).


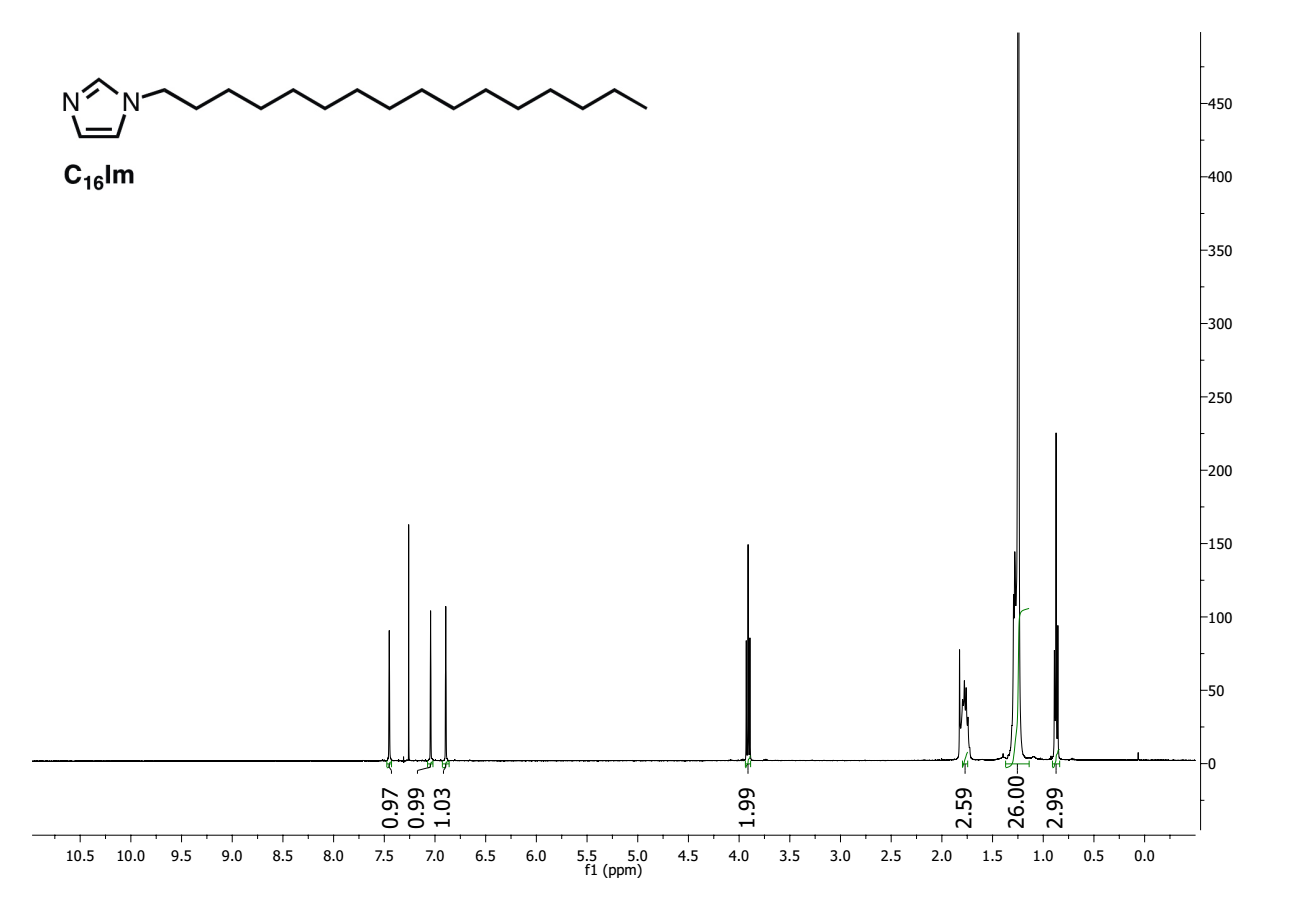


**Figure S27.** ^1^H NMR spectrum of **C_16_Im** (400 MHz, CDCl_3_).


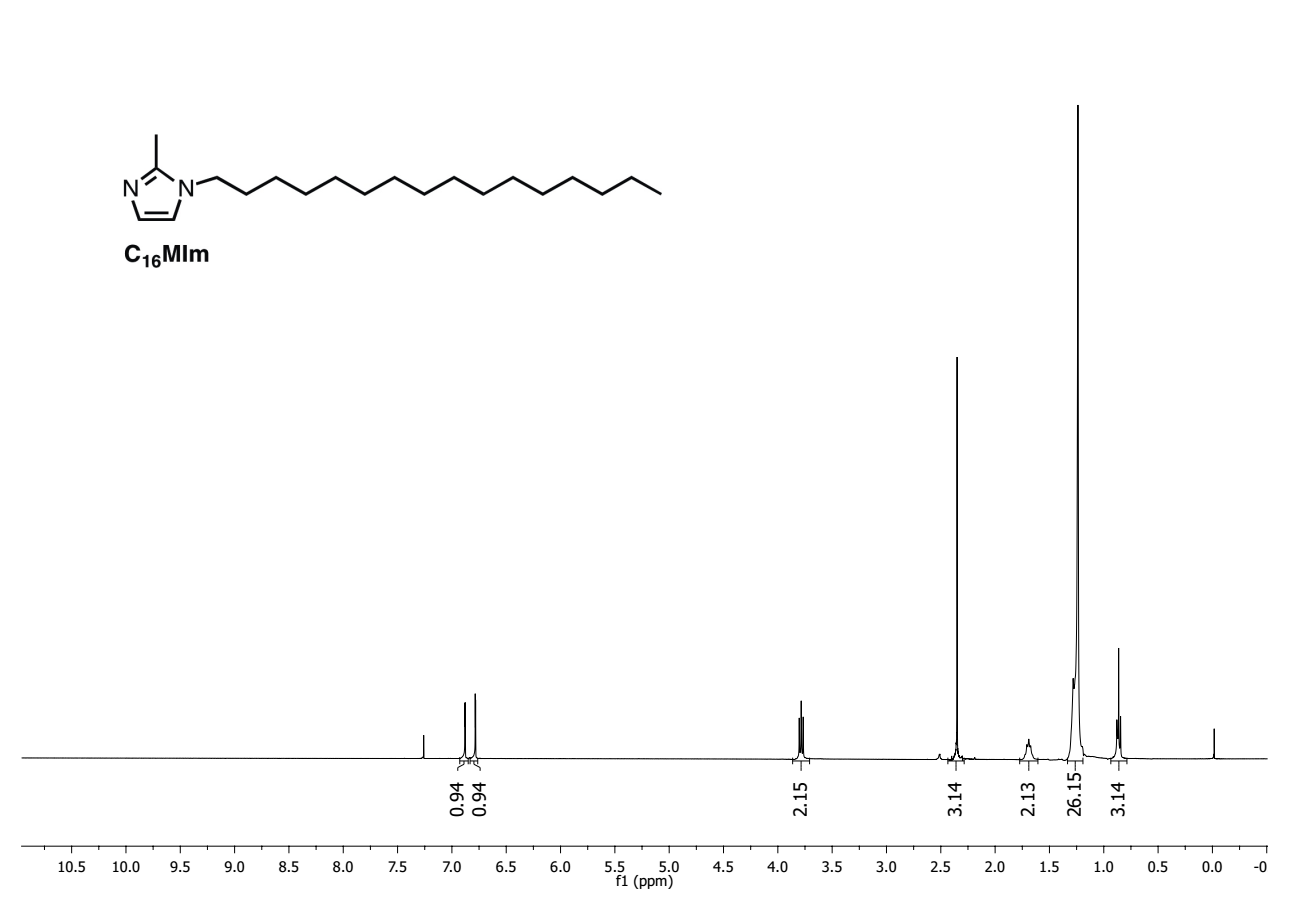


**Figure S28.** ^1^H NMR spectrum of **C_16_MIm** (400 MHz, CDCl_3_).


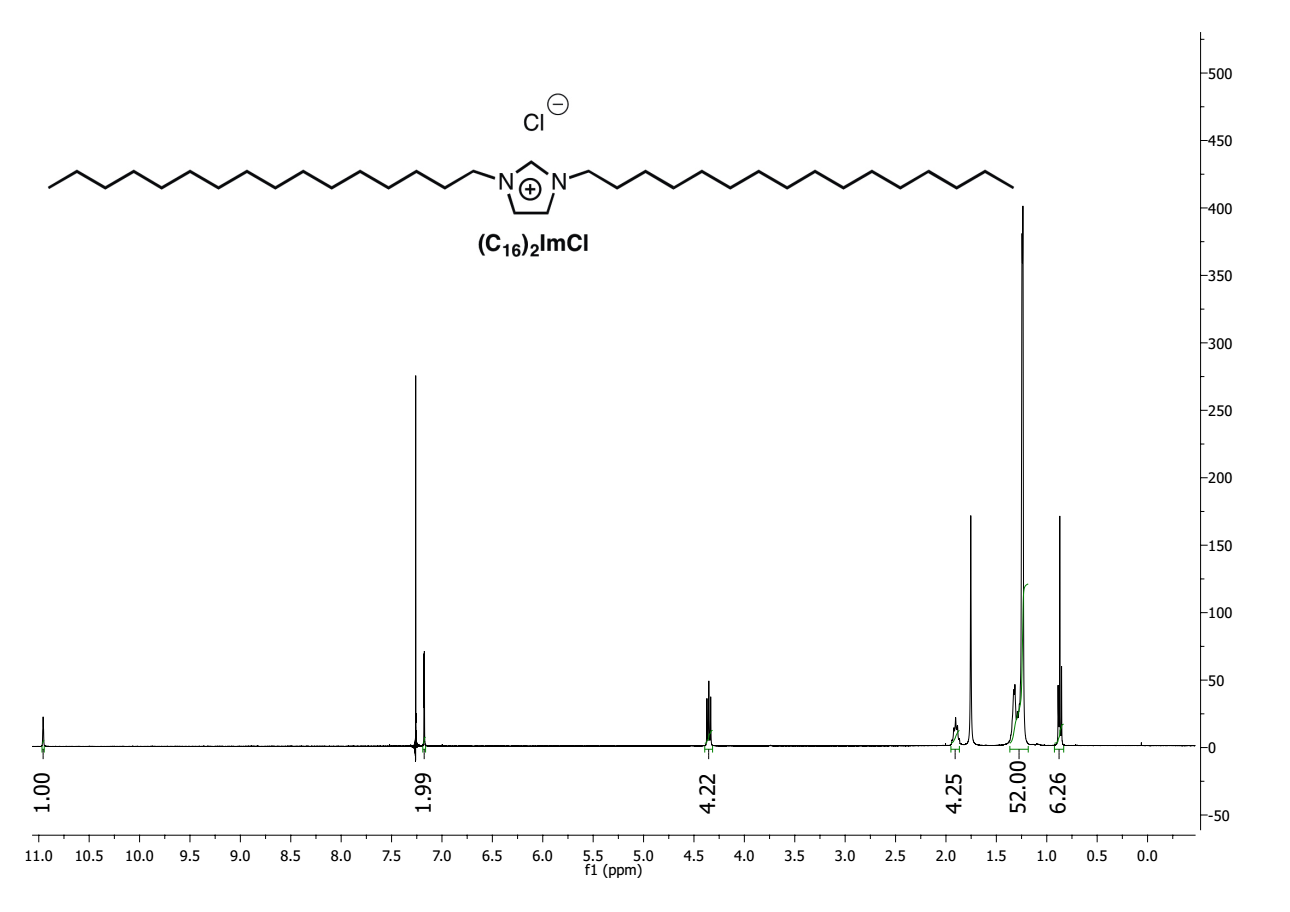


**Figure S29.** ^1^H NMR spectrum of **(C_16_)_2_ImCl** (400 MHz, CDCl_3_).


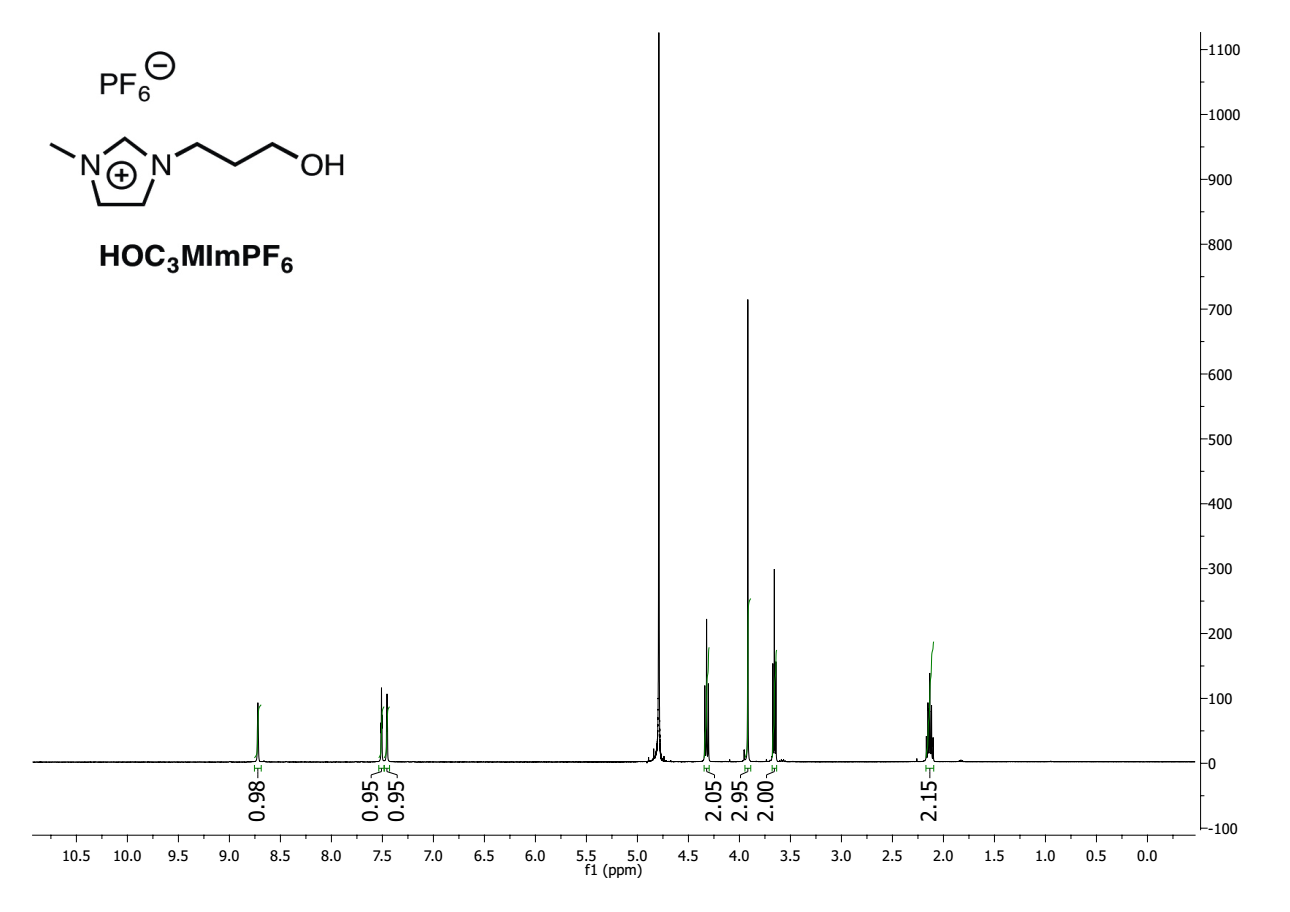


**Figure S30.** ^1^H NMR spectrum of **HOC_3_MImPF_6_** (400 MHz, D_2_O).


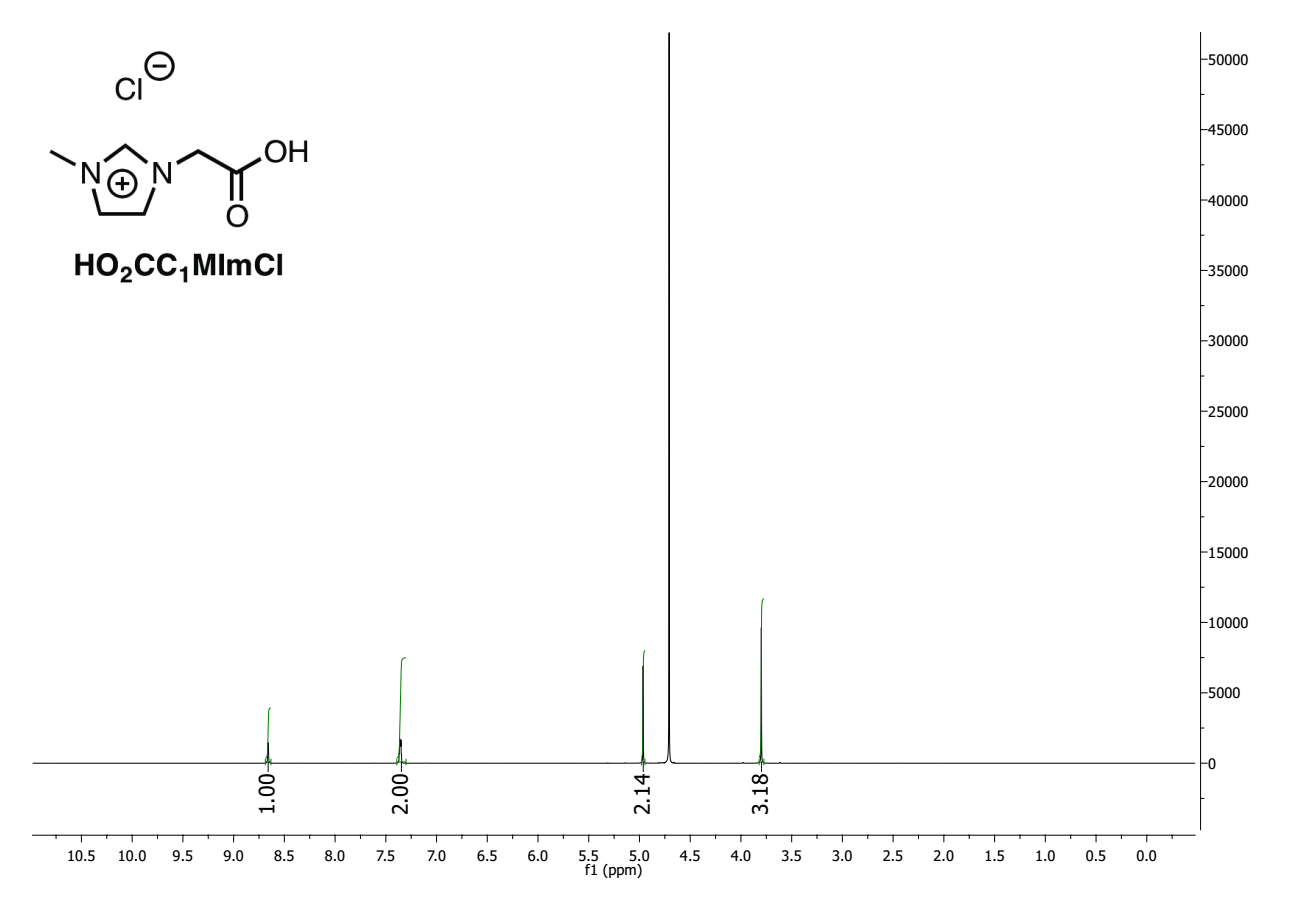


**Figure S31.** ^1^H NMR spectrum of **HO_2_CC_1_MImCl** (400 MHz, D_2_O).


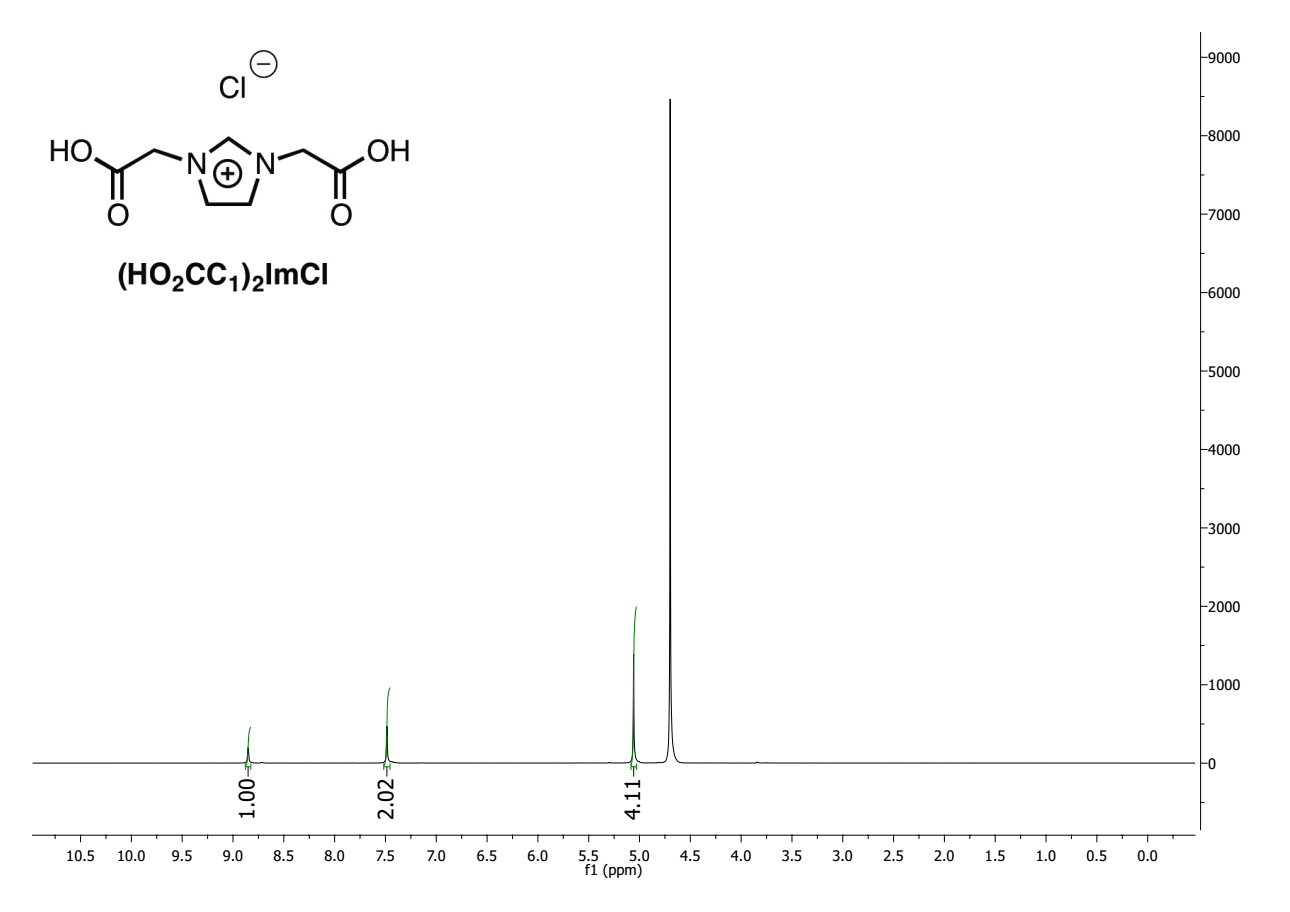


**Figure S32.** ^1^H NMR spectrum of **(HO_2_CC_1_)_2_ImCl** (400 MHz, D_2_O).


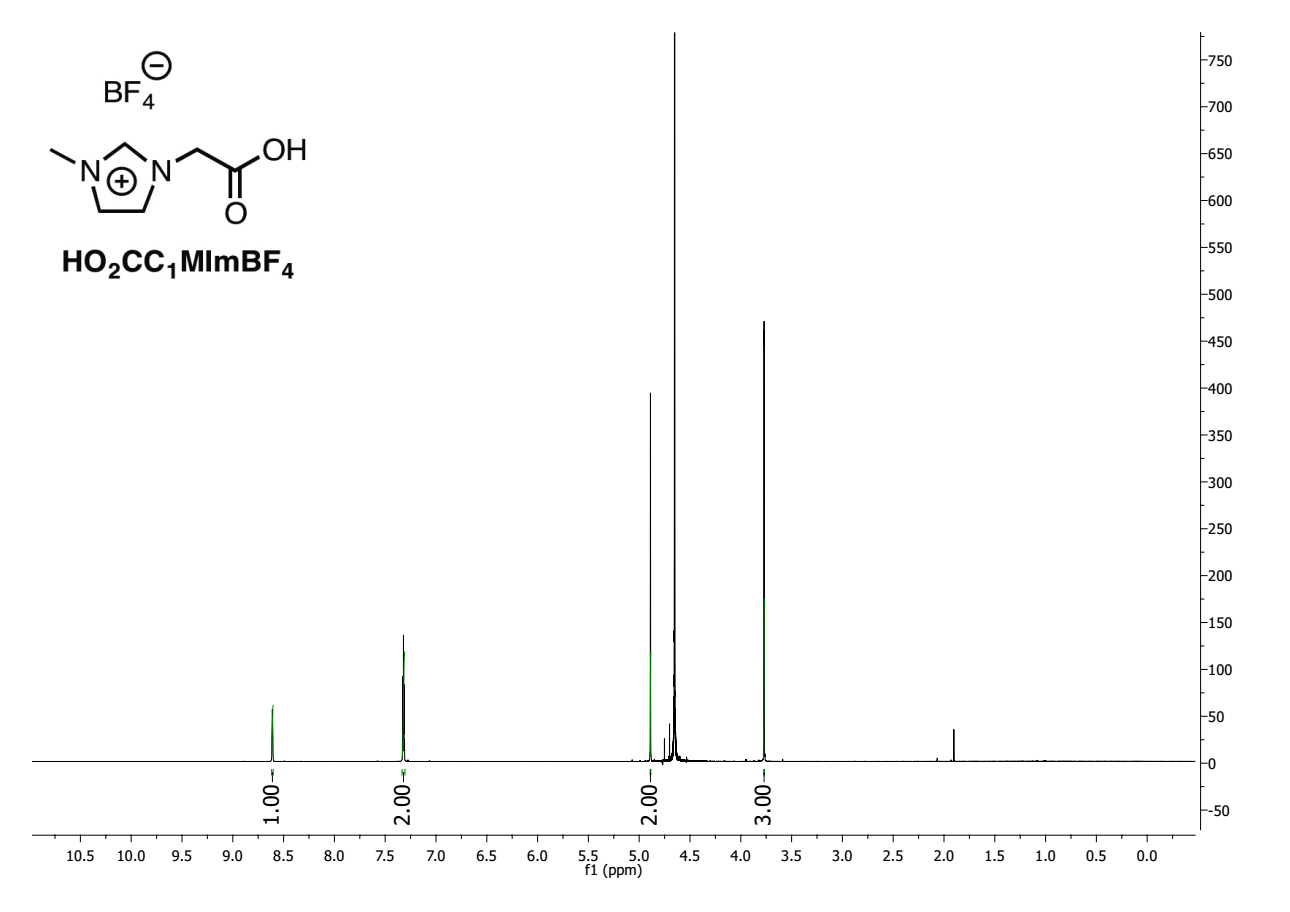


**Figure S33.** ^1^H NMR spectrum of **HO_2_CC_1_MImBF_4_** (400 MHz, D_2_O).


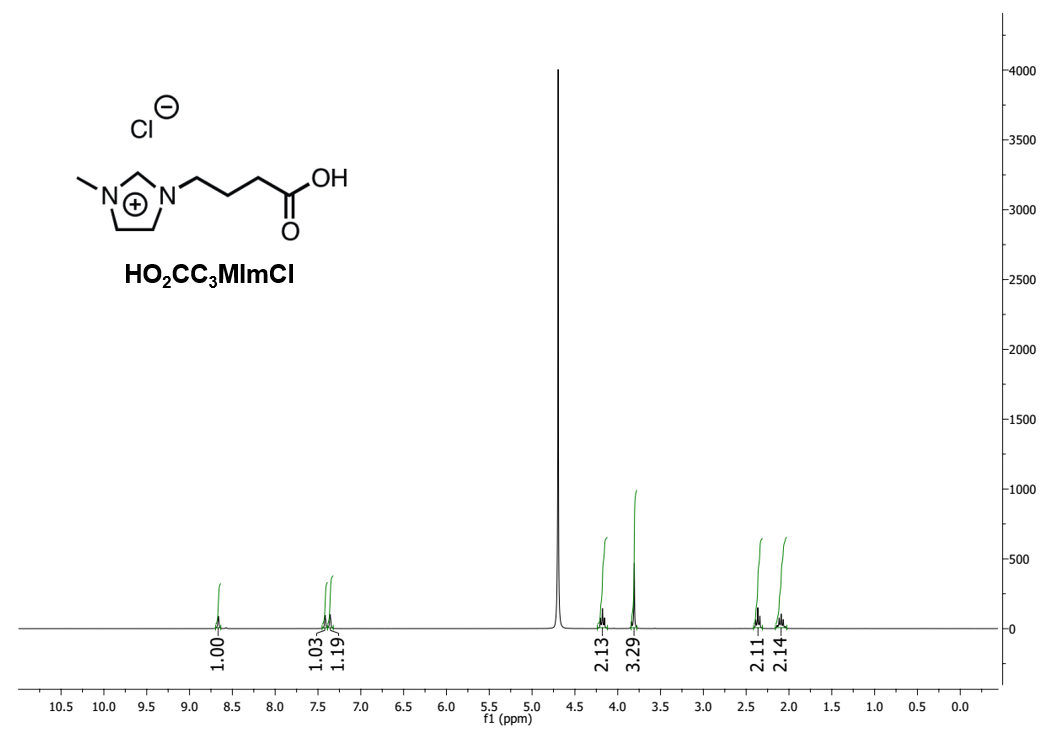


**Figure S34.** ^1^H NMR spectrum of **HO_2_CC_3_MImCl** (400 MHz, D_2_O).


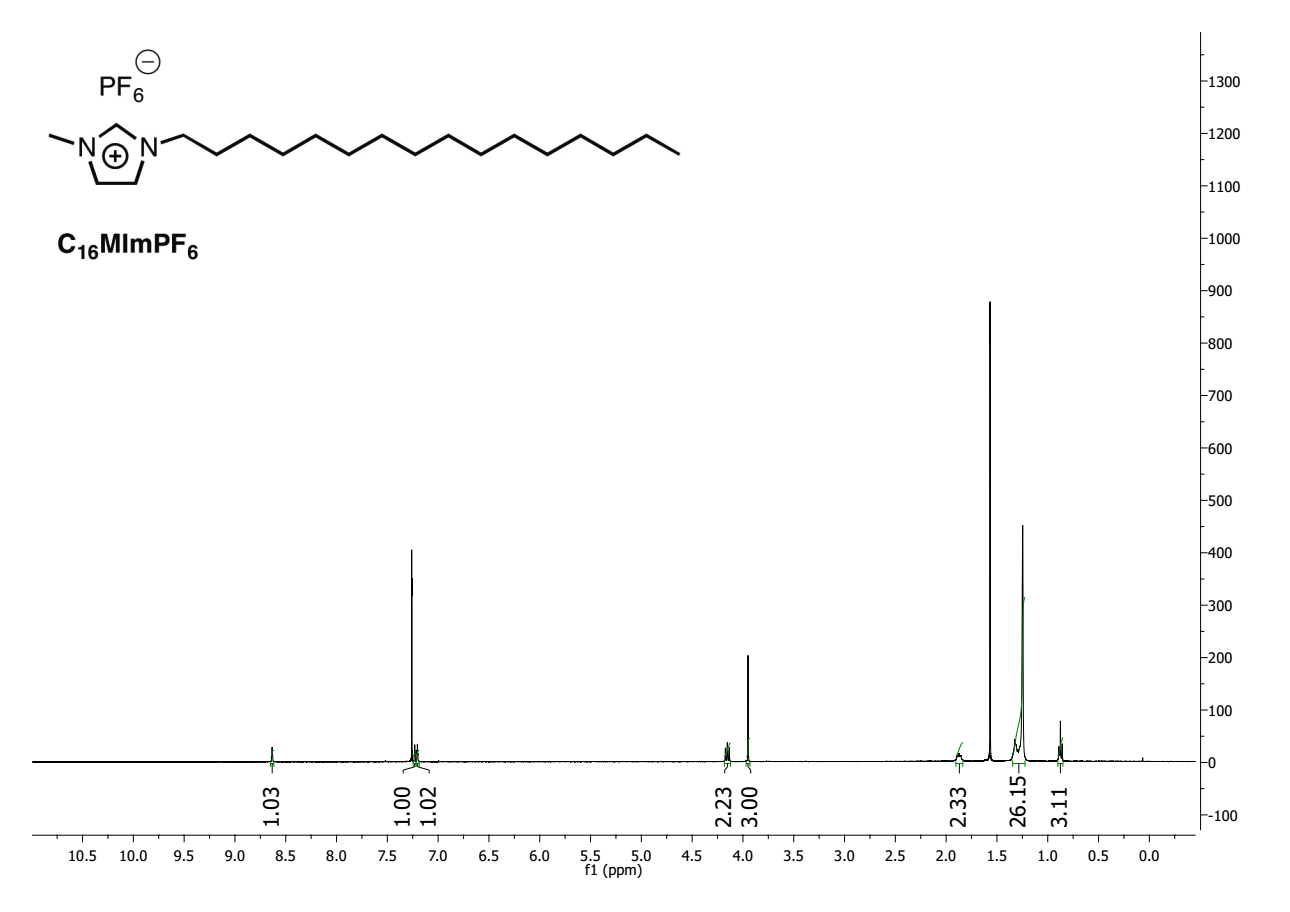


**Figure S35.** ^1^H NMR spectrum of **C_16_MImPF_6_** (400 MHz, CDCl_3_).


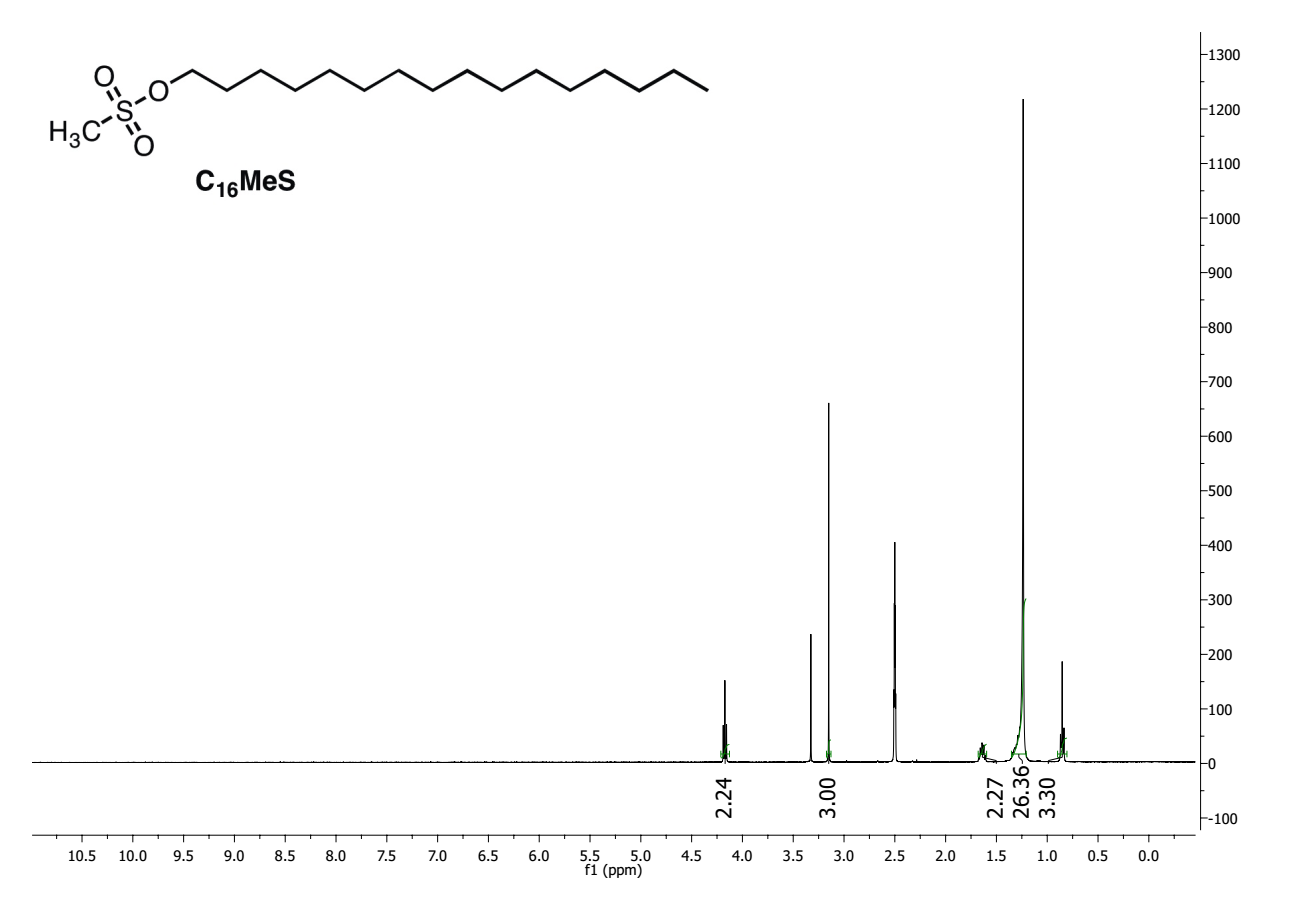


**Figure S36.** ^1^H NMR spectrum of *n*-hexadecyl methanesulfonate (400 MHz, DMSO-d6).


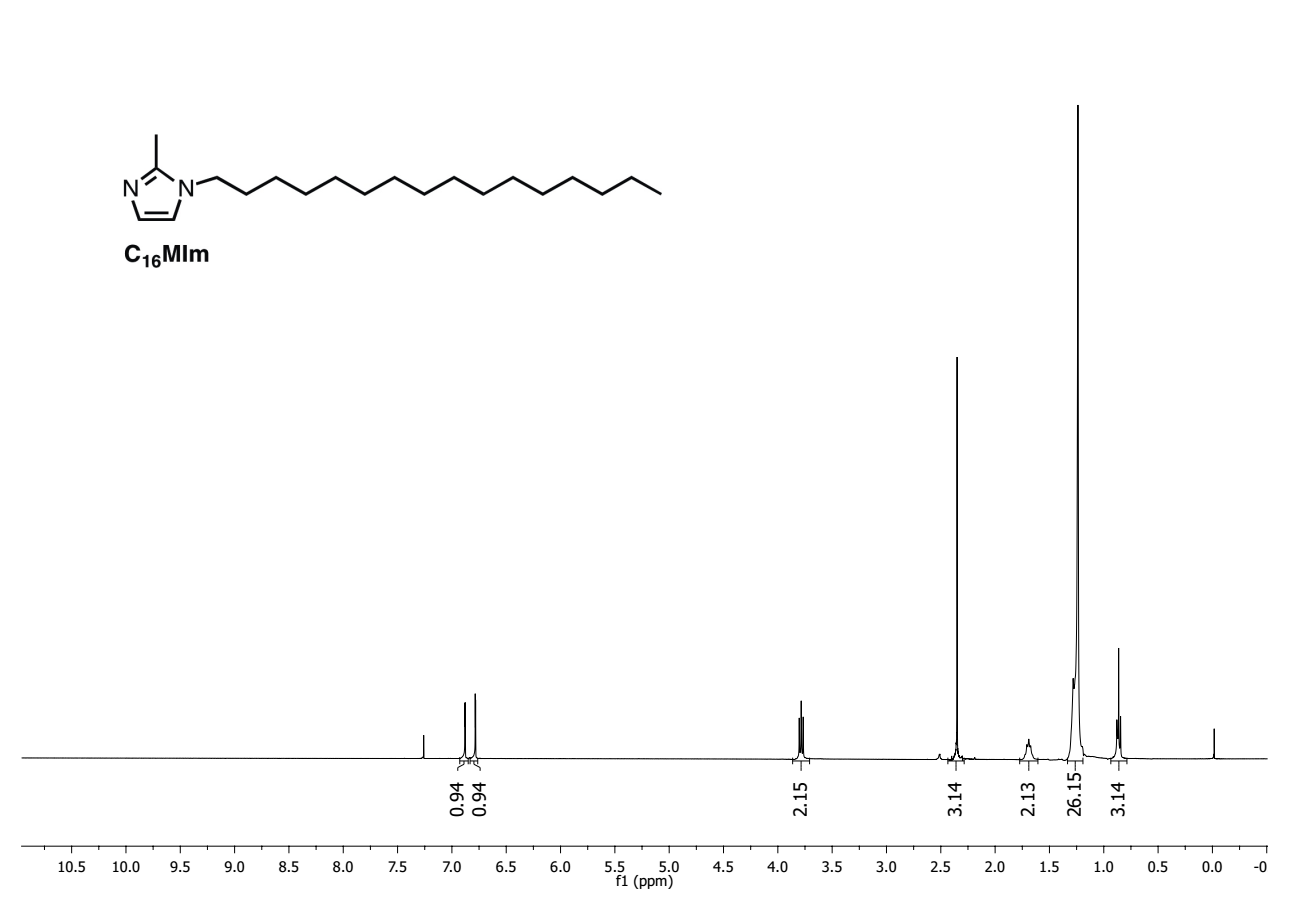


**Figure S37.** ^1^H NMR spectrum of **C_16_MIm** (400 MHz, CDCl_3_).





**Figure S38 - Leishmanicidal activity of imidazolium salts, C_16_Im and C_16_PyrCl on promastigote forms of *L. amazonensis* (A) and *L. infantum chagasi* (B).** Promastigotes in the stationary phase (3 × 10^6^ cells/well) were treated or not with the compounds (0.1 to 100 μM) and the viability determined using the MTT assay after 48 h of incubation. The activity of each compound was compared with control samples incubated with culture medium containing less than 0.05% of polysorbate 80 (Sigma-Aldrich) or amphotericin B at 5 µM (100 % mortality; Sigma-Aldrich) used as reference antileishmanial drug. Data are expressed as means ± SEM (n = 5) and are representative of three independent experiments. The One-way ANOVA test followed by the Bonferroni’s test were applied. **p < 0.01, ***p < 0.001 and ****p < 0.0001 when compared with the medium group (100% viability)





**Figure S39.** ***In vitro* cytotoxicity of imidazolium salts, C_16_Im and C_16_PyrCl towards macrophages (A) and red blood cells (B)*.*** 1 × 10^5^ RAW cells were incubated or not with the compounds at 5 to 200 µM, and viability was determined by MTT assay. Triton X-100 1% was used as positive control **(A)**. Human erythrocytes were incubated with PBS (negative control), Triton X-100 1% (positive control) or IS at 1 to 100 µM for 60 min at room temperature. Hemolysis rates were estimated by measuring the absorbance of supernatants at 540 nm (**B**). Data are expressed as means ± SEM (n = 5) and are representative of three independent experiments. The One-way ANOVA test followed by the Bonferroni’s test were applied. *p < 0.05, ***p < 0.001 and ****p < 0.0001 when compared with the medium group (to macrophage; 100% viability) or with the PBS group (to erythrocytes; 0% hemolysis)
